# Targeted inhibition of the PI3K/AKT/mTOR pathway by (+)-anthrabenzoxocinone induces cell cycle arrest, apoptosis, and autophagy in non-small cell lung cancer

**DOI:** 10.1186/s11658-024-00578-6

**Published:** 2024-04-23

**Authors:** Xiao-Qian Li, Xiao-Ju Cheng, Jie Wu, Kai-Feng Wu, Tie Liu

**Affiliations:** 1 The Third Affiliated Hospital of Zunyi Medical University, The First People’s Hospital of Zunyi), Scientific Research Center, Guizhou, 563002 People’s Republic of China; 2 Yunnan Characteristic Plant Extraction Laboratory, Key Laboratory of Medicinal Chemistry for Natural Resource, Key Laboratory of Medicinal Chemistry for Natural Resource, Ministry of Education and Yunnan Province, School of Chemical Science and Technology, Yunnan University, Kunming, 650091 People’s Republic of China

**Keywords:** Anthrabenzoxocinones, Non-small cell lung cancer, Cell cycle arrest apoptosis, Autophagy, PI3K/AKT/mTOR pathway, ROS

## Abstract

Non-small cell lung cancer (NSCLC), characterized by low survival rates and a high recurrence rate, is a major cause of cancer-related mortality. Aberrant activation of the PI3K/AKT/mTOR signaling pathway is a common driver of NSCLC. Within this study, the inhibitory activity of (+)-anthrabenzoxocinone ((+)-ABX), an oxygenated anthrabenzoxocinone compound derived from Streptomyces, against NSCLC is demonstrated for the first time both in vitro and in vivo. Mechanistically, it is confirmed that the PI3K/AKT/mTOR signaling pathway is targeted and suppressed by (+)-ABX, resulting in the induction of S and G2/M phase arrest, apoptosis, and autophagy in NSCLC cells. Additionally, the augmentation of intracellular ROS levels by (+)-ABX is revealed, further contributing to the inhibition of the signaling pathway and exerting inhibitory effects on tumor growth. The findings presented in this study suggest that (+)-ABX possesses the potential to serve as a lead compound for the treatment of NSCLC.

**Graphical abstract:**

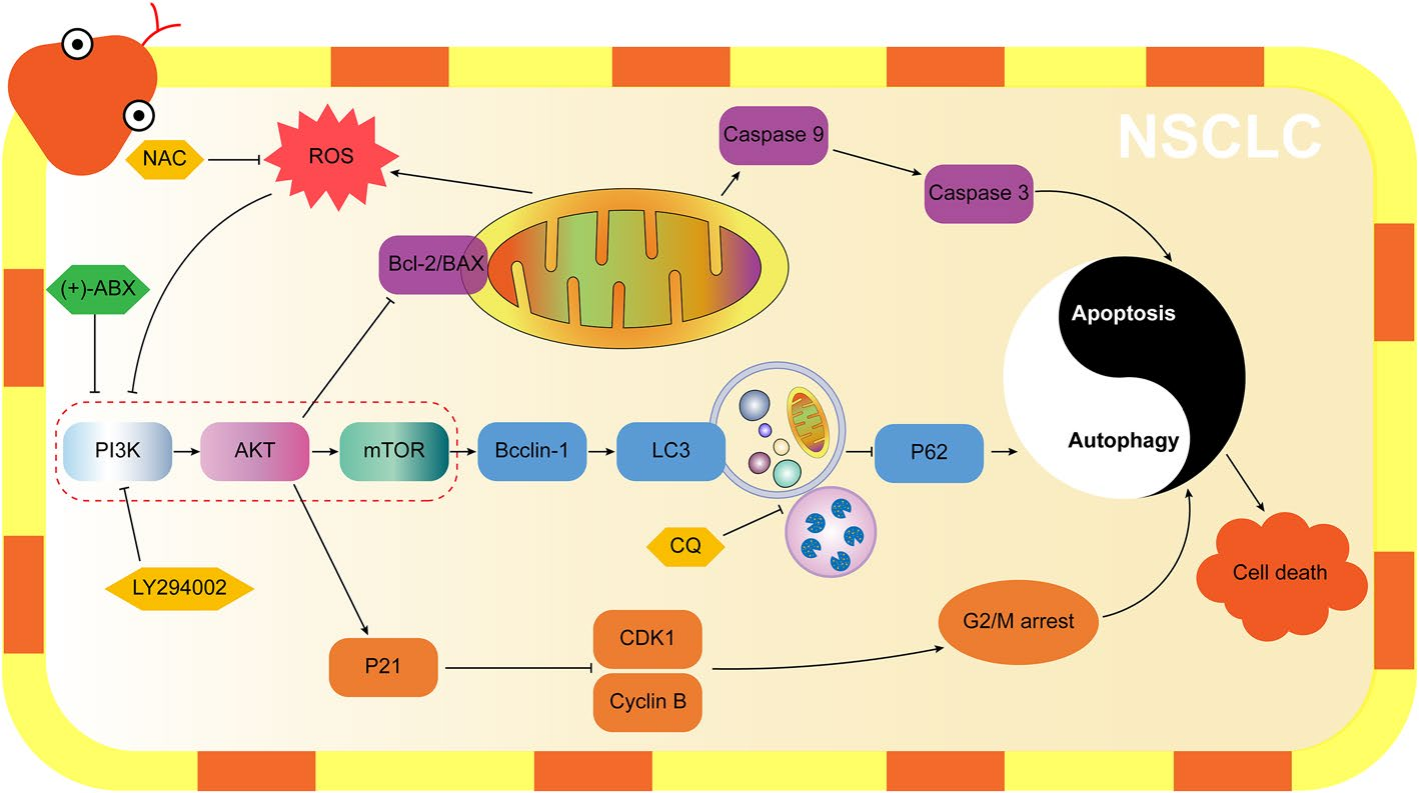

**Supplementary Information:**

The online version contains supplementary material available at 10.1186/s11658-024-00578-6.

## Introduction

Lung cancer, characterized by high incidence and mortality rates, is a leading cause of cancer-related death globally, resulting in an annual death toll exceeding 1.59 million individuals and accounting for 19.4% of cancer-related deaths [1]. Notably, non-small cell lung cancer (NSCLC) constitutes over 85% of all lung cancer cases. Due to the lack of effective screening measures and the occurrence of metastasis in the early stages of the lung cancer natural history, patients are frequently diagnosed in advanced stages. Advanced NSCLC is regarded as a single disease entity necessitating systemic treatments, with a notably low overall cure rate and a high relapse rate. Its 5-year survival rate is a mere 17.8%, remarkably low even among all cancers, reflecting a challenging scenario [2]. Despite the development of several novel drugs such as bevacizumab and pemetrexed for NSCLC treatment over the past decade, the complexity of etiology, heterogeneity among subtypes, and tumor heterogeneity have collectively contributed to the absence of desired treatment outcomes [3]. Currently, immune checkpoint inhibitors (ICIs) have significantly altered the landscape of first-line treatment for advanced NSCLC. Both monotherapy with ICIs and their combination with chemotherapy are recommended strategies according to the National Comprehensive Cancer Network (NCCN) guidelines [4, 5]. The population benefiting solely from immune therapy is limited, and individuals for whom immune therapy proves ineffective, shows poor efficacy, or develops resistance often need to resort to chemotherapy or a combination of immunotherapy and chemotherapy [6]. Combining chemotherapy with immunotherapy holds potential advantages, such as inducing immunogenic cell death, activating adaptive immune responses, and modulating the tumor microenvironment. Chemotherapy may reduce the inhibition of tumor-infiltrating lymphocytes by the tumor microenvironment, thereby enhancing the effectiveness of immunotherapy [7]. Clinical studies like KEYNOTE-189, KEYNOTE-407, and IMpower150 indicate that combining ICIs with chemotherapy yields a higher objective response rate, complementing the efficacy of immune monotherapy [8–10]. Consequently, the ongoing development of more efficient and less toxic chemotherapy drugs remains imperative.

 Food and Drug Administration (FDA)-approved chemotherapy drugs encompass alkylating agents, antimetabolites, antitumor antibiotics, kinase inhibitors, topoisomerase inhibitors, mitotic inhibitors, and corticosteroids [11, 12]. Antitumor antibiotics derived from microorganisms are characterized by high efficiency and a broad spectrum, making them a significant component of clinical chemotherapy [13]. Bleomycin, actinomycin, doxorubicin, and streptozotocin are all classified as antitumor antibiotics and are extensively employed in the treatment of various cancers [14–17]. In the context of NSCLC treatment, doxorubicin and actinomycin are also commonly used medications [18, 19]. The ongoing exploration for novel antitumor antibiotics from microorganism sources holds great promise.

*Streptomyces* FJS31-2, a unique habitat strain isolated from the soil of Fanjing Mountain National Nature Reserve in Tongren City, Guizhou Province, China, is preserved at the China General Microbiological Culture Collection Center under accession number CGMCC 4.7321. This strain is capable of producing a variety of antibiotics, including zunyimycins effective against Gram-positive drug-resistant bacteria [20], as well as milbemycins that can reverse cisplatin resistance in tumors [21]. (+)-Anthrabenzoxocinone ((+)-ABX), a type II polyketide compound, was isolated from the fermentation product of this strain [22]. Originally discovered by Kojiri, Katsuhisa, and Lam, Y. K. Tony from *Streptomyces violaceusniger* and *Actinomyces* MA7150, it was initially named BE 24566B [23] and L 755805 [24], respectively. Subsequently confirmed as the same compound [25], it demonstrated activity against drug-resistant Gram-positive bacteria, including Methicillin-resistant *Staphylococcus aureus* (MRSA). In our preliminary antitumor activity screening, we observed that (+)-ABX exhibited significant inhibitory activity against tumor cells, particularly NSCLC cell lines (Additional file 1: Fig. S1). To date, the antitumor activity of (+)-ABX has not been reported in literature. Conducting systematic antitumor research on it is highly necessary, as it holds promise for providing new lead compounds for NSCLC treatment.

Evading growth suppressors and resisting cell death are the most significant characteristics of cancer cells. Therefore, inhibiting the growth of tumor cells and targeting programmed cell death are the most important strategies for fighting cancer [26]. In particular, the effectiveness of two types of programmed death, apoptosis and autophagy, in combating various types of malignancies has been proved by a large number of evidences [27]. Clinical research related to NSCLC has also indicated that overexpression of antiapoptotic genes and dysregulation of autophagy are significant factors contributing to limited chemotherapy efficacy [28]. Dysregulated cell growth and division are fundamental hallmarks of cancer. Inhibiting cancer cell proliferation ultimately revolves around influencing the cell cycle’s progression. The ability to effectively block the tumor cell cycle is an essential criterion for evaluating antitumor drugs [29]. Dysregulation of reactive oxygen species (ROS) generation is a common feature of cancer cells, and ROS plays a pivotal role in regulating cancer progression. Numerous studies have indicated that ROS plays a regulatory role in pathways related to apoptosis, autophagy, and the cell cycle. Clarifying how drugs mechanistically mediate antitumor effects through ROS will be pivotal in unlocking the potential of ROS-targeted cancer therapy [30].

In this study, the first assessment of the inhibitory activity of (+)-ABX, against NSCLC cell lines is conducted. Additionally, the impact of (+)-ABX on critical phenotypes of tumor drug therapy, including cell cycle arrest, apoptosis, autophagy, and oxidative stress, was investigated. To unravel the possible molecular mechanisms underlying these effects and their interconnections, transcriptomic analysis was employed to systematically pinpoint the signaling pathways that (+)-ABX might modulate. Furthermore, molecular docking techniques were utilized to assess potential target sites. These findings were further validated through combinatorial application with various inhibitors. Moreover, the validation of (+)-ABX’s safety and efficacy in vivo was extended through nude mouse xenograft models and immunohistochemical experiments.

## Materials and methods

### Chemicals

(+)-ABX was prepared from the fermentation product of *Streptomyces* FJS31-2 in our laboratory. Chloroquine (CQ) was obtained from Solarbio (Beijing, China), while LY294002 and *N*-acetyl-l-cysteine (NAC) were acquired from MedChemExpress (Shanghai, China).

### Cell lines and cell culture

The human non-small cell lung cancer cell lines A549 (cat. no. CL-0016), NCI-H1299 (cat. no. CL-0165), and the human squamous lung cancer cell line NCI-H226 (cat. no. CL-0396) were sourced from the Scientific Research Center of the First People’s Hospital of Zunyi (China). These cell lines were cultivated in RPMI-1640 medium (Gibco, Australia) supplemented with 10% fetal bovine serum (FBS, Gibco, Australia) under a 5% CO_2_ atmosphere at 37 °C. The cells were passaged three times to reach a stable growth state before being used for the experiments.

### Cell viability assay

The cytotoxicity of (+)-ABX was assessed against A549, H1299, and H226 cells using the CCK-8 method. Briefly, a 100 μL cell suspension (5 × 10^4^ cells/mL) was seeded into 96-well microtiter plates with six replicate wells per group. After 24 h of incubation at 37 °C, 5% CO_2_, and saturated humidity, the compound was introduced. Different concentrations of (+)-ABX (ranging from 0 to 256 μM) were then added to the plates, followed by a 24-h incubation period. After discarding the liquid from the 96-well plate, each well was rinsed with phosphate-buffered saline (PBS). Next, 90 μL of culture medium and 10 μL of CCK-8 solution (Solarbio, Beijing, China) were added to each well, and the plate was further incubated for an additional 4 h. The absorbance at 490 nm (A) was measured using a microplate reader, and the percentage of inhibition was calculated. The formula for calculating percent cell viability is as follows:

$${\text{Percent Cell Viability}}\, = \,\left[ {\left( {\text{Treatment Group OD Value - Blank Group OD Value}} \right)/\left( {\text{Control Group OD Value - Blank Group OD Value}} \right)} \right]\, \times \,{1}00\% .$$

### Colony formation assay

Initially, A549, H1299, and H226 cells were exposed to varying concentrations of (+)-ABX (0, 10, 20, and 40 μM) for 24 h. Following this, the medium was aspirated, and the cells underwent two washes with PBS. The cells were then harvested, reseeded in 6-well plates at a density of 2000 cells per well, and the culture medium was renewed every 2 days. After a 14-day incubation period, the resulting colonies were fixed with 4% paraformaldehyde (Solarbio, Beijing, China) for 15 min, followed by staining with 0.1% crystal violet (Solarbio, Beijing, China) for 20 min. Colonies containing more than ten cells were quantified under a microscope (Olympus). The presented data represent the average of three independent experiments.

### Wound healing assay

The wound healing assay was conducted as previously outlined [31]. A549, H1299, and H226 cells were seeded into 6-well plates at a density of 10 × 10^5^ cells per well. Once a monolayer of confluent cells was established, a wound of approximately 0.5 mm width was generated using a sterile micropipette tip (200 μL), followed by gentle rinsing with PBS twice. Subsequently, the cells were exposed to various concentrations of (+)-ABX (0, 10, 20, and 40 μM) for 24 h. Images documenting the progression of wound healing were captured at 12-h intervals over a 48-h period using an inverted microscope (Olympus). The width of the wound, defined by the gap between the edges of the cell-free area, was quantified using Image-J software (NIH, MD).

### Transwell migration and invasion assay

To assess cell migration, A549, H1299, and H226 cells in logarithmic growth phase were seeded into a 6-well plate. Different concentrations of (+)-ABX (0, 10, 20, and 40 μM) were then added for a 24-h incubation period. Subsequently, the cells were digested and adjusted to a concentration of 1 × 10^6^ cells/mL in RPMI-1640 medium supplemented with 0.1% FBS. For the upper chamber of the transwell, 200 μL of the cell suspension was added, while the bottom chamber contained 500 μL of RPMI-1640 medium supplemented with 20% FBS. The transwell chambers were placed in a 37 °C, 5% CO_2_ incubator (Thermo) for 24 h. Following incubation, the culture medium was aspirated, the chambers were washed twice with PBS, and then the cells were fixed with methanol for 20 min. Subsequently, they were stained with 0.1% crystal violet for 20 min, rinsed with water several times, and imaged under a microscope. The migrated cells were counted after gently removing the upper unimmigrated cells using a cotton swab. For the evaluation of cell invasion, the aforementioned cell suspension was added to the transwell upper chamber that had been precoated with Matrigel. The cells were incubated at 37 °C for 5 h. Following incubation, the residual liquid in the chamber was removed, and each well was supplemented with 70 μL of serum-free medium. An additional 30-min incubation at 37 °C was conducted prior to the experiment. The subsequent steps of the operation were identical to those of the cell migration assay.

### Flow cytometry

A549, H1299, and H226 cells were individually seeded into 6-well plates (1 × 10^5^ cells per well) and exposed to (+)-ABX (0, 10, 20, and 40 μM) for 24 h. For apoptosis detection, all cells were collected and processed using the FITC Annexin V Apoptosis Detection Kit (BD) according to the manufacturer’s instructions. The stained cells were washed twice with PBS and analyzed using a flow cytometer (AccuriTM C6 Plus, BD). To analyze the cell cycle, the cells were collected, fixed in 70% ethanol overnight at 4 °C, and then stained with propidium iodide (PI) for 30 min at 37 °C in the dark. Subsequent analysis was performed using a flow cytometer.

### Cell apoptosis assay

Apoptosis was assessed using Hoechst 33,342 staining. A549, H1299, and H226 cell densities were adjusted to 1 × 10^5^ cells/mL, and 100 μL of cell suspension was seeded into each well of a 96-well plate. The cells were cultured in a 37 °C, 5% CO_2_ environment for 24 h. Subsequently, different concentrations of (+)-ABX (0, 10, 20, and 40 μM) were added after the initial 24-h incubation. The medium was discarded, and the cells were washed twice with PBS. Following this, the cells were fixed with 4% paraformaldehyde for 15 min, stained with 100 μL of Hoechst 33,342 solution (10 μg/mL) (Solarbio, Beijing, China), and incubated at 37 °C in a 5% CO_2_ incubator for 30 min. The staining solution was removed, and the cells were washed twice with PBS. Apoptosis was observed under a fluorescence microscope (Olympus), and the images were captured.

### Measurement of mitochondrial membrane potential (MMP)

The cells were exposed to various concentrations of (+)-ABX (0, 10, 20, and 40 μM) for a duration of 24 h. Following this exposure, the culture medium was aspirated, and the cells were subjected to two washes with PBS. Subsequently, 1 mL of JC-1 staining working solution (Solarbio, Beijing, China) was added to each well, and the cells were incubated in a cell culture incubator at 37 °C for 20 min. The culture medium was then discarded, and the cells were washed twice with precooled JC-1 staining buffer. Afterward, 1 mL of PBS was added to each well to resuspend the cells, allowing for observation and image collection using a fluorescent microscope.

### Quantitative real-time polymerase chain reaction

Total RNA was extracted from A549, H1299, and H226 cells treated with various concentrations of (+)-ABX (0, 10, 20, and 40 μM) for 24 h using the RNAiso Plus reagent (Takara, Beijing, China). Subsequently, the extracted total RNA was reverse transcribed into first-strand cDNA using the PrimeScript™ RT Master Mix (Takara, Beijing, China). Real-time PCR amplification was carried out utilizing the Real-Time PCR Detection System (CFX96, Bio-Rad, USA) and the SYBR Premix Ex Taq™ (Takara, Beijing, China). The PCR primers (Additional file 1: Table S1) were designed and synthesized by Sangon Biotech (Shanghai, China). The relative mRNA expression levels were determined using the relative standard curve method with β-actin as a reference.

### Western blot assay

The cells were seeded in 6-well plates and cultured for 24 h prior to treatment with (+)-ABX (0, 10, 20, 40 µM) or inhibitors (CQ, LY294002, and NAC) for an additional 24 h. Following treatment, cells were lysed using RIPA lysis buffer (Solarbio, Beijing, China), and protein concentrations were determined using the BCA assay. Proteins (20 µg per lane) were separated using 12% sodium dodecyl sulfate–polyacrylamide gel electrophoresis (SDS–PAGE) gel (Solarbio, Beijing, China). Subsequently, the gels were transferred onto polyvinylidene fluoride membranes (Millipore, MA), blocked with 5% BSA blocking buffer (Solarbio, Beijing, China) for 2 h, and incubated overnight at 4 °C with primary antibodies. Antibodies against Beclin1, LC3B, SQSTM1/p62, cleaved-caspase 3, mTOR, phospho-mTOR, phospho-PI3K, phospho-Akt, Akt, Bcl2, p21, cyclin B1, and CDK1 were procured from Zenbio (Chengdu, China). Antibodies against BAX, Bcl2, and β-actin were sourced from Solarbio (Beijing, China), while cleaved-caspase 9 was obtained from Affinity Biosciences (Changzhou, China). PI3K and HRP Goat anti-Rabbit IgG secondary antibodies were purchased from Cell Signaling Technology (Beverly, MA). Subsequent to incubation with corresponding secondary antibodies at room temperature for 2 h, the membranes were treated with Immobilon Western Chemiluminescent HRP Substrate (Millipore, MA) and imaged using the BIO-RAD ChemiDoc Imaging System (Hercules, CA).

### Transmission electron microscopy (TEM)

A549 and H1299 cells treated with (+)-ABX at 20 μM for 24 h were collected and subjected to pre-embedding in 1% agarose solution. Subsequently, the samples were fixed in 1% osmium tetroxide at room temperature in the dark for 2 h. After three washes with PBS buffer, each lasting 15 min, a series of increasing ethanol concentrations were employed for dehydration. The samples were then subjected to infiltration, polymerization, and slicing processes to obtain ultrathin sections measuring 60–80 nm. These sections were stained with 2% uranyl acetate saturated alcoholic solution in the absence of light, followed by staining with 2.6% lead citrate solution to avoid exposure to carbon dioxide. Subsequently, the samples were observed for cellular morphology, and the images were captured using a transmission electron microscope (HT7800, Hitachi, Japan).

### RNA-seq and transcriptomic analysis

The H1299 cells were cultured to the logarithmic growth phase. For the (+)-ABX treated groups, the cells were treated with 20 μM and 30 μM of (+)-ABX, respectively, while the control group received an equivalent volume of solvent. Each group had three replicates, and after 24 h, the cells were collected and treated with 1 mL of Trizol reagent individually. The library preparation and transcriptome sequencing experiments were conducted by Beijing Qinge Biotechnology Company (Beijing, China) using the Illumina HiSeq platform. During the analysis of differentially expressed genes, a threshold of fold change ≥ 2 and *P*-value < 0.05 was utilized for screening. Pathway enrichment analysis of differentially expressed genes was carried out using the KEGG database (www.genome.jp/kegg).

### Molecular docking

The three-dimensional conformation of (+)-ABX was optimized using Chem3D 19.0 (CambridgeSoft, USA). The three-dimensional structures of PI3K (1H9O), AKT (2W1C), and mTOR (4DRI) were downloaded from the RSC Protein Data Bank (https://www.rcsb.org) and processed using PyMol (Version 2.6.0a0 Open-Source). Molecular docking studies were conducted using AutoDock Vina (Version 1.5.7) [32], and the docking results were visualized using Discovery Studio 2019 (BIOVIA, USA).

### Measurement of ROS levels

The cells were adjusted to a density of 1 × 10^6^ cells/mL. Subsequently, 500 μL of the cell suspension was inoculated into a 24-well plate and cultured for 24 h. Different concentrations of (+)-ABX (0, 10, 20, and 40 μM) were then added for an additional 24 h. Following the removal of the culture medium, 1 mL of preprepared DCFH-DA fluorescent dye (Solarbio, Beijing, China) was added to each well. The cells were incubated in the incubator for 30 min, after which any excess dye that had not entered the cells was washed away with PBS. Finally, 1 mL of PBS was added to each well for resuspension. The cells were excited with blue light using an inverted fluorescence microscope, and the fluorescence intensity was observed.

### Tumor xenograft models

The animal experiment protocol was approved by the Laboratory Animal Welfare and Ethical Committee of Zunyi Medical University (ZMU21-2303–105). SPF-grade male BALB/c-nu nude mice (4–5 weeks old) were obtained from Beijing HFK Bioscience CO., Ltd (SCXK J2019-0008). A volume of 100 μL of A549 cell suspension (1 × 10^6^ cells/mL) was subcutaneously inoculated on the back of nude mice. The mental state of the mice was observed every two days, and body weight and tumor volume (0.5 × L × W^2^, where L is length and W is width) were recorded. After 10 days, when the tumor volume reached approximately 60–80 mm^3^, the nude mice were randomly divided into two groups: a control group and a (+)-ABX treatment group (2 mg/kg), each containing six mice. The treatment group received intraperitoneal administration of 200 μL of (+)-ABX (2 mg/kg) every other day for 28 days, while the control group received intraperitoneal injections of an equal volume of normal saline. The experiment was terminated after 30 days, at which point the nude mice were euthanized by cervical dislocation. The tumors and major organs (liver, lung, and kidney) were removed, weighed, recorded, and subsequently fixed with 10% formalin for histopathology and immunology evaluation.

### Histopathology and immunohistochemistry

After the in vivo experiment, subcutaneously transplanted tumors, liver, kidney, and lung samples from each group of nude mice were subjected to hematoxylin and eosin (H&E) staining to assess the potential organ toxicity of (+)-ABX. The procedure was carried out as outlined below. The tissues were fixed in 10% formalin, dehydrated using varying concentrations of ethanol, paraffin-embedded, and subsequently sectioned. The sections were dewaxed, hydrated, stained with hematoxylin for 20 min, water-rinsed, differentiated with differentiation medium for 30 s, water-soaked for 15 min, stained with eosin for 2 min, water-soaked for 5 min, dehydrated, and sealed with neutral balsam. The images were acquired under a microscope. For immunohistochemical staining (IHC), the slides were incubated with antibodies (Ki-67, cleaved-caspase 3, cleaved-caspase 9, BAX, AKT, LC3A, and Beclin1) at 4 °C overnight, followed by incubation with secondary antibodies at room temperature for 2 h. Subsequently, the slides were mounted using neutral balsam, and optical microscope images were captured. Antibodies Ki67 was sourced from ABclonal (Wuhan, China), LC3A from Zenbio (Chengdu, China), and BAX and cleaved-caspase 3 from Proteintech (Wuhan, China). The HRP-Polymer anti-Mouse/Rabbit IHC Kit was acquired from MXB (Fuzhou, China), and the remaining antibodies corresponded to those used in the western blot assay.

### Statistical analysis

All experiments were conducted independently in triplicate, and the outcomes are expressed as mean ± standard deviation (SD). Statistical evaluations were performed using SPSS 29.0 (IBM Corp., Armonk, NY) and GraphPad Prism 8.0 statistical software (GraphPad, La Jolla, CA). Group comparisons were assessed utilizing one-way analysis of variance (ANOVA) and Student’s *t*-test. A significance threshold of *P* < 0.05 denoted statistical significance and is denoted by * or △; ** or ^△△^*P* < 0.01, and *** or ^△△△^*P* < 0.001. A *P*-value exceeding 0.05 indicated nonsignificance (ns).

## Results

### Effective inhibition of proliferation, migration, and invasion in NSCLC cell lines by (+)-ABX

**Fig. 1:**
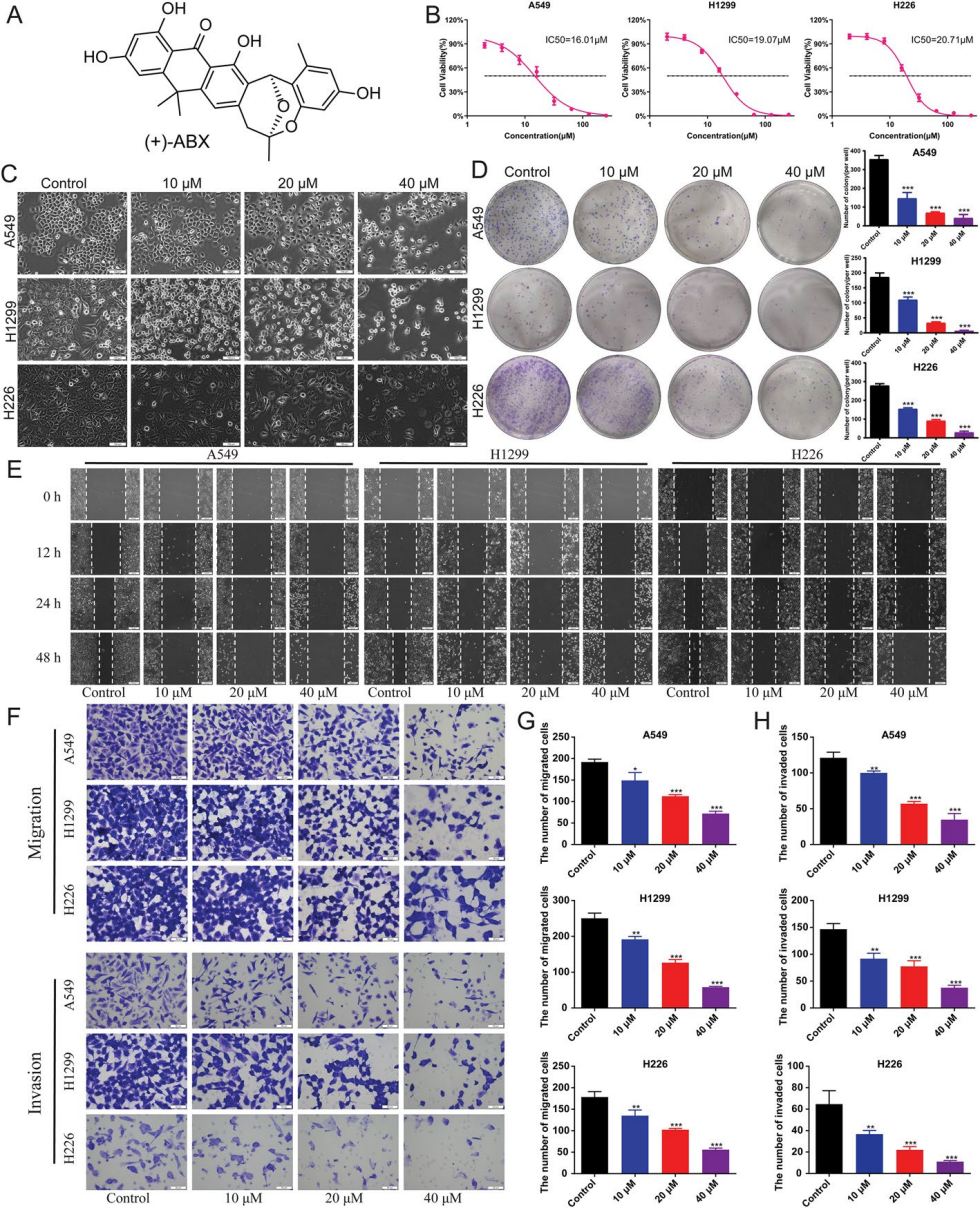
Inhibition of proliferation, migration, and invasion of NSCLC cell lines by (+)-ABX. **A** Chemical structure of (+)-ABX. **B** Determination of IC_50_ values using CCK-8 assay. **C** Morphological changes in cells treated with various concentrations of (+)-ABX for 24 h. **D** Colony formation of NSCLC cells after treatment with different concentrations of (+)-ABX. Colony-forming ability was quantified by counting colonies per well. **E** Measurement of wound healing in NSCLC cells after (+)-ABX treatment. **F** Assessment of transwell migration and invasion of NSCLC cells after (+)-ABX treatment. **G** Quantitative analysis of wound healing, transwell migration, and invasion assays. The bar graphs represent mean ± SD of at least three independent experiments; *P < 0.05, **P < 0.01, and ***P < 0.001 compared with the control group

Initially, the inhibitory effects of (+)-ABX on NSCLC cell lines were observed using optical microscopy (Fig. 1A). After 24 h of treatment with various concentrations of (+)-ABX, noticeable changes in cell morphology were observed in all three NSCLC cell lines compared with the control group. Additionally, an increase in floating dead cells and a reduction in cell numbers were noted with increasing (+)-ABX concentrations (Fig. 1C). Furthermore, the ability of these cell lines to form colonies was diminished as the concentration of (+)-ABX increased (Fig. 1D and Additional file 1: Fig. S2). The IC_50_ (half maximal inhibitory concentration) values of (+)-ABX for A549, H1299, and H226 cell lines were determined as 16.01 μM, 19.07 μM, and 20.71 μM, respectively, using the CCK-8 assay (Fig. 1B). These results collectively indicate the concentration-dependent inhibitory effect of (+)-ABX on the proliferation of these three NSCLC cell lines. The outcomes of the wound healing assay (Fig. 1E) and transwell assay demonstrated a gradual reduction in migration and invasion areas, as well as cell numbers, for the three NSCLC cell lines with increasing (+)-ABX concentrations (Fig. 1F–H and Additional file 1: Figs. S3 and S4). This provides evidence that (+)-ABX effectively suppresses the migration and invasion of NSCLC cells in a dose-dependent manner.

### The cell cycle of NSCLC cells is arrested in G2/M phase and S phase by (+)-ABX

**Fig. 2:**
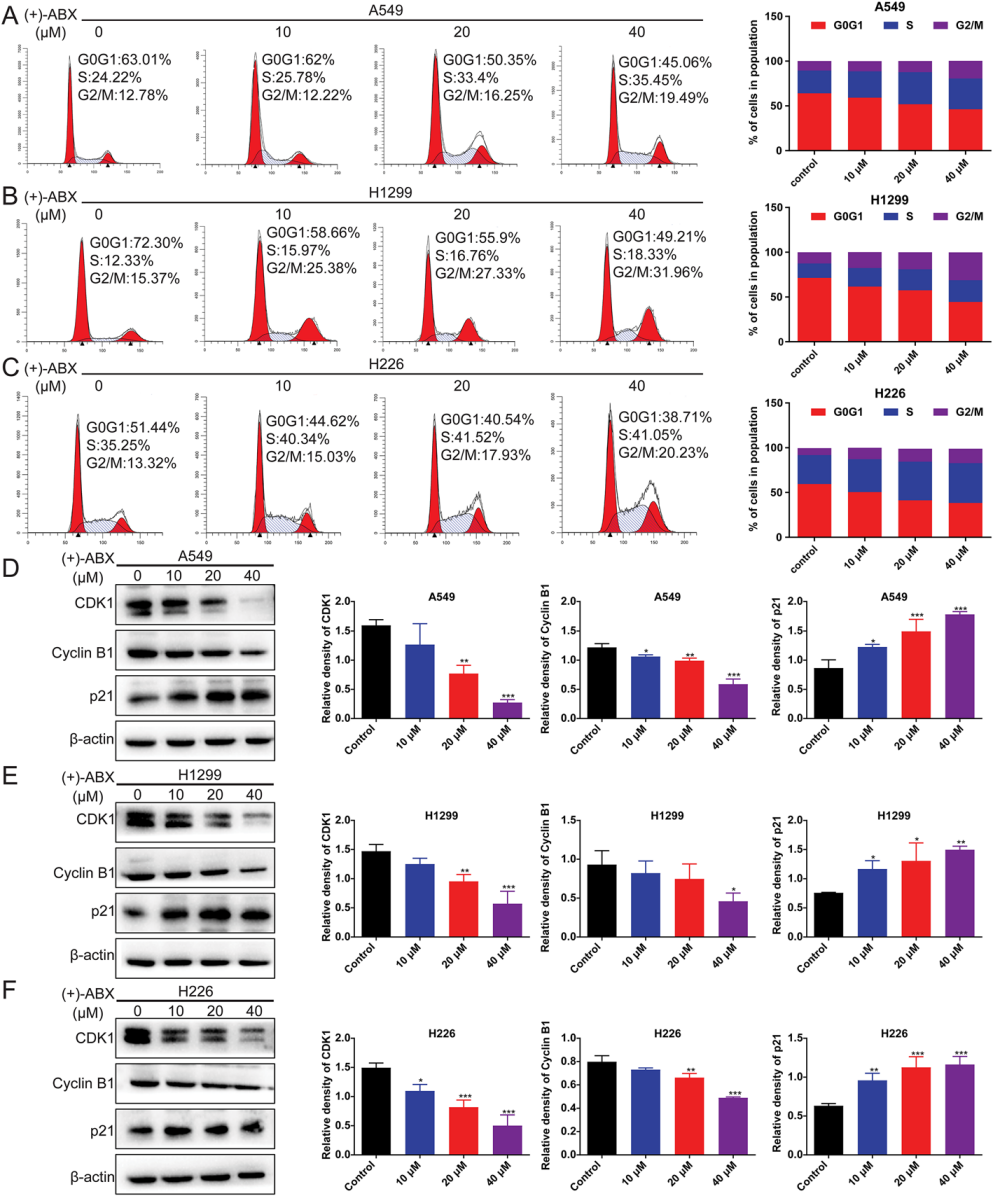
Induction of S phase and G2/M phase arrest by (+)-ABX in NSCLC cells. **A** Flow cytometric analysis of cell cycle distribution in A549 cells. **B** Flow cytometric analysis of cell cycle distribution in H1299 cells. **C** Flow cytometric analysis of cell cycle distribution in H226 cells. **D**–**F** Expression levels of G2/M phase-related proteins in the three NSCLC cell lines assessed through western blotting. Data are presented as means ± SD. *P < 0.05 and **P < 0.01, and ***P < 0.001 compared with the control group. The data were obtained from a minimum of three independent experiments

Previous findings have initially confirmed the inhibitory activity of (+)-ABX in these three NSCLC cell lines, though the underlying biological mechanisms remain unclear. To investigate whether (+)-ABX affects the cell cycle of these cell lines, flow cytometry was employed to analyze the cell cycle distribution of the experimental group treated with (+)-ABX in comparison with the control group. In the experimental group subjected to (+)-ABX intervention, a notable increase in the proportion of cells in the G2/M and S phases was observed (Fig. 2A–C and Additional file 1: Fig. S5). Specifically, in A549 cells, the proportions of cells in the G2/M phase for each group (0, 10, 20, and 40 μM) were 10.6 ± 4.24%, 11.44 ± 3.04%, 12.28 ± 3.6%, and 19.75 ± 1.69%, respectively, while the proportions of cells in the S phase were 25.4 ± 1.53%, 29.39 ± 4.6%, 35.9 ± 3.3%, and 29.02 ± 8.4%, respectively. In H1299 cells, the proportions of cells in the G2/M phase for each group were 12.55 ± 2.66%, 17.66 ± 3.11%, 19.01 ± 3.25%, and 31.12 ± 0.78%, respectively, while the proportions of cells in the S phase were 16.19 ± 3.67%, 20.76 ± 4.01%, 23.55 ± 2.13%, and 24.3 ± 2.12%, respectively. In H226 cells, the proportions of cells in the G2/M phase for each group were 7.76 ± 3.24%, 12.7 ± 3.24%, 14.59 ± 3.74%, and 16.04 ± 2.67%, respectively, while the proportions of cells in the S phase were 32.2 ± 2.12%, 36.77 ± 3.45%, 44.23 ± 5.4%, and 43.51 ± 5.3%, respectively. In summary, (+)-ABX induces G2/M and S phase arrest in these cells, with the effect being predominantly dose-dependent and notably more pronounced in G2/M phase. Furthermore, the aforementioned conclusion was reinforced by examining the expression of G2/M phase-related proteins, where (+)-ABX dose-dependently upregulated p21 expression while downregulating CDK1 and cyclin B expression (Fig. 2D–F).

### Induction of apoptosis in NSCLC cells by (+)-ABX

**Fig. 3:**
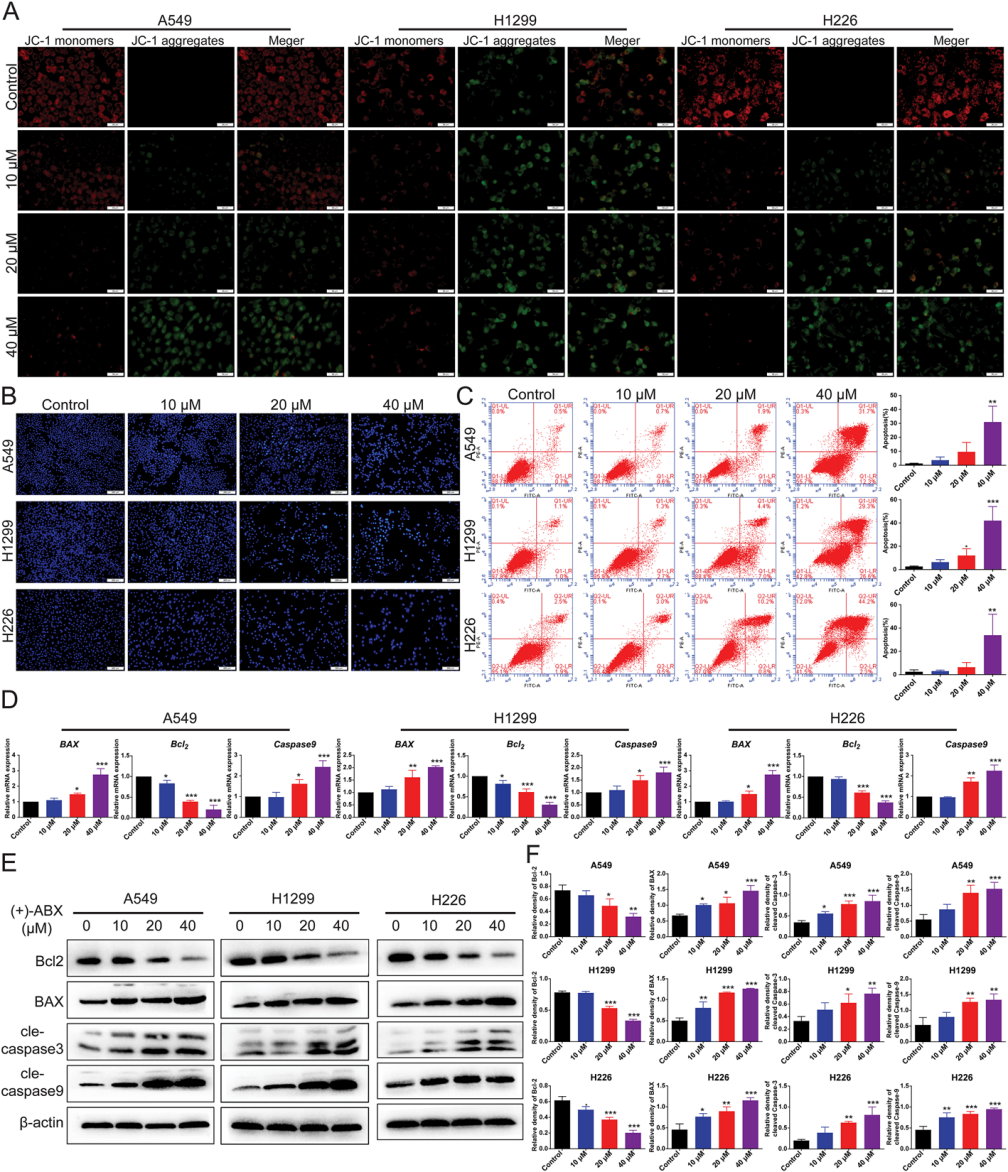
(+)-ABX induces apoptosis in NSCLC cells. **A** Measurement of mitochondrial membrane potential (MMP) in cells treated with (+)-ABX (0, 10, 20, and 40 μM) for 24 h using JC-1 staining. **B** NSCLC cells exposed to various concentrations of (+)-ABX were evaluated for apoptosis through Hoechst33342 staining assays. **C** Flow cytometry-based annexin V/FITC/PI staining was conducted after a 24 h exposure to (+)-ABX to quantify cell apoptosis rates in each phase. **D** Relative mRNA expression of apoptosis-related genes in NSCLC cells treated with (+)-ABX (0, 10, 20, and 40 μM) for 24 h. **E** Cellular exposure to (+)-ABX (0, 10, 20, and 40 μM) for 24 h was followed by western blotting to assess the expression of apoptosis-related proteins. **F** Relative protein expression of apoptosis-related factors. Data were presented as means ± SD. * *P* < 0.05, ** *P* < 0.01, and ***P < 0.001 compared with the control. Information was collected from a minimum of three independent experiments

Apoptosis, a crucial programmed cell death mechanism, is significant in cancer cell biology. Therefore, we investigated whether (+)-ABX could induce apoptosis in the three NSCLC cell lines. Initially, Hoechst 33,342 staining was employed to assess changes in nuclear morphology. In comparison with the control group, cells treated with (+)-ABX exhibited distinct apoptotic changes in nuclear morphology. The nuclei of apoptotic cells appeared densely stained, either in a condensed form or as fragmented clusters, with the apoptotic effect intensifying in line with drug concentration augmentation (Fig. 3B).

Changes in mitochondrial membrane potential (MMP) represent the earliest distinctive events within the apoptotic cascade. As illustrated in Fig. 3A, control group cells showcased JC-1 in polymer form, indicative of higher membrane potential, resulting in red fluorescence. With the augmentation of (+)-ABX concentration, the intensity of red fluorescence within cells gradually waned, while green fluorescence became more pronounced. This shift caused a proportional reduction in the red-to-green fluorescence ratio with the increasing (+)-ABX concentration, thereby indicating a progressive decline in mitochondrial membrane potential. This decline marks an upsurge in the apoptotic rate.

Furthermore, apoptotic rates were determined using flow cytometry. The results exhibited that after exposure to various concentrations of (+)-ABX (0, 10, 20, and 40 μM) for 24 h, the apoptotic rates were as follows: for A549 cells, 1.3 ± 0.17%, 3.7 ± 2.2%, 9.6 ± 6.7%, and 31 ± 11.3%, respectively; for H1299 cells, the corresponding rates were 2.6 ± 0.44%, 6.4 ± 2.2%, 12.1 ± 5.8%, and 42 ± 12.1%; and H226 cells exhibited rates of 2.5 ± 1.67%, 3.03 ± 0.81%, 6.4 ± 4%, and 33.9 ± 18.1%. Notably, as the drug concentration increased, the apoptotic rates of the cells also displayed an ascending trend (Fig. 3C and Additional file 1: Fig. S6).

Furthermore, the mRNA expression of apoptosis-related proteins was analyzed using PCR with reverse transcription (RT–qPCR). It was observed that after treatment with (+)-ABX, there was a downregulation of *Bcl-2* gene expression, accompanied by an upregulation of *BAX* and *caspase 9* gene expressions (Fig. 3D). This trend was further confirmed at the protein level, where the antiapoptotic protein Bcl-2 exhibited reduced expression, while the proapoptotic proteins BAX, cleaved-caspase 3, and cleaved-caspase 9 showed increased expression. Notably, these effects demonstrated a dose-dependent pattern (Fig. 3E, F). In summary, the induction of cellular apoptosis through the mitochondrial pathway constitutes one of the mechanisms through which (+)-ABX combats NSCLC cells.

### (+)-ABX induces autophagy in NSCLC cells and its interplay with apoptosis

**Fig. 4:**
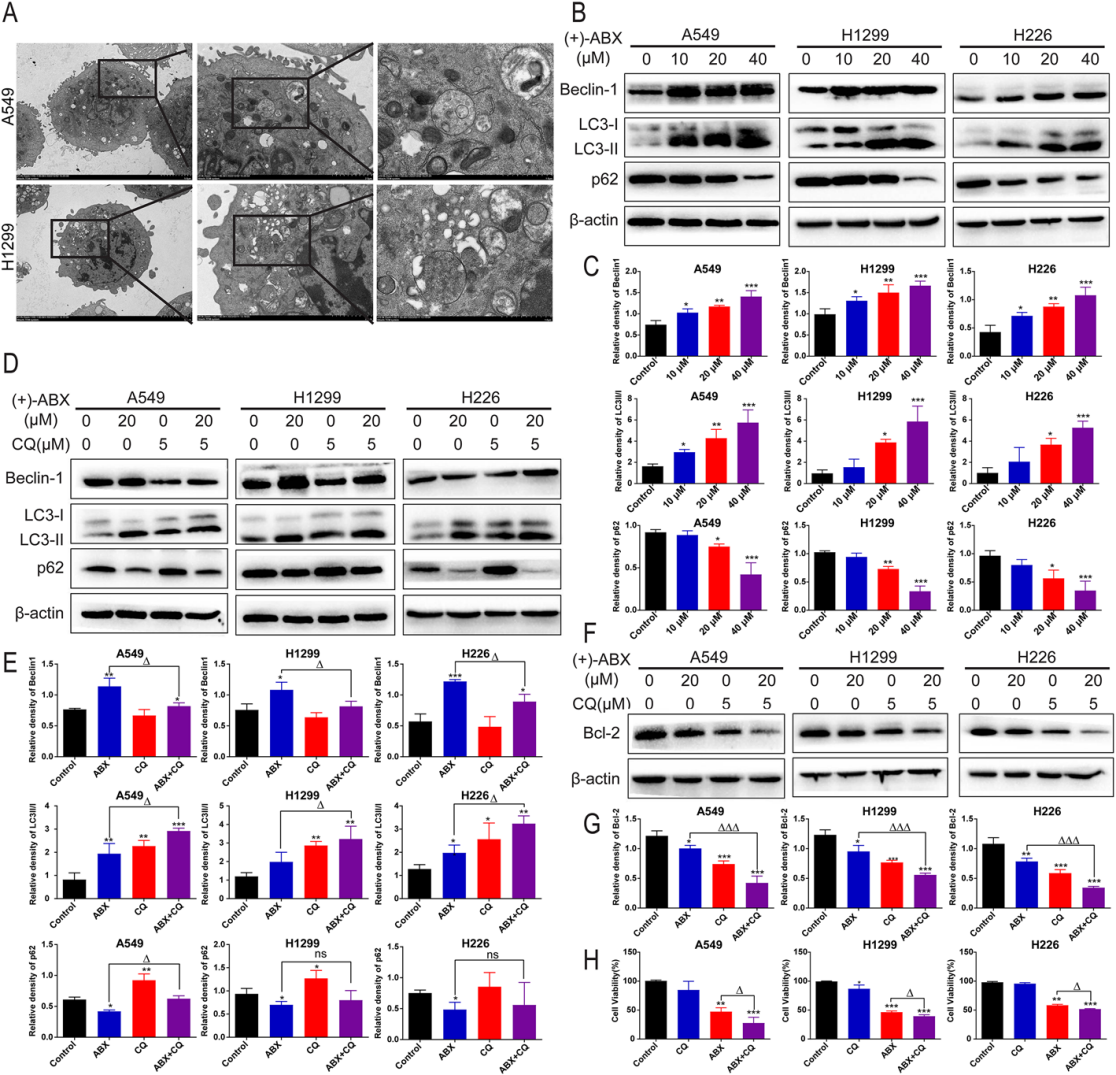
Induction of autophagy by (+)-ABX and the impact of autophagy inhibition on apoptosis and anticancer effects. **A** Observation of cellular autophagic features using TEM. Scale bars, 5 μm, 2 μm, and 1 μm. **B** Western blot analysis of autophagy-related protein expression in cells treated with different concentrations of (+)-ABX. **C** Quantification of the data from panel B. **D** Western blot analysis of changes in autophagy-related protein expression after (+)-ABX treatment with CQ intervention. **E** Quantification of the data from panel D.** F** Western blot analysis of changes in apoptosis-related protein expression after (+)-ABX treatment with CQ intervention. **G** Quantification of the data from panel F. **H** CCK-8 assay measuring the impact of CQ pretreatment on cell viability after (+)-ABX exposure. Data are presented as means ± SD. * or ^△^*P* < 0.05, ** or ^△△^*P* < 0.01, and *** or ^△△△^*P* < 0.001 compared with the control. The results were derived from a minimum of three independent experiments

Autophagy is also a crucial form of programmed cell death that interplays with apoptosis. To ascertain whether (+)-ABX affects NSCLC cells through autophagic mechanisms, we employed transmission electron microscopy (TEM) to observe cells post (+)-ABX treatment. As depicted in Fig. 4A, treated cells exhibited characteristic features of macroautophagy, evident by distinct autophagosomes and autolysosomes. Furthermore, we assessed the expression of several autophagy-associated proteins at the protein level. (+)-ABX dose-dependently upregulated the expression of Beclin-1 and the LC3-II/LC3-I ratio in the three NSCLC cell lines. Simultaneously, (+)-ABX downregulated p62 expression levels (Fig. 4B, C).

Subsequently, we employed the autophagy inhibitor CQ to investigate the relationship between (+)-ABX-induced autophagy and apoptosis in NSCLC cells. CQ hampers the completion of autophagy by inhibiting the fusion of autophagosomes with lysosomes and the degradation of lysosomal proteins. Following cotreatment, the expression level of Beclin-1 decreased to levels comparable to the control group, and the LC3B-II/I ratio was higher compared with when (+)-ABX was used alone. The expression level of p62 was higher than that with (+)-ABX alone, while the antiapoptotic protein Bcl-2’s expression level was further downregulated compared with (+)-ABX alone (Fig. 4D–G). This suggests that CQ obstructed the autophagic flux induced by (+)-ABX in these cells, thus enhancing apoptosis and promoting the apoptotic effects elicited by (+)-ABX. Moreover, the growth of cells in the combination group was significantly more suppressed compared with the (+)-ABX treatment group (Fig. 4H). These findings collectively indicate that (+)-ABX induces substantial macroautophagy in NSCLC cells, and cotreatment with CQ, which impedes autophagy, intensifies apoptosis, enhancing the inhibitory effect of (+)-ABX on these cells.

### Transcriptomic and molecular docking analysis suggests that (+)-ABX may target the PI3K/AKT/mTOR signaling pathway

**Fig. 5:**
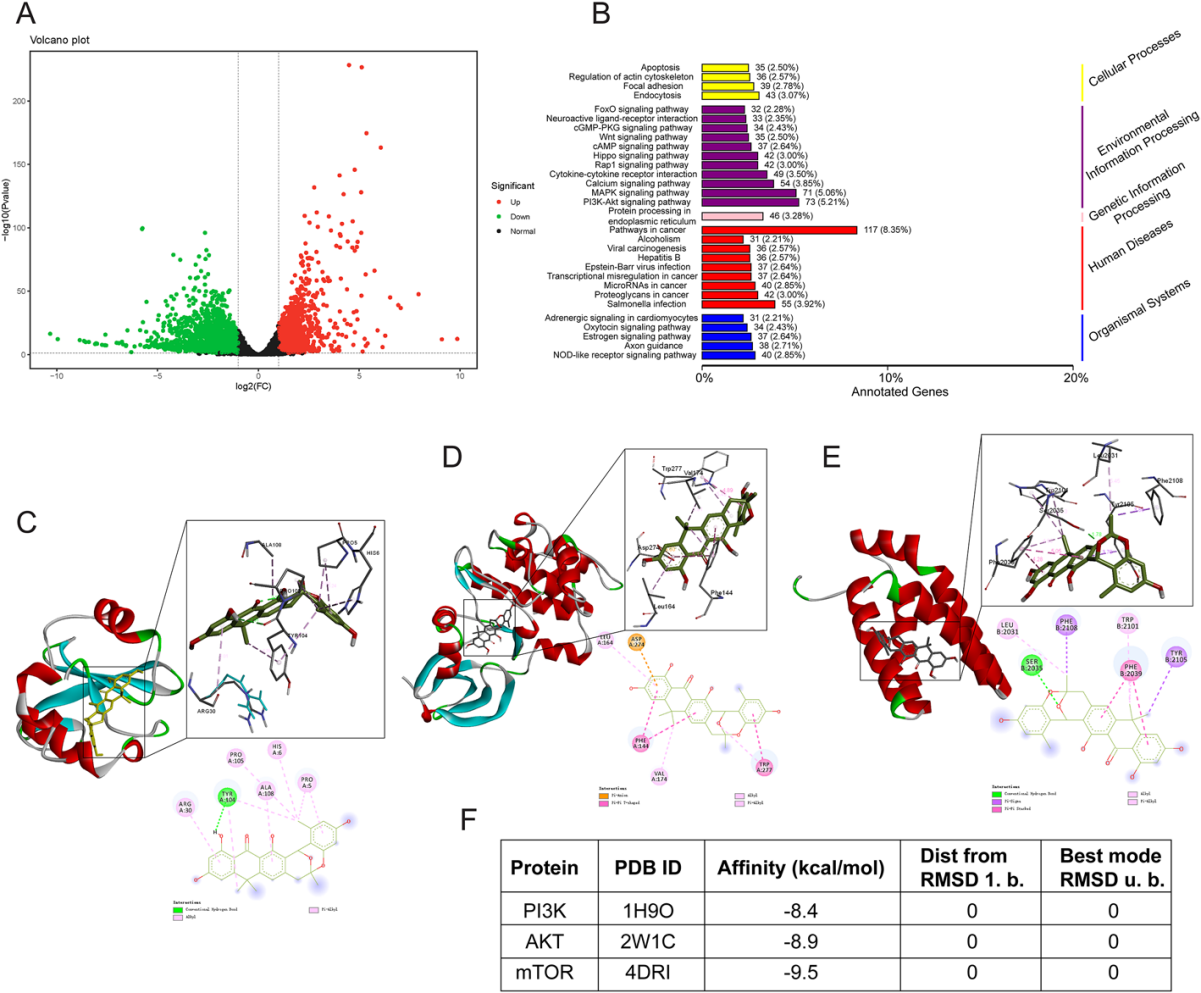
Transcriptomic analysis and molecular docking results. **A** Volcano plot depicting differentially expressed genes (**A** versus **C**). The cell line used for transcriptome analysis is H1299. **B** Kyoto Encyclopedia of Genes and Genomes (KEGG) pathway analysis (A versus C). **C** Molecular docking model of (+)-ABX with PI3K. **D** Molecular docking model of (+)-ABX with AKT. **E** Molecular docking model of (+)-ABX with mTOR. **F** Target protein information and docking efficiency scores

To further elucidate the molecular mechanisms underlying the inhibitory effects of (+)-ABX on NSCLC cells, transcriptomic analysis was conducted on cells from the control group (group A), the treatment group with 20 μM (+)-ABX (group B), and the treatment group with 40 μM (+)-ABX (group C). Principal component analysis (PCA) revealed significant differences in the transcriptomes among the three groups (Additional file 1: Fig. S7A). Pairwise comparisons of differentially expressed genes (DEGs) were performed between the groups (Fig. 5A and Additional file 1: Fig. S7B, C). The A versus B comparison identified 3086 DEGs, with 1006 upregulated and 2080 downregulated. The A versus C comparison yielded 3872 DEGs, consisting of 1526 upregulated and 2346 downregulated genes. The B versus C comparison exhibited 433 DEGs, including 289 upregulated and 144 downregulated genes. Venn diagrams (Additional file 1: Fig. S7D) depicted the unique and shared DEGs among the compared groups. Hierarchical clustering analysis was applied to the selected differentially expressed genes to group genes with similar expression patterns (Additional file 1: Fig. S7E). Annotation of the differentially expressed genes using KEGG categorized them according to pathway types (Fig. 5B and Additional file 1: Fig. S7F, G), including Wnt, cAMP, Hippo, Rap1, Calcium, MAPK, and the PI3K/AKT signaling pathway. Notably, the PI3K/AKT signaling pathway had the highest representation, and its close relationship with cell cycle arrest, apoptosis, and autophagy was evident. Consequently, it is inferred that (+)-ABX may exert its inhibitory effects on NSCLC cells through this pathway.

PI3K, AKT, and mTOR serve as pivotal targets within the PI3K/AKT/mTOR signaling pathway. To investigate the binding interactions between (+)-ABX and these aforementioned targets, molecular docking techniques were employed. The docking results provide an intuitive visualization of the overall three-dimensional structures of the three complexes. Enlarged views of the docking sites showcase the amino acid residues involved in ligand interactions, including bond lengths, while two-dimensional plots illustrate the types of forces at play (Fig. 5C–E). The binding free energies of (+)-ABX with PI3K, AKT, and mTOR were calculated as −8.4, −8.9, and −9.5 kcal/mol, respectively (Fig. 5F), underscoring the spontaneous and stable binding of (+)-ABX to these three targets.

### Induction of apoptosis and autophagy in NSCLC cells by (+)-ABX through the PI3K/AKT/mTOR signaling pathway

**Fig. 6:**
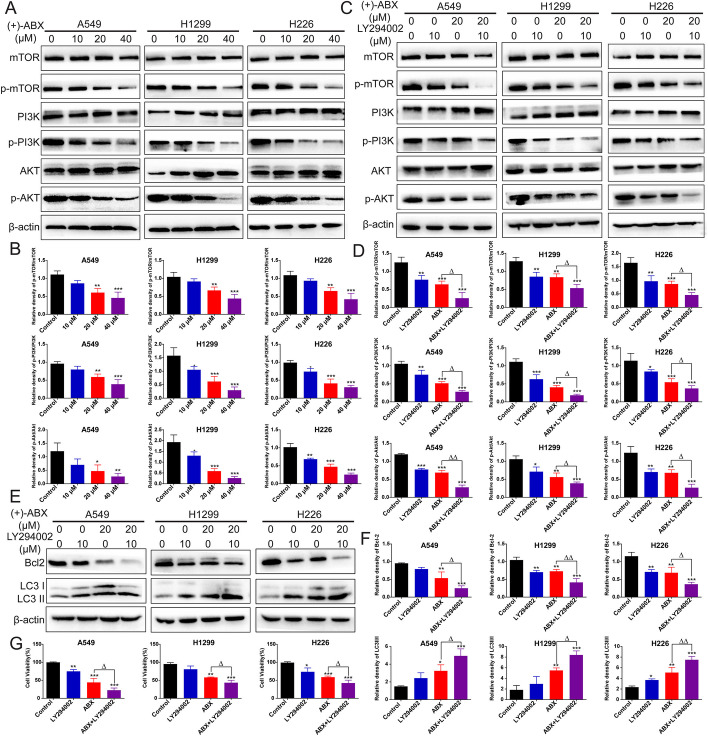
Inhibition of the PI3K/AKT/mTOR signaling pathway by (+)-ABX in NSCLC cells. **A** Impact of different concentrations of (+)-ABX treatment on the expression of PI3K/AKT/mTOR signaling pathway-related proteins in cells determined by western blotting. **B** Quantification of the data presented in (**A**). **C** Changes in the expression of PI3K/AKT/mTOR signaling pathway-related proteins upon intervention with LY294002 after treatment with (+)-ABX assessed by western blotting. **D** Quantification of the data presented in (**C**). **E** Alterations in the expression of apoptosis and autophagy-related proteins upon LY294002 intervention after treatment with (+)-ABX measured by western blotting. **F** Quantification of the data presented in (**E**). **G** Evaluation of the impact of LY294002 pretreatment on cell viability upon (+)-ABX treatment using the CCK-8 assay. Values are expressed as means ± SD. * or ^△^*P* < 0.05, ** or ^△△^*P* < 0.01, and ***P < 0.001, compared with the control. Data were collected from a minimum of three independent experiments

We validated the regulatory effect of (+)-ABX on the PI3K/AKT/mTOR signaling pathway in the three NSCLC cell lines at the protein expression level. The results demonstrated that as the concentration of (+)-ABX increased, the expression levels of p-PI3K/p-PI3K, p-AKT/p-AKT, and p-mTOR/p-mTOR progressively decreased in comparison to the control group. This suggests that (+)-ABX is capable of inhibiting the phosphorylation of PI3K, Akt, and mTOR in a dose-dependent manner, thereby suppressing this signaling pathway (Fig. 6A, B).

Furthermore, the impact of (+)-ABX on cell apoptosis and autophagy through modulation of the PI3K/AKT/mTOR signaling pathway was investigated using a PI3K inhibitor. The results revealed that in combination with LY294002, the expression of p-PI3K/p-PI3K, p-AKT/p-AKT, and p-mTOR/p-mTOR was further reduced compared with the use of (+)-ABX alone (Fig. 6C, D). Concurrently, when coadministered with LY294002, the expression of the antiapoptotic protein Bcl-2 was significantly diminished compared with the use of (+)-ABX alone, while the expression levels of the autophagic marker LC3-II/LC3-I were further elevated (Fig. 6E, F). Moreover, the cell viability was markedly decreased upon cotreatment (Fig. 6G). These findings confirm that (+)-ABX indeed induces apoptosis and autophagy in NSCLC cells through the PI3K/AKT/mTOR signaling pathway, ultimately leading to cell death. LY294002, as an inhibitor of PI3K, exhibited a synergistic effect with (+)-ABX. Furthermore, molecular docking techniques were employed to simulate the binding interactions of (+)-ABX and LY294002 with PI3K under identical conditions. As depicted in Additional file 1: Fig. S8, both compounds stably bound to distinct pockets on PI3K. This suggests potential interactions with different binding sites on PI3K, influencing its activity through distinct mechanisms. The collaborative impact of these two effects may lead to a more potent inhibitory outcome, manifesting as synergy in experimental results.

### (+)-ABX induces ROS accumulation in NSCLC cells further suppressing the PI3K/AKT/mTOR signaling pathway to inhibit NSCLC cell growth

**Fig. 7:**
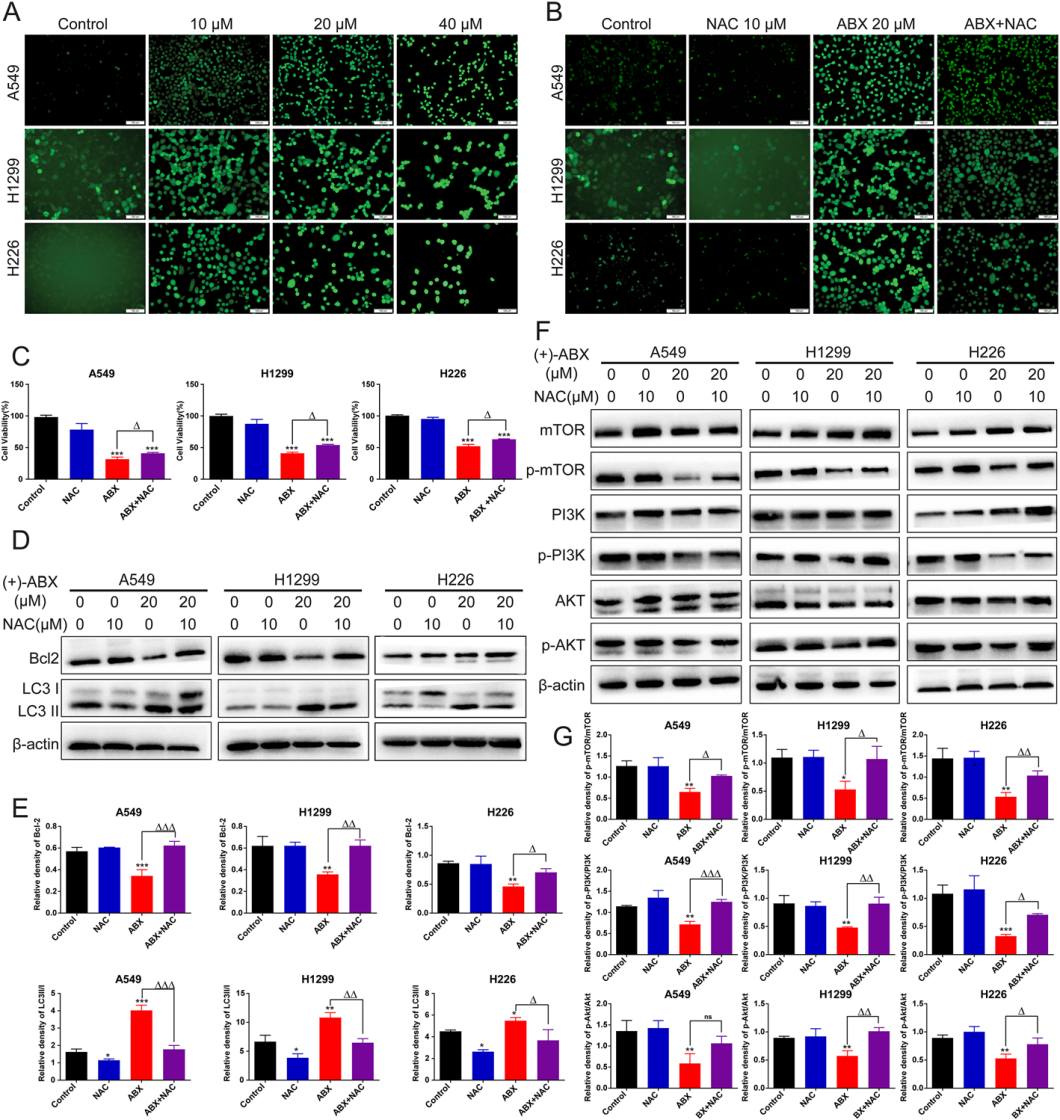
Enhanced ROS levels in NSCLC cells induced by (+)-ABX treatment. **A** Detection of ROS levels in cells treated with (+)-ABX using the DCFH-DA method. **B** Assessment of changes in ROS levels in cells treated with (+)-ABX after NAC intervention using the DCFH-DA method. **C** Impact of NAC intervention on cell viability after treatment with (+)-ABX measured by the CCK-8 assay. **D** Expression changes of apoptosis and autophagy-related proteins in cells treated with (+)-ABX after NAC intervention using western blotting. **E** Quantification of data from (**D**). **F** Expression changes of PI3K/AKT/mTOR pathway-related proteins in cells treated with (+)-ABX after NAC intervention using western blotting. **G** Quantification of data from (**F**). Data are presented as means ± SD. Statistical significance: * or ^△^*P* < 0.05, ** or ^△△^*P* < 0.01, and *** or ^△△△^*P* < 0.001 compared with the control. Data were obtained from at least three independent experiments

The preceding experiments revealed aberrant mitochondrial function in NSCLC cells treated with (+)-ABX, with mitochondria being the primary site of intracellular ROS generation. Utilizing ROS fluorescent probes, it was observed that (+)-ABX dose-dependently increased ROS production levels in all three types of NSCLC cells (Fig. 7A). Upon intervention with NAC, ROS generation levels were diminished (Fig. 7B), subsequently attenuating the inhibitory effect of (+)-ABX on these cells (Fig. 7C). These findings indicate that (+)-ABX can enhance ROS levels within these cells, and that ROS is involved in the inhibitory impact of (+)-ABX on NSCLC cells.

Disturbances in mitochondrial pathway-derived ROS levels are intricately linked with both cell apoptosis and autophagy. Upon NAC intervention in cells treated with (+)-ABX, there was an observed upregulation of the antiapoptotic protein Bcl-2 compared with when (+)-ABX was administered alone, while the expression ratio of the autophagic marker LC3-II/LC3-I was downregulated (Fig. 7D, E). These outcomes suggest that (+)-ABX can induce cell apoptosis and autophagy by elevating intracellular ROS levels.

Furthermore, the role of ROS in the process of (+)-ABX inhibiting the PI3K/AKT/mTOR pathway was investigated. Upon NAC intervention in cells treated with (+)-ABX, there was a significant reversal in the phosphorylation levels of the PI3K, AKT, and mTOR proteins compared with when (+)-ABX was administered alone (Fig. 7F, G). This suggests that ROS exerts a negative regulatory role on the PI3K/AKT/mTOR signaling pathway. In conclusion, (+)-ABX can induce ROS accumulation in NSCLC cells, further suppressing the PI3K/AKT/mTOR signaling pathway, enhancing levels of apoptosis and autophagy, and ultimately leading to cell death.

### (+)-ABX inhibits tumor growth in mice

**Fig. 8:**
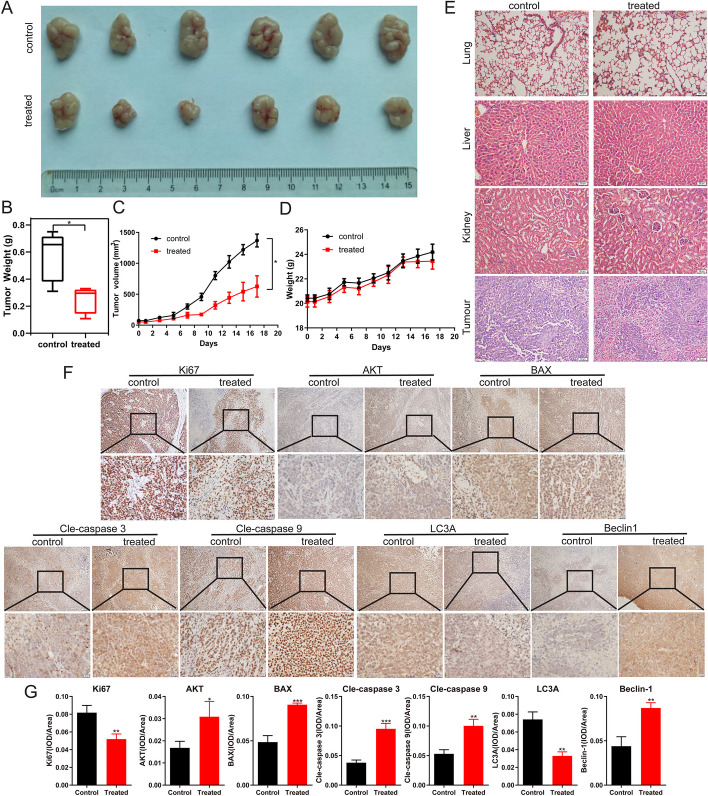
Impact of (+)-ABX on xenograft model in nude mice. **A** Ex vivo tumor imaging. **B** The tumor weight (*n* = 6). **C** Tumor growth curve plotted based on tumor volume measurements (*n* = 6). **D** Curve depicting changes in mouse body weight (*n* = 6). **E** Representative pathological sections of major organs and tumor tissues stained with H&E (*n* = 6). Scale bar, 50 μm. **F** IHC staining of Ki67, AKT, BAX, cleaved-caspase 3, cleaved-caspase 9, LC3A, and Beclin1 expression in tumor tissues. Scale bar, 100 and 50 μm. **G** Quantification of integrated optical density of IHC staining in (**E**). *n* = 3 (Additional file 1: Table S2). Data were presented as means ± SD. *P < 0.05, **P < 0.01, and ***P < 0.001 compared with the control. Information was collected from a minimum of three independent experiments

To evaluate the in vivo antitumor efficacy of (+)-ABX, we established a xenograft tumor model using A549 cells implanted subcutaneously in nude mice. Following a 21-day (+)-ABX treatment regimen, there were no significant differences in body weight between the two groups of nude mice (Fig. 8D). Daily observations of their food and water intake, mental status, stool consistency, and other parameters revealed no anomalies, ruling out adverse effects of (+)-ABX on the overall health of the mice. In comparison with the control group, the treatment group exhibited a reduction in tumor mass (Fig. 8A, B) and a decelerated tumor volume growth rate (Fig. 8C). The average tumor volume in the untreated group measured 1367.5 ± 104.7 mm^3^, whereas in the treatment group, it was 625.7 ± 172.9 mm^3^, demonstrating a significant difference (*P* < 0.05) and an inhibition rate of 54.24%. These findings strongly indicate that (+)-ABX effectively suppresses tumor growth in vivo.

Furthermore, H&E staining analysis was conducted on the major organs and tumor masses of both groups of mice (Fig. 8E). The results revealed that (+)-ABX treatment at a dose of 2 mg/kg did not induce significant toxicity in the major organs. In the control group, tumor cells exhibited a tight and irregular arrangement, with larger cell volumes and variable shapes including round, spindle, and polygonal forms. There was a substantial quantity of cells, enriched cytoplasm, evident megakaryocytic cells, pronounced cellular heterogeneity, frequent nuclear division, and slight focal necrosis in certain areas. In the treatment group, the number of cells was relatively reduced, with fewer instances of nuclear division. Clear foci of tumor cell necrosis were observed in localized areas, where necrotic cells displayed shrinkage, dissolution, or fragmentation, resulting in larger necrotic zones. Generally, under conditions without drug interference, tumor necrosis tends to intensify with increasing volume. The tumors in the treatment group exhibited smaller volumes compared with the control group, yet more severe necrosis, indicating that (+)-ABX treatment accelerated tumor necrosis. This pathological evidence corroborates the anticancer effect of (+)-ABX.

Finally, the mechanisms underlying the in vivo anticancer effects of (+)-ABX were corroborated using IHC techniques. The results demonstrated a significant reduction in the expression of cell proliferation-associated antigen Ki67 and platelet-endothelial cell adhesion molecule CD31 in tumor tissues of the treatment group (Fig. 8F, G). Conversely, the expression of proapoptotic indicators Bax, cleaved-caspase 3, and cleaved-caspase 9 was notably increased, while the expression of autophagy-related indicators Beclin-1 was elevated and LC3A was diminished. Additionally, the expression of AKT was elevated, suggesting potential inhibition of AKT phosphorylation. In summary, these findings collectively suggest that (+)-ABX can suppress the AKT pathway in tumor tissues in vivo, inducing tumor apoptosis and autophagy, while inhibiting tumor proliferation.

## Discussion

ABXs represent a class of structurally novel oxygenated anthrabenzoxocinone compounds. They feature a distinctive pair of chiral oxygen-bridged scaffolds along with a 1,3-dihydroxy-10,10-dimethylanthrone (DHDA) unit, which exhibits a high affinity for binding the condensation enzyme FabF of bacterial fatty acid synthases [33]. The robust antibacterial activity and unique mechanism of action of these compounds have drawn the attention of both chemists and synthetic biologists. Currently, 24 naturally isolated and synthetically prepared ABXs compounds exist, with their investigation primarily focused on antibacterial potential [34–36]. In this study, the in vitro and in vivo anticancer activity of (+)-ABX is unveiled for the first time, and an exploration of its plausible mechanisms of action is conducted. This research lays the groundwork for the potential application of (+)-ABXs compounds in cancer therapy.

The cell cycle checkpoint is a vital regulatory mechanism in eukaryotic cells, ensuring the integrity of chromosome numbers and the proper progression of the cell cycle [37]. Despite having intact checkpoint signaling pathways, a shared feature among cancer cells is the deficiency in G1 checkpoint control. Cancer cells primarily rely on the S and G2 checkpoints to prevent DNA damage from transforming into cell death. Prolonged arrest of tumor cells in the S and G2/M phases not only inhibits tumor growth but also accumulates DNA damage, triggering apoptosis. Therefore, arresting cells in S and G2/M phases plays a pivotal role in combating tumors [38]. Currently, many clinically used chemotherapeutic agents target the S or G2/M phases, such as cytarabine, paclitaxel, and bleomycin [39–41]. Our research supports that (+)-ABX significantly induces cell cycle arrest in both the S and G2/M phases of NSCLC cells. The mechanism behind the G2/M arrest involves upregulating the expression of the cell cycle inhibitor protein p21, leading to the suppression of cyclin B and CDK1 expression.

Cell apoptosis is one of the intrinsic surveillance and balancing mechanisms of the cell cycle, capable of promptly eliminating harmful and aberrant cells. Inducing apoptosis in tumor cells is regarded as a significant approach for tumor prevention and treatment [42]. The occurrence of cell apoptosis is primarily regulated by the mitochondrial pathway, death receptor pathway, and endoplasmic reticulum stress pathway [43]. When cells are exposed to internal apoptotic stimuli, the mitochondrial apoptotic pathway can be activated. Notably, the antiapoptotic protein Bcl-2 and proapoptotic protein BAX act as upstream regulators of the mitochondrial pathway, exerting a crucial role in modulating mitochondrial membrane permeability during the apoptotic signal transduction process. This modulation further regulates the activation of caspase-9, a downstream protein of the mitochondrial pathway. Activated caspase-9 can subsequently activate caspase-3, which is the pivotal execution enzyme in mitochondrial-mediated cell apoptosis [31]. This study demonstrates that (+)-ABX significantly enhances the apoptosis rate of NSCLC cells. Its mechanism of action involves the modulation of the expression levels of the Bcl-2 protein family through the mitochondrial pathway, subsequently triggering a cascade of caspase reactions and ultimately inducing apoptosis in NSCLC cells.

Autophagy exerts opposing dual roles in tumor cells, maintaining survival while also promoting cell death, and these roles can interconvert under certain circumstances. Presently, the process of autophagy has become a pivotal target in cancer therapy, with both enhancing and inhibiting autophagy considered effective treatment strategies [44]. Autophagy and apoptosis individually regulate the turnover of intracellular organelles and proteins, as well as the cellular turnover within organisms. Many stress pathways sequentially trigger autophagy and apoptosis within the same cell. Generally, autophagy can impede the induction of cell apoptosis, whereas apoptosis-related caspase activation can shut down the autophagic process. However, in specific situations, autophagy or autophagy-related proteins might contribute to inducing cell apoptosis or necrosis, as excessive autophagy degradation of cytoplasm leads to “autophagic cell death” [45]. Our research indicates that (+)-ABX induces macroautophagy in NSCLC cells by regulating the expression of autophagy-related proteins. While autophagy activation may be one of the mechanisms through which (+)-ABX inhibits NSCLC, the combination of autophagy inhibitor CQ seemingly enhances the inhibitory effect of (+)-ABX on NSCLC by promoting apoptosis. This suggests that combining (+)-ABX with an autophagy inhibitor could potentially offer an improved therapeutic strategy.

In NSCLC, the abnormal activation of the PI3K/AKT/mTOR signaling pathway is frequently detected. A spectrum of cancer-related functions is linked with this signaling pathway, encompassing tumor cell proliferation, migration, regulation of the cell cycle, adhesion, apoptosis, autophagy, and angiogenesis within tumor tissues. Indeed, the escalation of pathway activation correlates with the progression of malignancy, and the potential to hinder its operation holds promise in thwarting the advancement of lung cancer [46, 47]. In our study, compelling evidence is presented, demonstrating that the PI3K/AKT/mTOR signaling pathway in NSCLC cells is significantly inhibited by (+)-ABX. The inhibitory effects of (+)-ABX on this pathway are further intensified by the PI3K inhibitor LY294002, facilitating apoptosis and autophagy incited by (+)-ABX, consequently enhancing its capacity for anticancer activity. Moreover, the outcomes of molecular docking suggest that (+)-ABX can establish stable bindings with the PI3K, AKT, and mTOR proteins. These findings underscore the pronounced significance of the PI3K/AKT/mTOR signaling pathway as a pivotal target for the inhibition of NSCLC growth by (+)-ABX.

ROS serves as a secondary messenger in the PI3K/AKT/mTOR signaling pathway, playing a crucial role, potentially regulating cell survival and death by modulating apoptosis and autophagy pathways [48, 49]. The pharmacological induction of ROS is increasingly recognized as a principle for selectively targeting tumor cells, and the combination of PI3K inhibitors with agents inducing ROS has shown synergistic antitumor effects [50]. Our study demonstrates that (+)-ABX can induce oxidative stress in NSCLC cells, leading to an increase in intracellular ROS levels. This, in turn, enhances the inhibitory effects on the PI3K/AKT/mTOR signaling pathway, promoting apoptosis and autophagy. The attenuation of these effects by the ROS inhibitor NAC suggests that triggering oxidative stress in NSCLC cells is one of the mechanisms underlying the anticancer effects of (+)-ABX.

Tumors often rapidly develop resistance to drugs by activating compensatory mechanisms that circumvent the inhibited signaling pathways [51]. Due to the activation of compensatory signaling pathways, many promising single-pathway targeted drugs have yielded disappointing clinical outcomes [52, 53]. In contrast, the development of combination therapies and multipathway targeted drugs holds more promise for cancer treatment [54]. Interestingly, transcriptomic results indicate that the antitumor-related signaling pathways affected by (+)-ABX appear to be diverse, involving pathways such as MAPK, calcium, Rap1, and Hippo. How (+)-ABX modulates these signaling pathways to exert its anticancer effects warrants further in-depth exploration.

## Conclusions

**Fig. 9:**
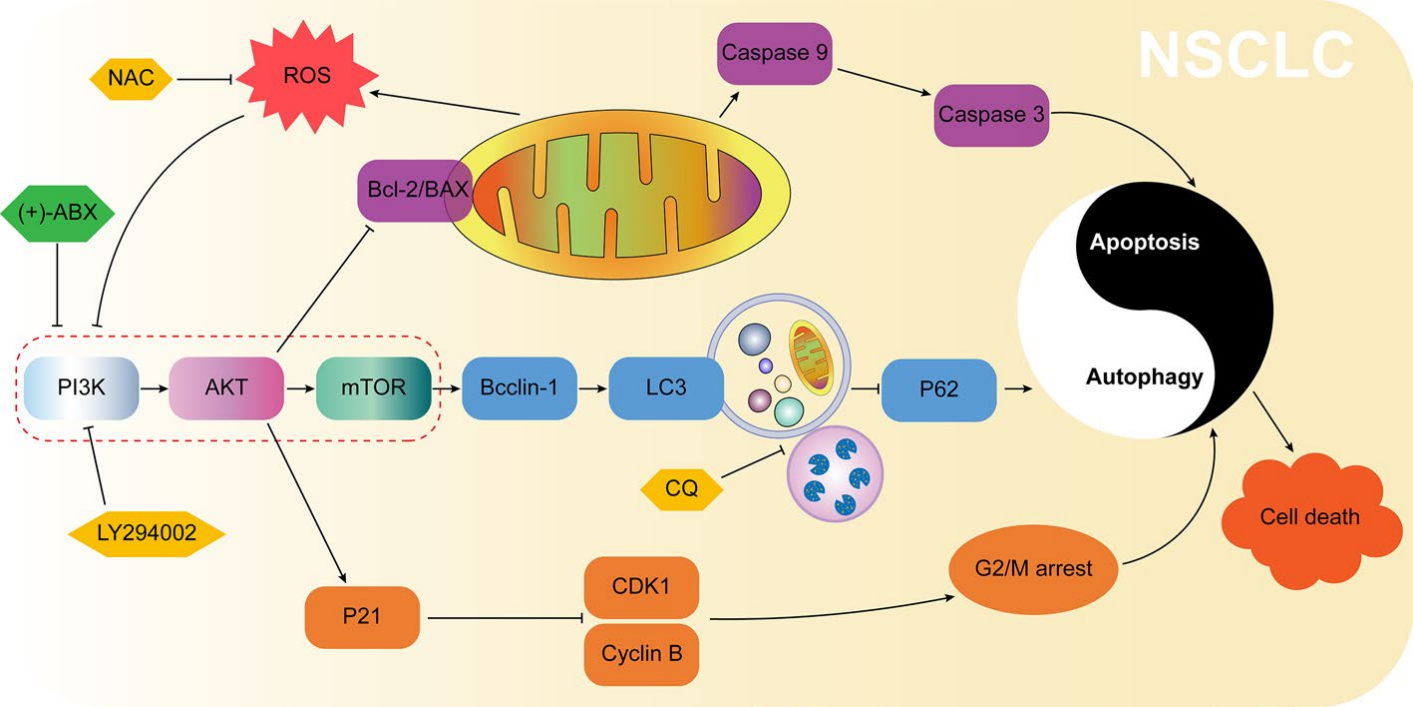
Molecular mechanism underlying the anti-NSCLC activity of (+)-ABX inferred from the findings of this study

(+)-ABX effectively inhibits the proliferation, migration, and invasion of NSCLC cells. Its mechanism of action potentially involves targeted inhibition of the PI3K/AKT/mTOR signaling pathway, thereby regulating G2/M phase arrest, apoptosis, and autophagy in NSCLC cells. Additionally, it elevates ROS levels to further suppress this signaling pathway, ultimately leading to NSCLC cell death (Fig. 9). In vivo experiments further validate the therapeutic efficacy of (+)-ABX. In summary, our research outcomes provide a promising lead compound for the treatment of NSCLC.

### Supplementary Information

**Additional file 1.** Includes the primer sequences in qRT-PCR assay; effects of different concentrations of (+)-ABX on the colony formation of three NSCLC cell lines; effects of different concentrations of (+)-ABX on migration of three NSCLC cell lines; effects of different concentrations of (+)-ABX on invasion of three NSCLC cell lines; effects of different concentrations of (+)-ABX on the cell cycle distribution of three NSCLC cell lines; flow cytometry analysis of apoptosis rates in three NSCLC cell lines treated with different concentrations of (+)-ABX; transcriptomic analysis; the images of immunohistochemistry staining in three repetitions; and the images of the original western blots in three repetitions.** Fig. S1**. Antitumor activity screening results of (+)-ABX. The screening range includes nasopharyngeal carcinoma cells (CNE1), colorectal cancer cells (LOVO), thyroid cancer cells (TPC-1), melanoma cells (B16), liver cancer cells (MHCC97H, Hep1), and lung cancer cells (A549, H1299, and H226). The IC_50_ values were determined using the CCK-8 method. **Table S1**. The primer sequences in qRT-PCR assay. **Fig. S2**. Effects of different concentrations of (+)-ABX on the colony formation of three NSCLC cell lines. **Fig. S3**. Effects of different concentrations of (+)-ABX on migration of three NSCLC cell lines (magnification ×200; scale bar, 50 μm). **Fig. S4**. Effects of different concentrations of (+)-ABX on invasion of three NSCLC cell lines (magnification × 200; scale bar, 50 μm). **Fig. S5.** Effects of different concentrations of (+)-ABX on the cell cycle distribution of three NSCLC cell lines. **Fig. S6.** Flow cytometry analysis of apoptosis rates in three NSCLC cell lines treated with different concentrations of (+)-ABX. **Fig. S7.** Transcriptomic analysis. Group A: untreated group; group B: 20 μM (+)-ABX treated group; group C: 40 μM (+)-ABX treated group. **A** Principal component analysis (PCA). **B** Volcano plot of differentially expressed genes (**A** versus **B**). **C** Volcano plot of differentially expressed genes (**B** vs **C**). **D** Venn diagram of differentially expressed genes. **E** Hierarchical clustering heatmap of differentially expressed genes in three groups. **F** Kyoto Encyclopedia of Genes and Genomes (KEGG) pathway analysis (**A** vs **B**). **G** Kyoto Encyclopedia of Genes and Genomes (KEGG) pathway analysis (**B** versus **C**). **Fig. S8.** Differences in the binding sites between (+)-ABX and LY294002 with the PI3K protein. (A) Docking results of (+)-ABX with PI3K. (B) Docking results of LY294002 with PI3K. **Table S2**. The images of immunohistochemistry staining in three repetitions. **Table S3**. The images of the original western blots in three repetitions.

## Data Availability

All data generated or analyzed during this study are included in this article (and its additional files).

## Abbreviations

(+)-ABX: (+)-Anthrabenzoxocinone
NSCLC: Non-small cell lung cancer
ROS: Reactive oxygen species
CQ: Chloroquine
NAC: N-acetyl-L-cysteine
FBS: Fetal bovine serum
CCK-8: Cell counting kit-8
PBS: Phosphate-buffered saline
PI: Propidium iodide
MMP: Measurement of mitochondrial membrane potential
PCR: Polymerase chain reaction
TEM: Transmission electron microscopy
KEGG: Kyoto encyclopedia of genes and genomes
H&E: Hematoxylin and eosin
IHC: Immunohistochemical staining
DEGs: Differentially expressed genes
