# Supplementary material for: Targeted inhibition of the PI3K/AKT/mTOR pathway by (+)-anthrabenzoxocinone induces cell cycle arrest, apoptosis, and autophagy in non-small cell lung cancer
Source: Cell Mol Biol Lett. 2024 Apr 23;29:58. doi: 10.1186/s11658-024-00578-6 (PMC11036658; doi:10.1186/s11658-024-00578-6)
Supplement: Supplementary file 1 — Additional file 1. Includes the primer sequences in qRT-PCR assay; effects of different concentrations of (+)-ABX on the colony formation of three NSCLC cell lines; effects of different concentrations of (+)-ABX on migration of three NSCLC cell lines; effects of different concentrations of (+)-ABX on invasion of three NSCLC cell lines; effects of different concentrations of (+)-ABX on the cell cycle distribution of three NSCLC cell lines; flow cytometry analysis of apoptosis rates in three NSCLC cell lines treated with different concentrations of (+)-ABX; transcriptomic analysis; the images of immunohistochemistry staining in three repetitions; and the images of the original western blots in three repetitions. Fig. S1. Antitumor activity screening results of (+)-ABX. The screening range includes nasopharyngeal carcinoma cells (CNE1), colorectal cancer cells (LOVO), thyroid cancer cells (TPC-1), melanoma cells (B16), liver cancer cells (MHCC97H, Hep1), and lung cancer cells (A549, H1299, and H226). The IC50 values were determined using the CCK-8 method. Table S1. The primer sequences in qRT-PCR assay. Fig. S2. Effects of different concentrations of (+)-ABX on the colony formation of three NSCLC cell lines. Fig. S3. Effects of different concentrations of (+)-ABX on migration of three NSCLC cell lines (magnification ×200; scale bar, 50 μm). Fig. S4. Effects of different concentrations of (+)-ABX on invasion of three NSCLC cell lines (magnification × 200; scale bar, 50 μm). Fig. S5. Effects of different concentrations of (+)-ABX on the cell cycle distribution of three NSCLC cell lines. Fig. S6. Flow cytometry analysis of apoptosis rates in three NSCLC cell lines treated with different concentrations of (+)-ABX. Fig. S7. Transcriptomic analysis. Group A: untreated group; group B: 20 μM (+)-ABX treated group; group C: 40 μM (+)-ABX treated group. A Principal component analysis (PCA). B Volcano plot of differentially expressed genes (A versus B). C Volcano plot of differen [file 11658_2024_578_MOESM1_ESM.pdf]

Additional file 1

Targeted Inhibition of the PI3K/AKT/mTOR Pathway by (+)-Anthrabenzoxocinone Induces Cell Cycle Arrest, Apoptosis, and Autophagy in Non-Small Cell Lung Cancer

Xiao-Qian Li [a], Xiao-Ju Cheng [a], Jie Wu [a], Kai-Feng Wu [a]*, Tie Liu [a,b]*

[a]*The Third Affiliated Hospital of Zunyi Medical University (The First People's Hospital of Zunyi), Scientific Research Center, Guizhou 563002, People's Republic of China*

[b]*Yunnan Characteristic Plant Extraction Laboratory, Key Laboratory of Medicinal Chemistry for Natural Resource, Key Laboratory of Medicinal Chemistry for Natural Resource, Ministry of Education and Yunnan Province, School of Chemical Science and Technology, Yunnan University, Kunming, 650091, People's Republic of China*

***Corresponding Author:**

Tie Liu and Kai-Feng Wu

Tel: +86-851-23110859

Email: liutiefes@outlook.com (Tie Liu), kiphoonwu@126.com (Kai-Feng Wu)

Content of Supplementary Data

**Fig. S1.** Antitumor activity screening results of (+)-ABX.	**3**
**Table S1.** The primer sequences in qRT-PCR assay	**4**
**Fig. S2.** Effects of different concentrations of (+)-ABX on the colony formation of three NSCLC cell lines	**5**
**Fig. S3.** Effects of different concentrations of (+)-ABX on migration of three NSCLC cell lines	**6**
**Fig. S4.** Effects of different concentrations of (+)-ABX on invasion of three NSCLC cell lines	**7**
**Fig. S5.** Effects of different concentrations of (+)-ABX on the cell cycle distribution of three NSCLC cell lines	**8**
**Fig. S6.** Flow cytometry analysis of apoptosis rates in three NSCLC cell lines treated with different concentrations of (+)-ABX	**9**
**Fig. S7.** Transcriptomic analysis.	**10**
**Fig. S8.** Differences in the binding sites between (+)-ABX and LY294002 with the PI3K protein.	**11**
**Table S2.** The images of immunohistochemistry staining in three repetitions	**12**
**Table S3.** The images of the original western blots in three repetitions	**17**
The original images of **Fig. 7A** and **7B**.	**59**

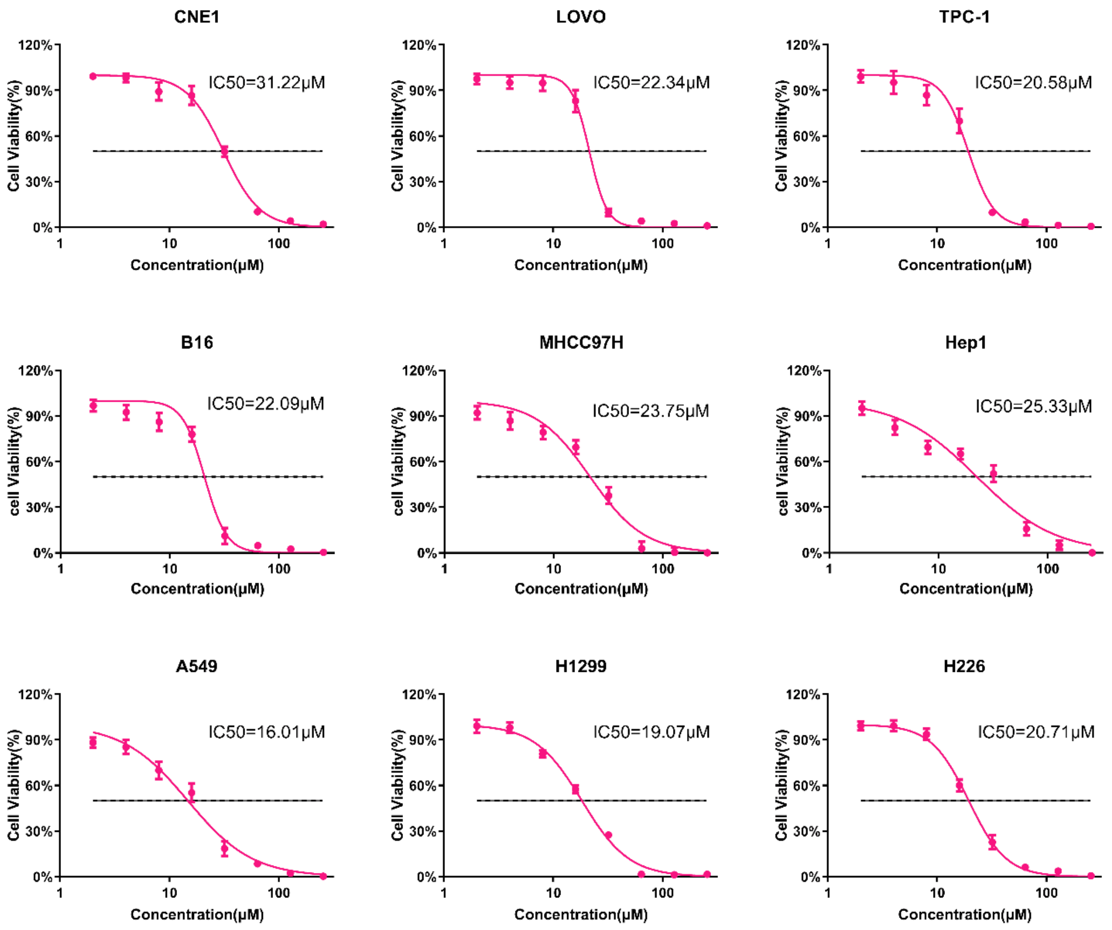

Fig. S1. Antitumor activity screening results of (+)-ABX. The screening range includes nasopharyngeal carcinoma cells (CNE1), colorectal cancer cells (LOVO), thyroid cancer cells (TPC-1), melanoma cells (B16), liver cancer cells (MHCC97H, Hep1), and lung cancer cells (A549, H1299, and H226). The IC50 values were determined using the CCK-8 method.

Table S1. The primer sequences in qRT-PCR assay.

Gene	Primer name	Sequence (5’ to 3’)
Bcl2	Bcl2-F	GGAGGATTATGGCCTTCTTTG
	Bcl2-R	GCAGGCATGTTGACTTCACTTG
BAX	BAX -F	TTTGCTTCAGGGTTTCATCC
	BAX -R	GCCACAAAGATGGTCACGGT
Caspase-9	Caspase-9-F	GTGGTGGGGAGCAGAAAGAC
	Caspase-9-R	TGCAAGATAAGGCAGGGTGAG
β-actin	β-actin -F	AGACCTTCAACACCCCAG
	β-actin -R	CACGATTTCCCTCTCAGC

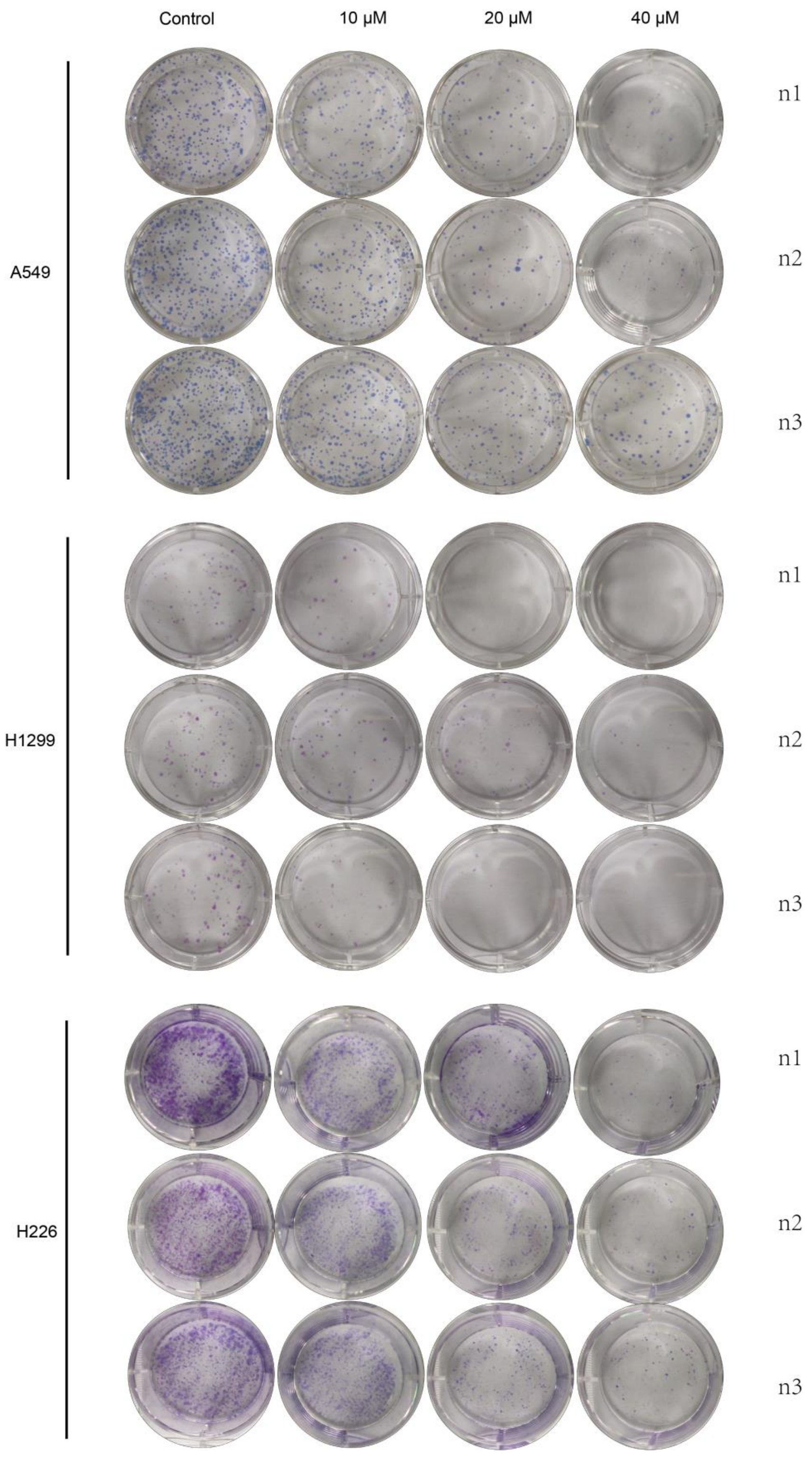

Fig. S2. Effects of different concentrations of (+)-ABX on the colony formation of three NSCLC cell lines.

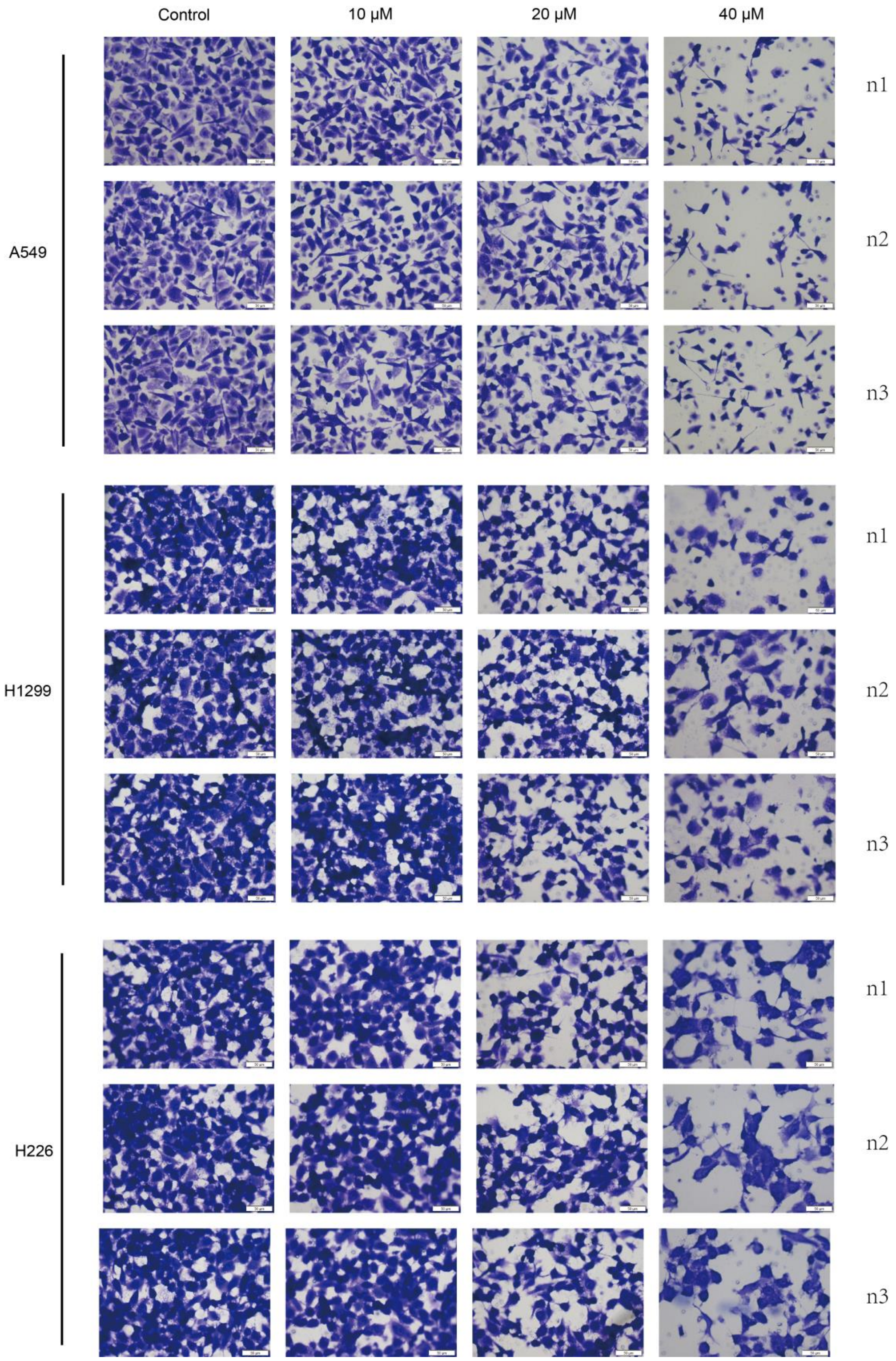

Fig. S3. Effects of different concentrations of (+)-ABX on migration of three NSCLC cell lines (magnification ×200, scale bar = 50 μm).

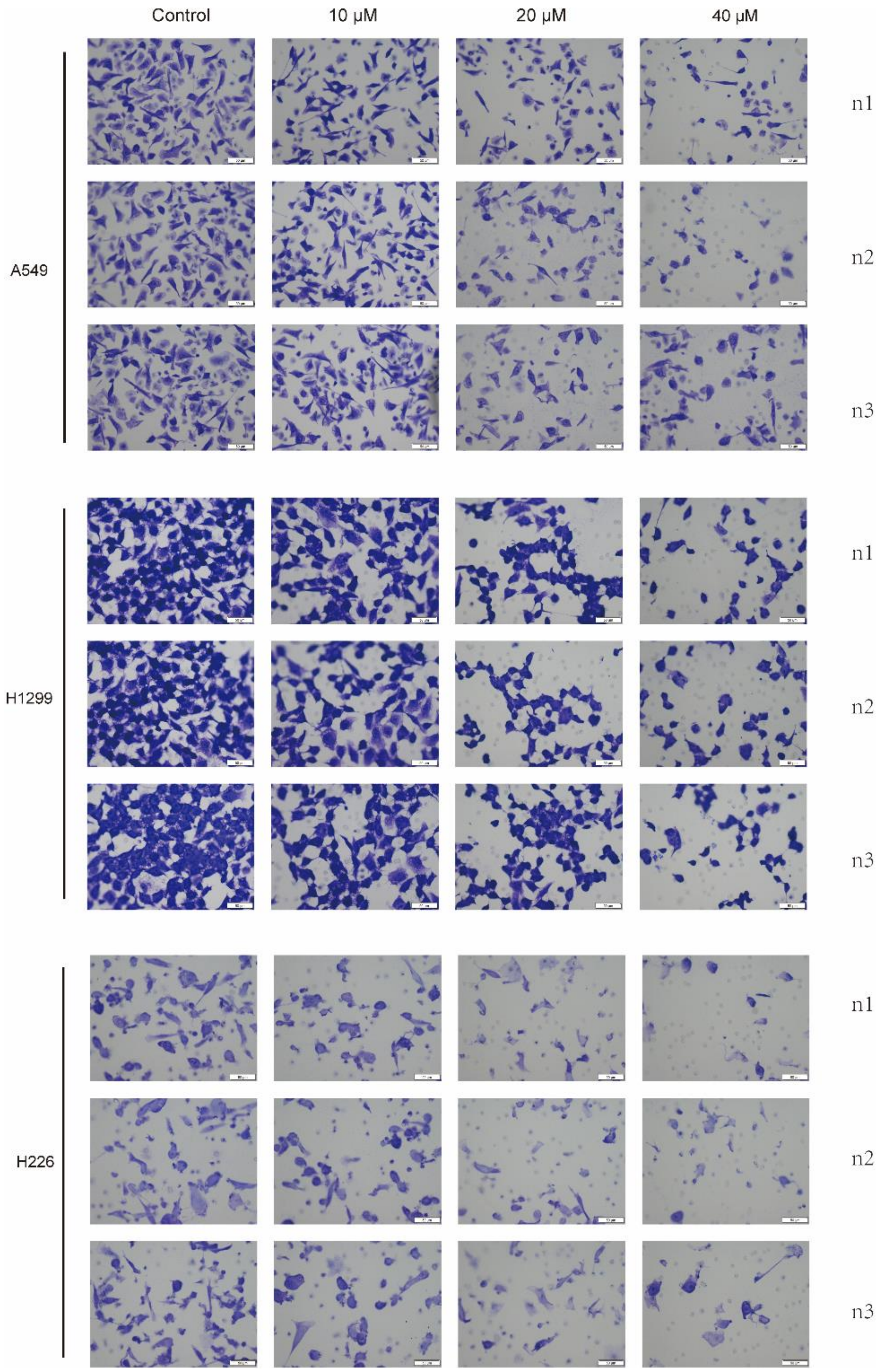

Fig. S4. Effects of different concentrations of (+)-ABX on invasion of three NSCLC cell lines (magnification ×200, scale bar = 50 μm).

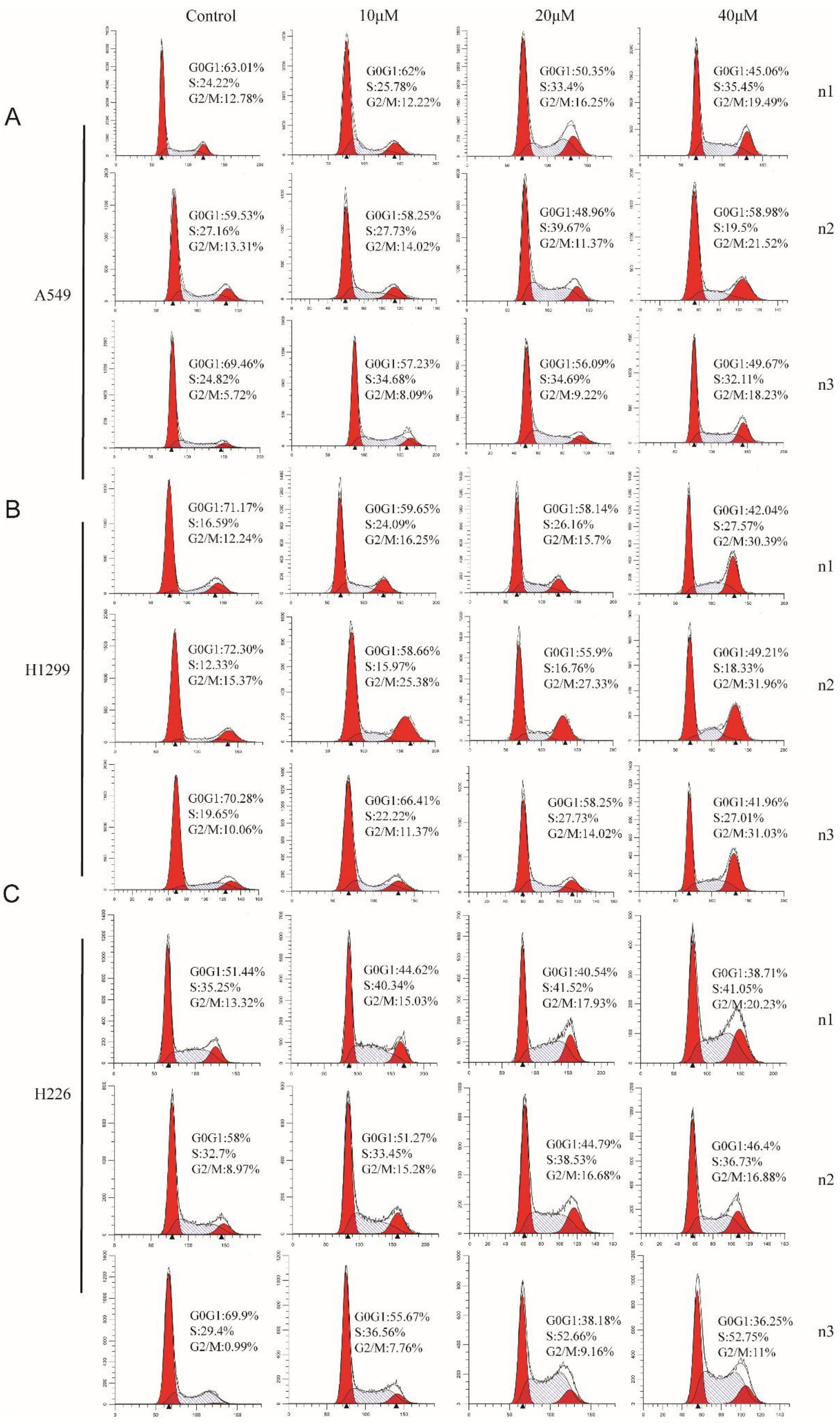

Fig. S5. Effects of different concentrations of (+)-ABX on the cell cycle distribution of three NSCLC cell lines.

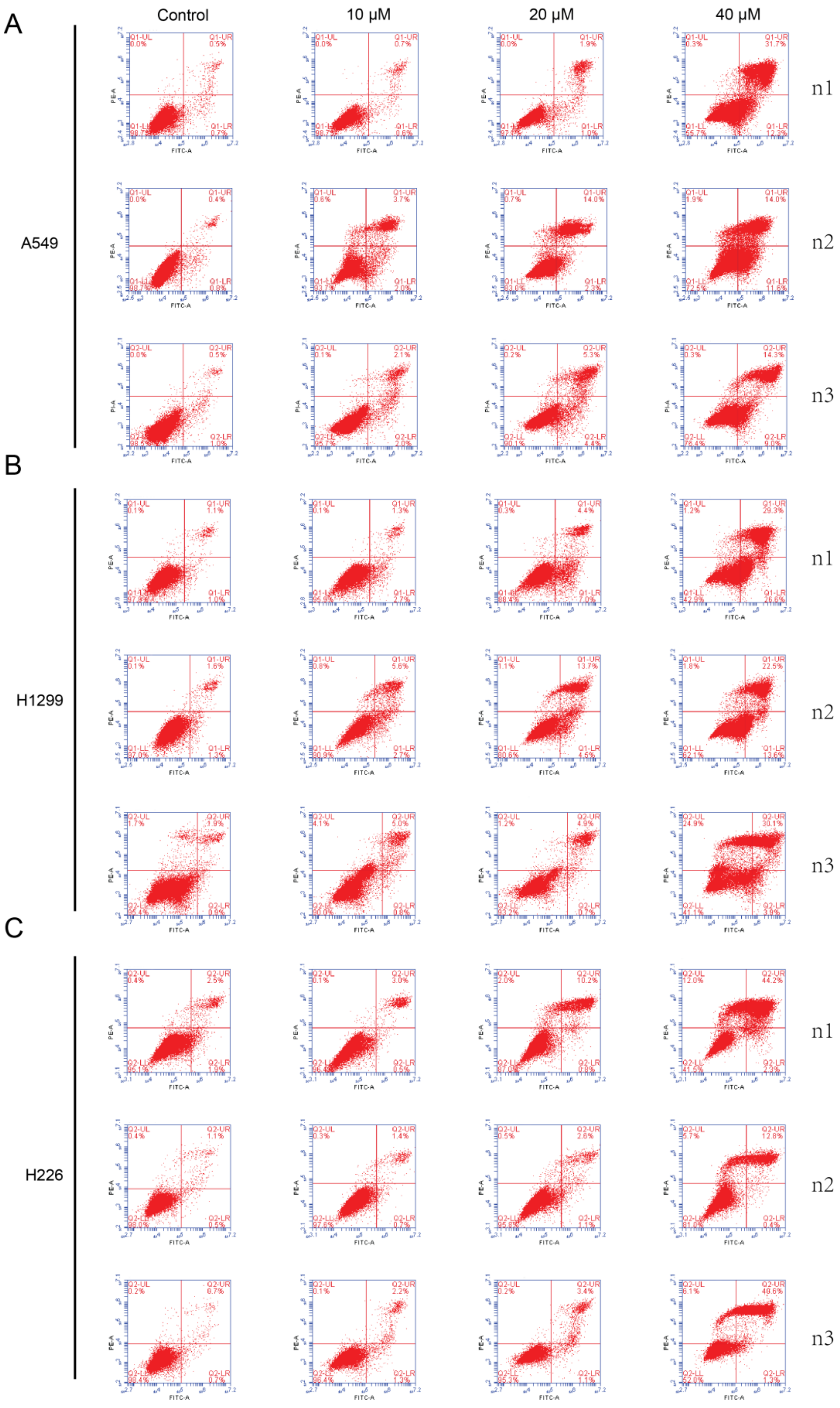

Fig. S6. Flow cytometry analysis of apoptosis rates in three NSCLC cell lines treated with different concentrations of (+)-ABX.

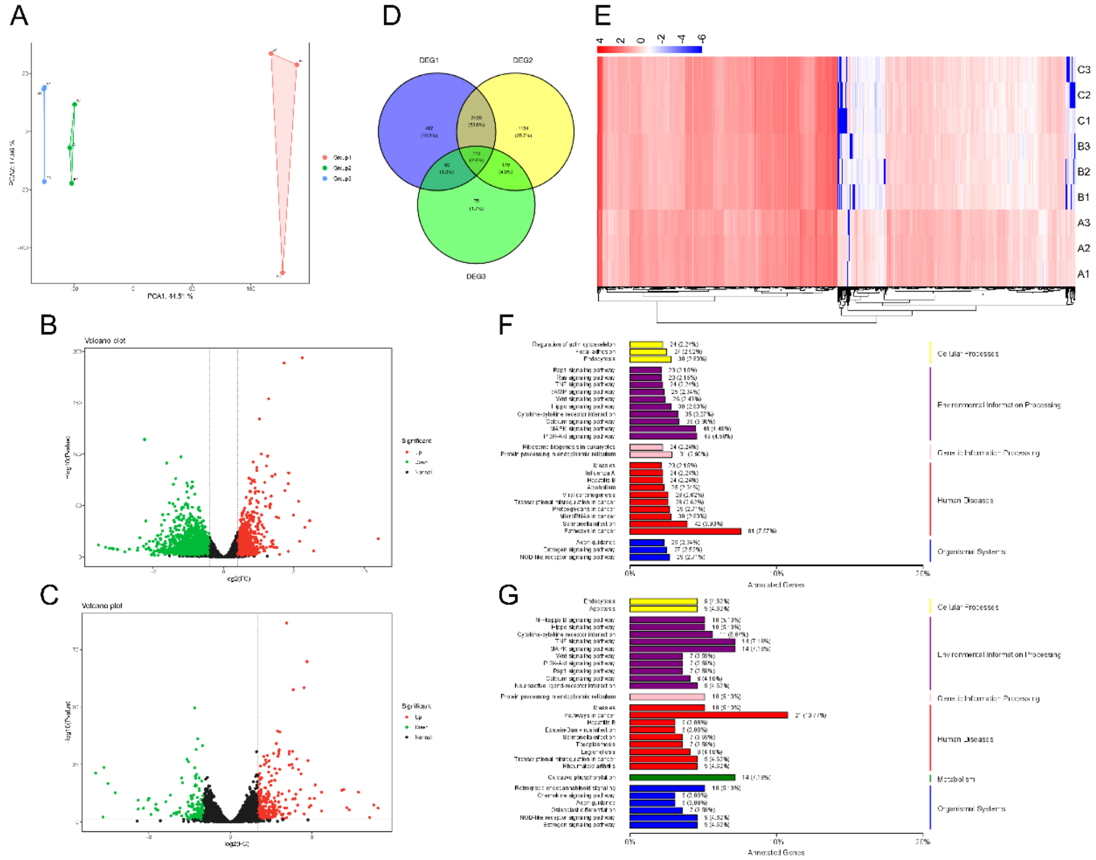

Fig. S7. Transcriptomic analysis. Group A: untreated group ; Group B: 20 μM (+)-ABX treated group ; Group C: 40 μM (+)-ABX treated group. (A) Principal Component Analysis (PCA). (B) Volcano plot of differentially expressed genes (A vs B). (C) Volcano plot of differentially expressed genes (B vs C). (D) Venn diagram of differentially expressed genes. (E) Hierarchical clustering heatmap of differentially expressed genes in three groups. (F) Kyoto Encyclopedia of Genes and Genomes (KEGG) pathway analysis (A vs B). (G) Kyoto Encyclopedia of Genes and Genomes (KEGG) pathway analysis (B vs C).

A

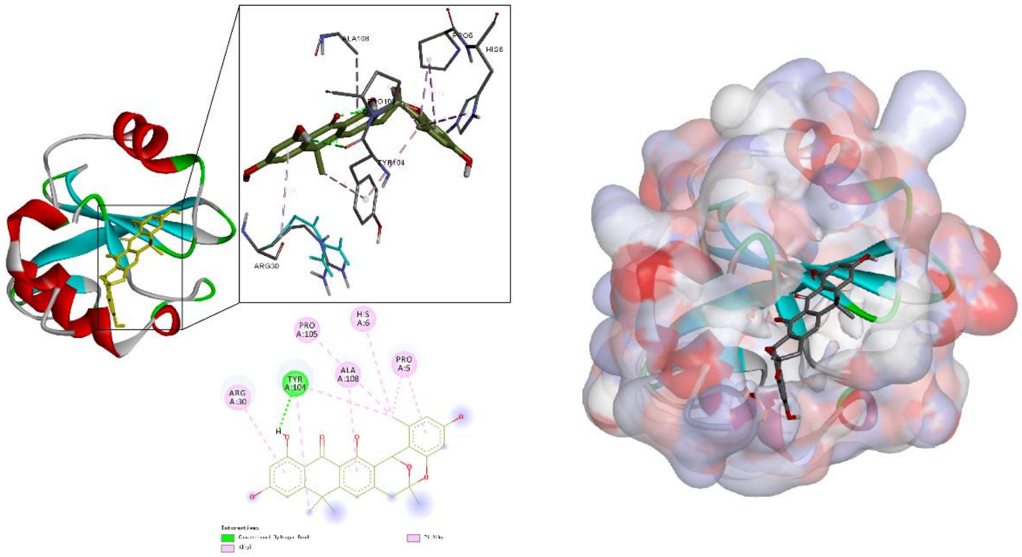

Ligand	Protein	PDB ID	Affinity (kcal/mol)	Dist from RMSD l. b.	Best mode RMSD u. b.
(+)-ABX	PI3K	1H9O	-8.4	0	0

B

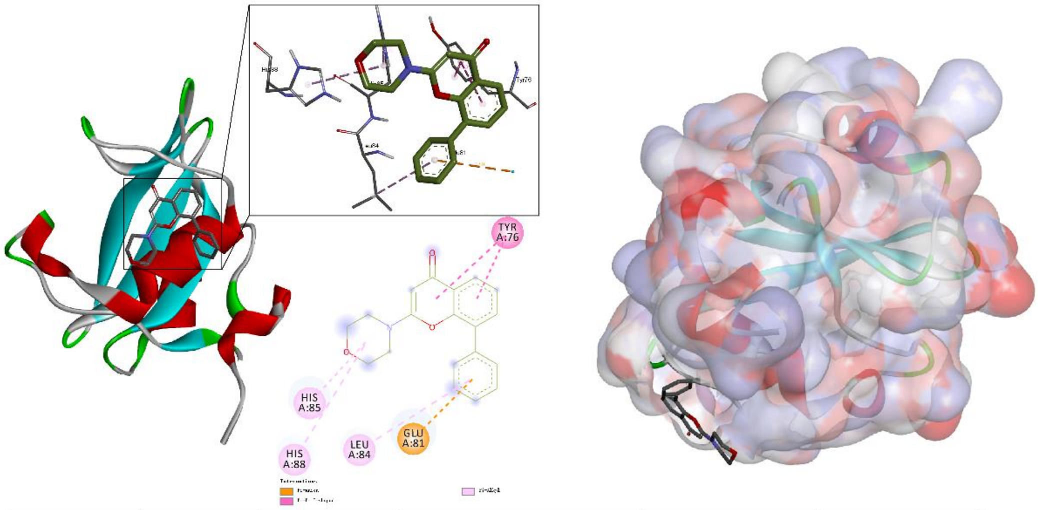

Ligand	Protein	PDB ID	Affinity (kcal/mol)	Dist from RMSD l. b.	Best mode RMSD u. b.
LY294002	PI3K	1H9O	-6.7	0	0

Fig. S8. Differences in the binding sites between (+)-ABX and LY294002 with the PI3K protein. (A) Docking results of (+)-ABX with PI3K. (B) Docking results of LY294002 with PI3K.

Table S2. The images of immunohistochemistry staining in three repetitions

Beclin1	n1	n2	n3
Control ×100 (100μm)	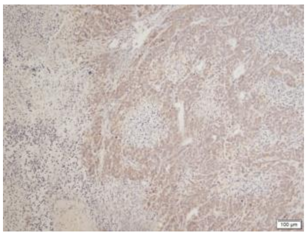	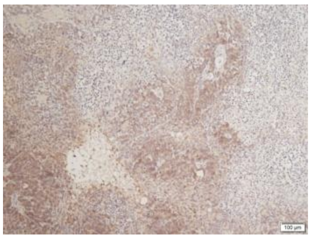	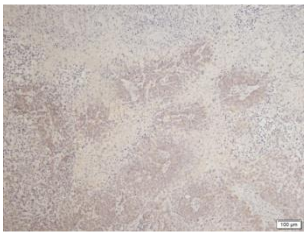
Control ×400 (20μm)	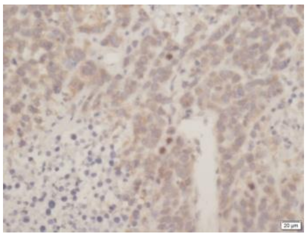	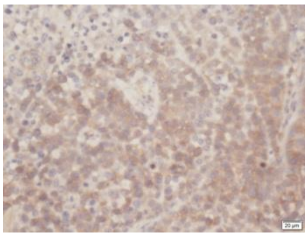	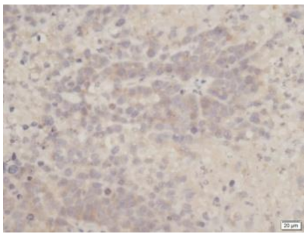
Treated ×100 (100μm)	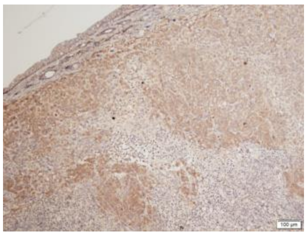	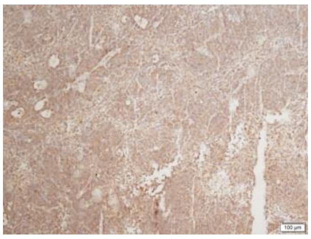	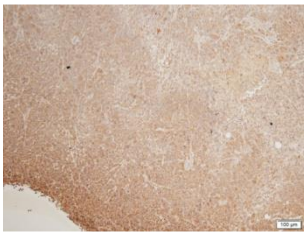
Treated ×400 (20μm)	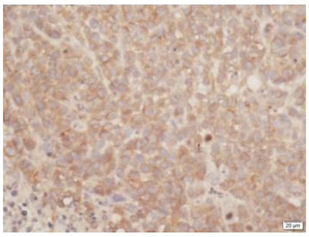	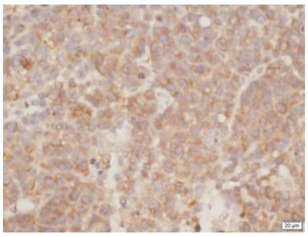	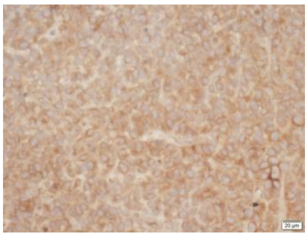
AKT	n1	n2	n3
Control ×100 (100μm)	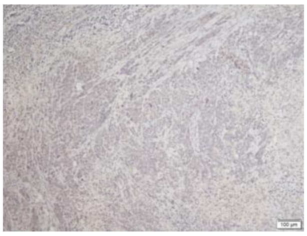	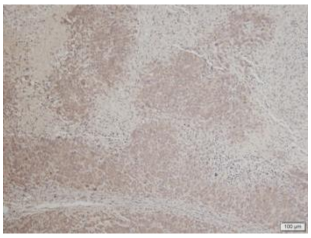	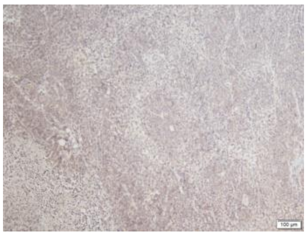
Control ×400 (20μm)	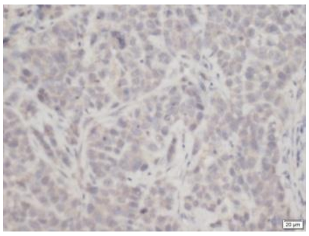	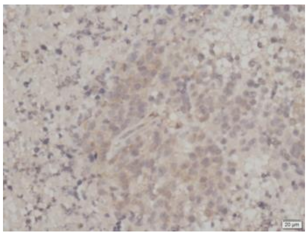	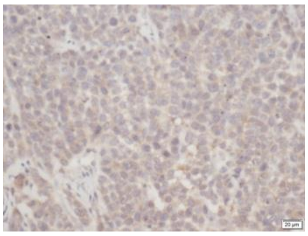

Treated<br>×100<br>(100μm)	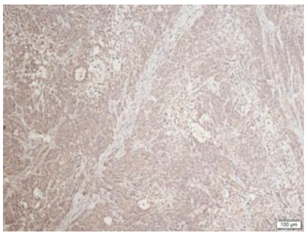	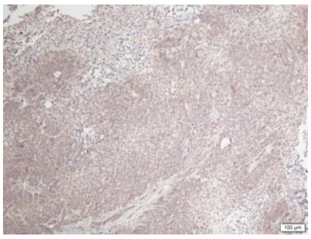	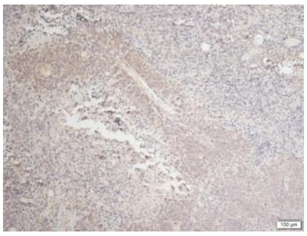
Treated<br>×400<br>(20μm)	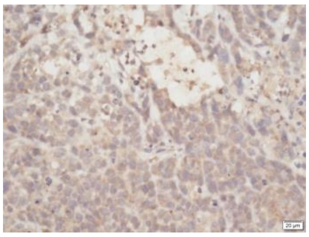	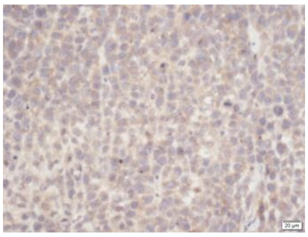	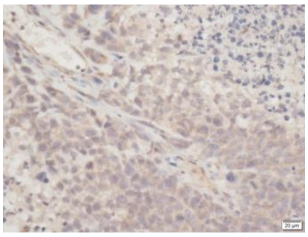
BAX	n1	n2	n3
Control<br>×100<br>(100μm)	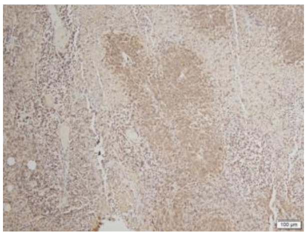	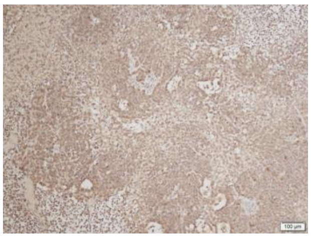	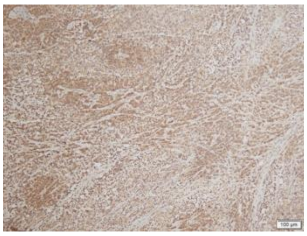
Control<br>×400<br>(20μm)	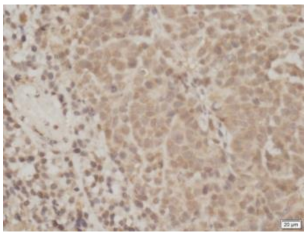	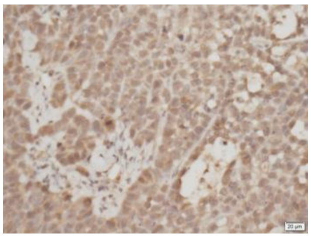	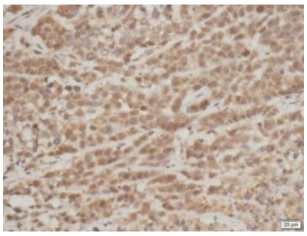
Treated<br>×100<br>(100μm)	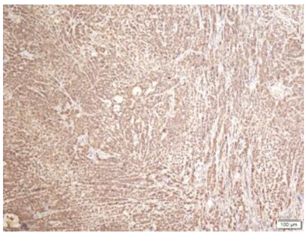	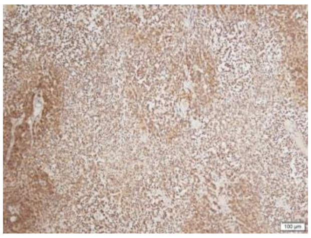	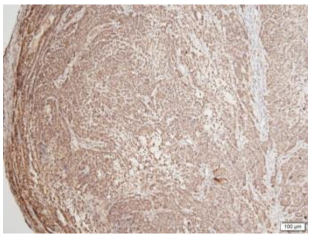
Treated<br>×400<br>(20μm)	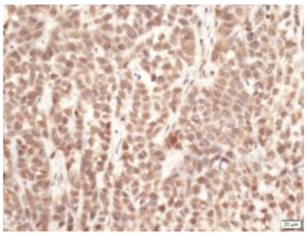	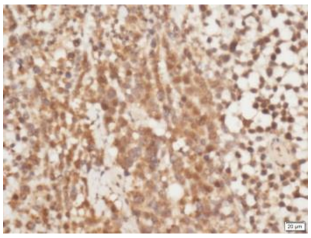	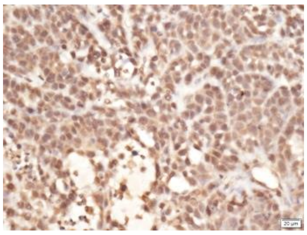
Caspase 3	n1	n2	n3
Control<br>×100<br>(100μm)	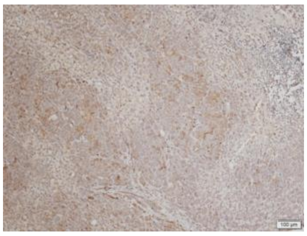	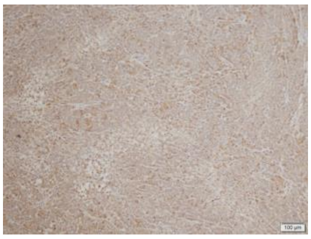	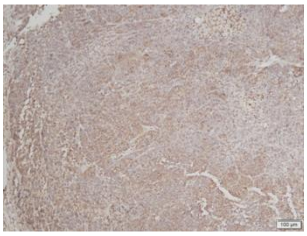

Control ×400 (20μm)	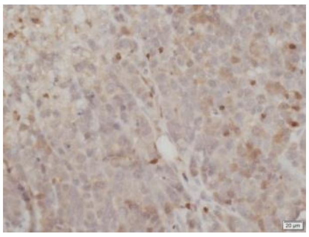	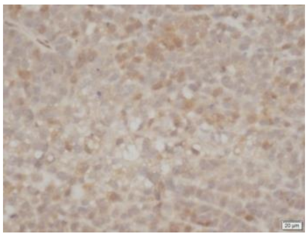	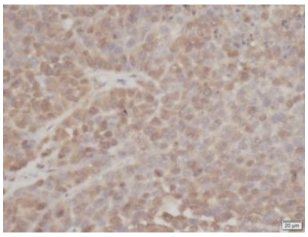
Treated ×100 (100μm)	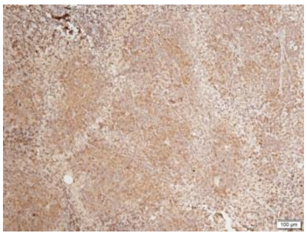	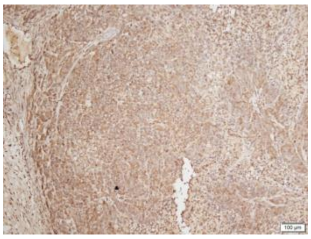	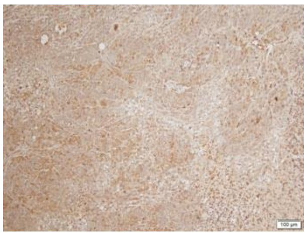
Treated ×400 (20μm)	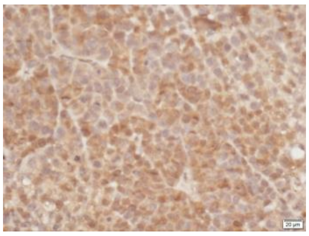	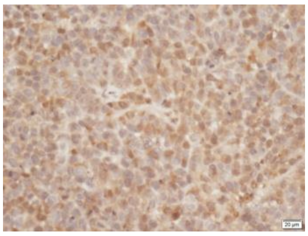	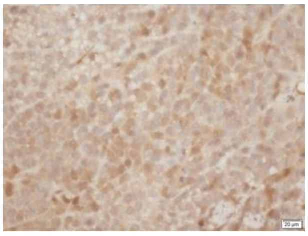
Caspase 9	n1	n2	n3
Control ×100 (100μm)	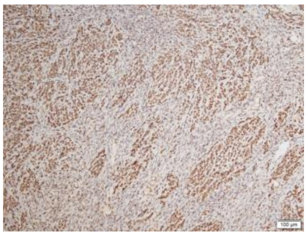	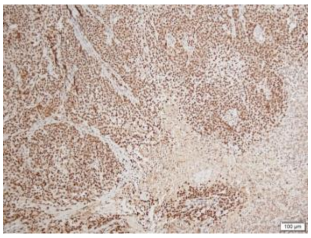	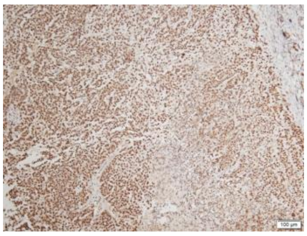
Control ×400 (20μm)	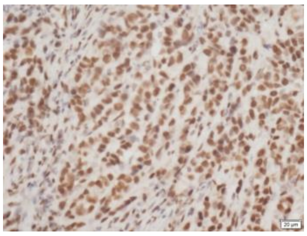	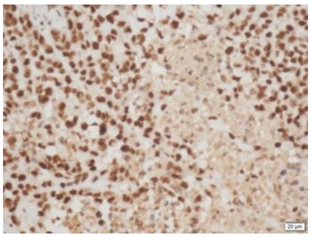	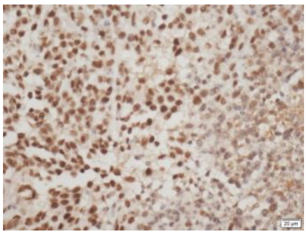
Treated ×100 (100μm)	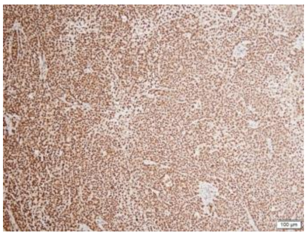	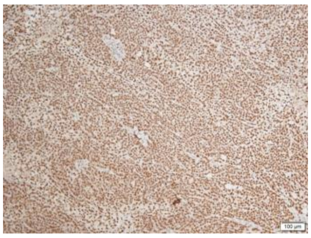	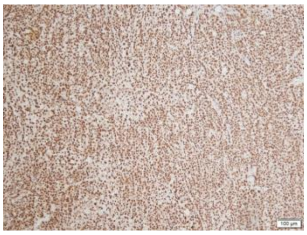
Treated ×400 (20μm)	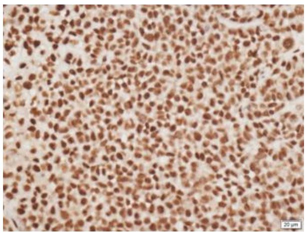	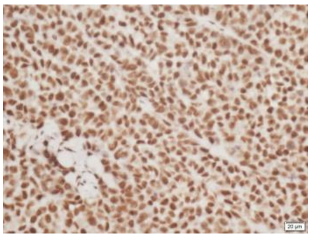	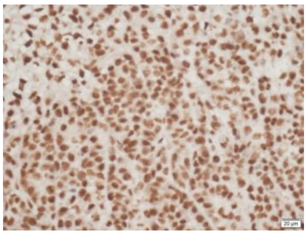
Ki67	n1	n2	n3

Control ×100 (100μm)	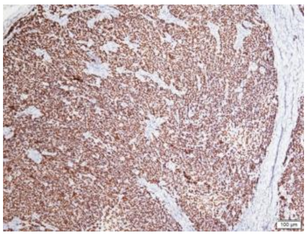	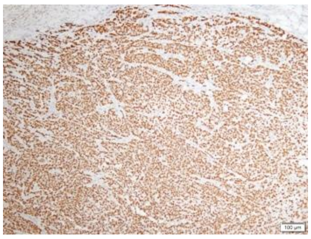	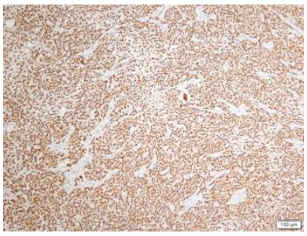
Control ×400 (20μm)	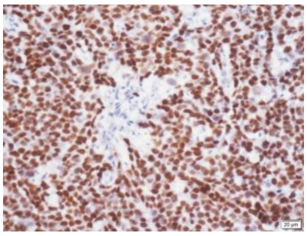	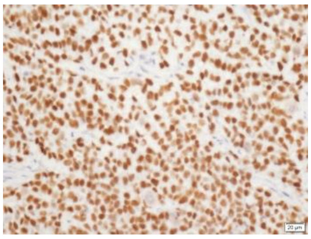	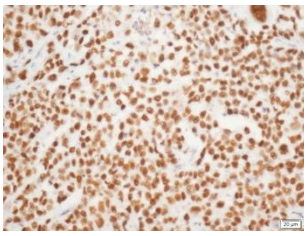
Treated ×100 (100μm)	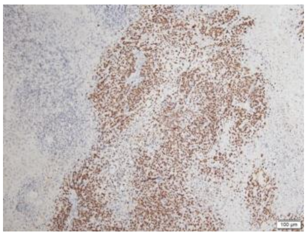	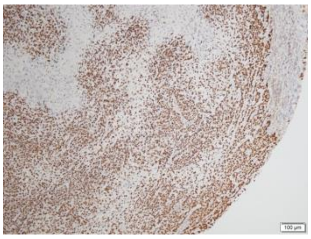	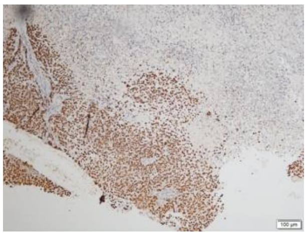
Treated ×400 (20μm)	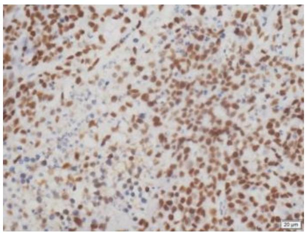	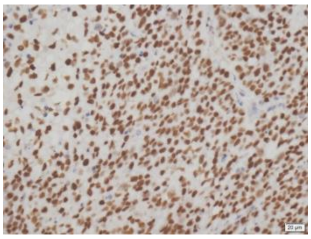	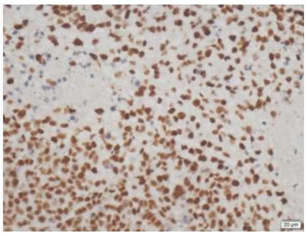
LC3A	n1	n2	n3
Control ×100 (100μm)	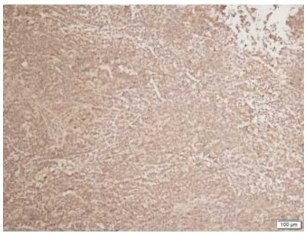	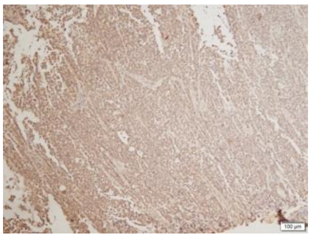	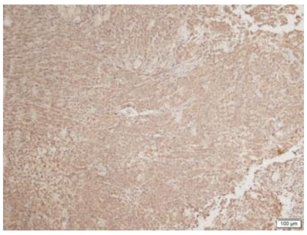
Control ×400 (20μm)	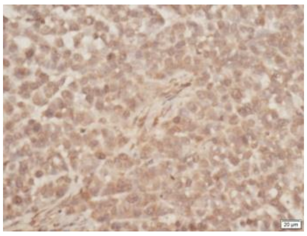	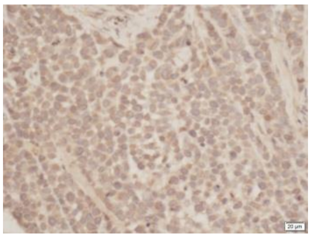	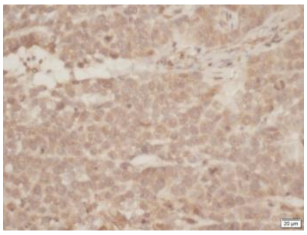
Treated ×100 (100μm)	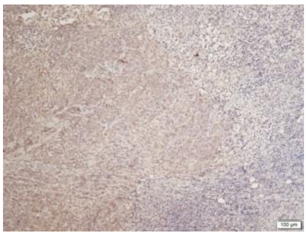	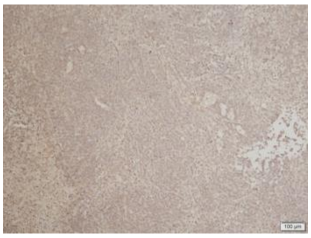	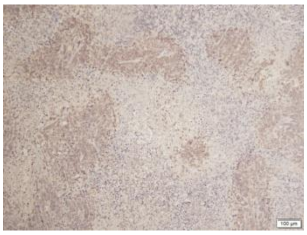

Treated<br>×400<br>(20μm)	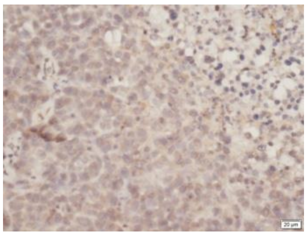	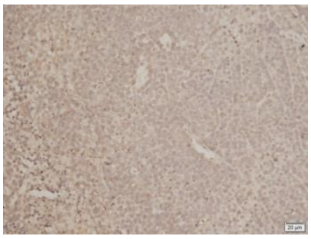	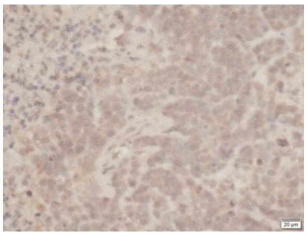

Table S3. The images of the original western blots in three repetitions

Figure 2D			
Proteins	n1	n 2	n 3
CDK1	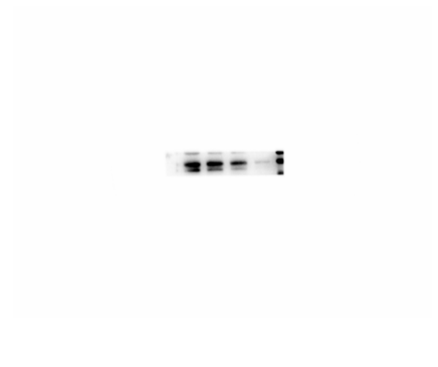	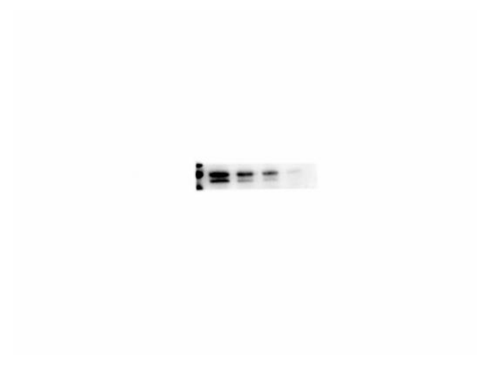	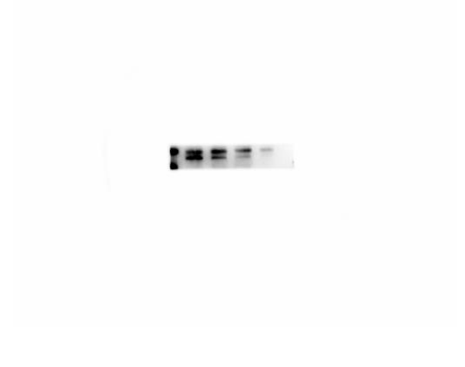
Cyclin B1		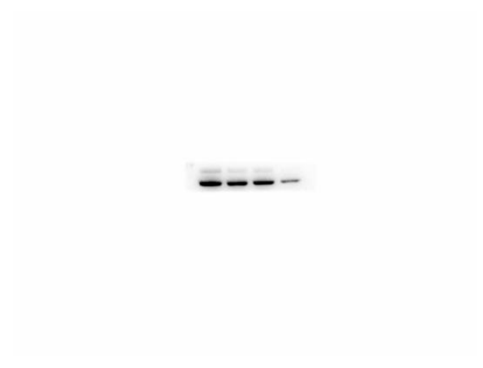	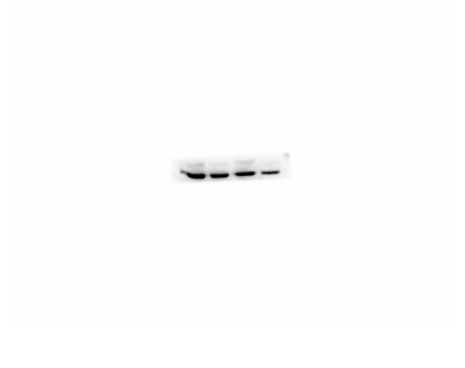
P21	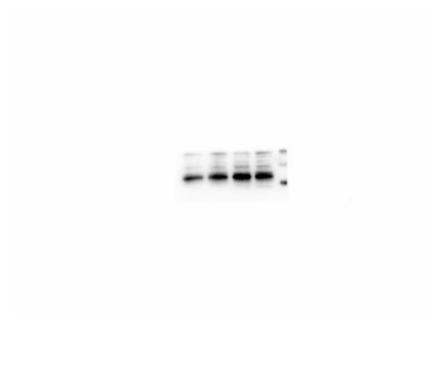	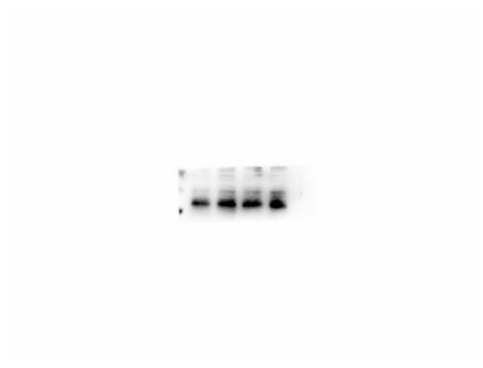	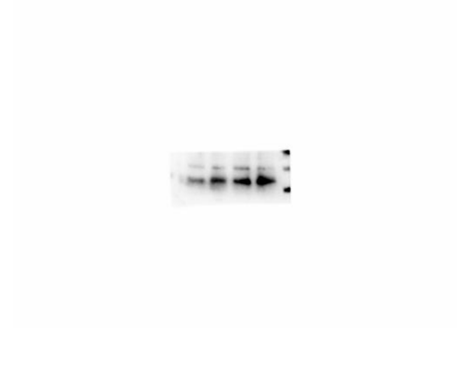
β-actin			
Figure 2E			

CDK1	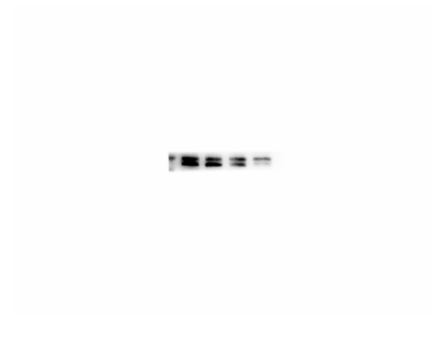	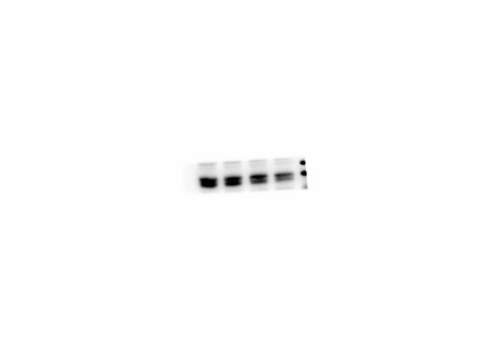	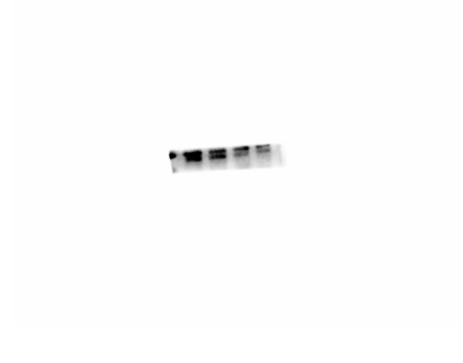
Cyclin B1	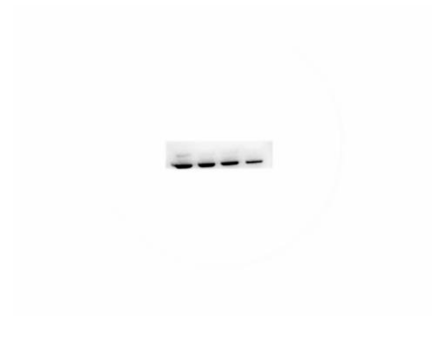	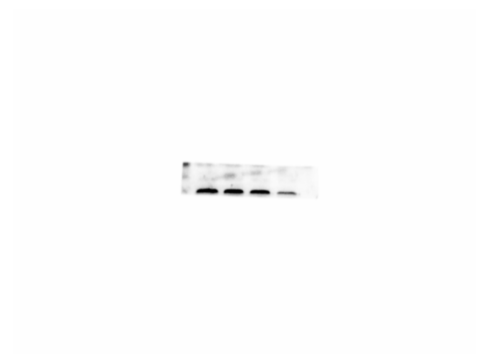	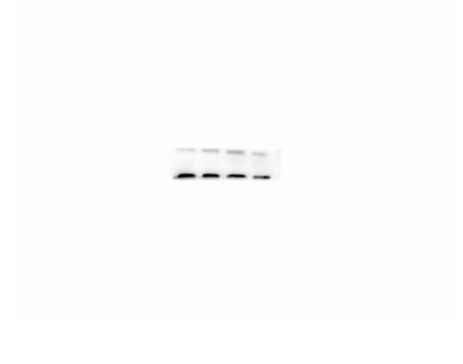
P21	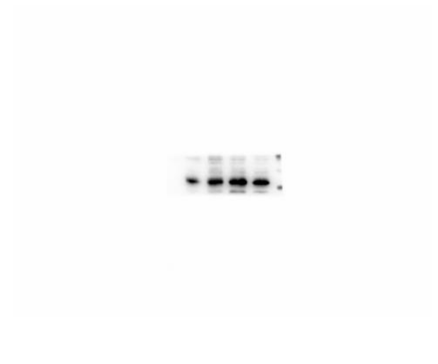	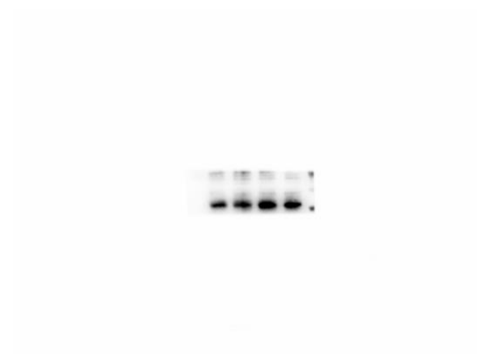	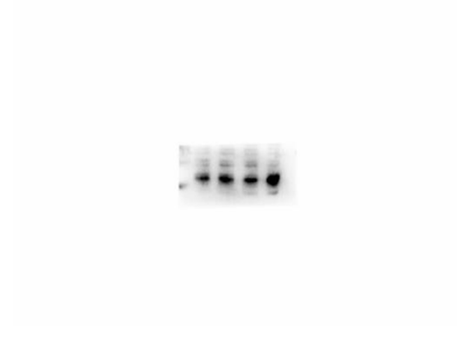
β-actin		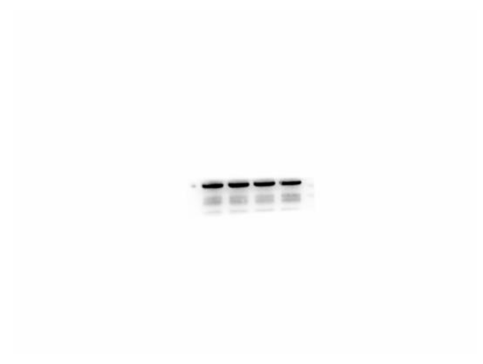	

Figure 2F			
CDK1	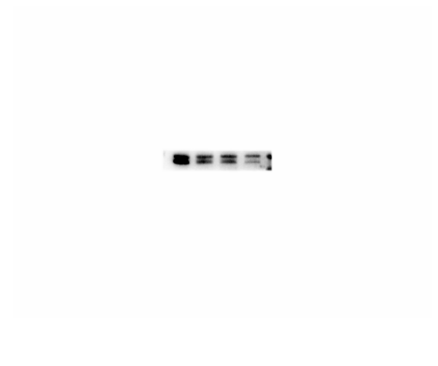	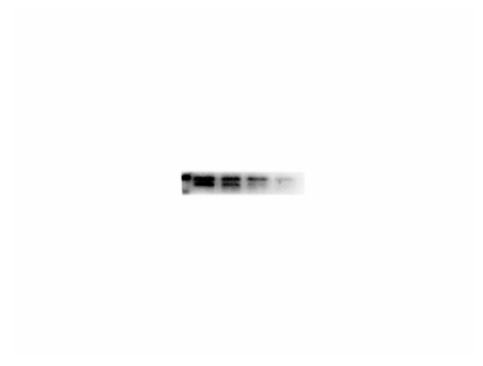	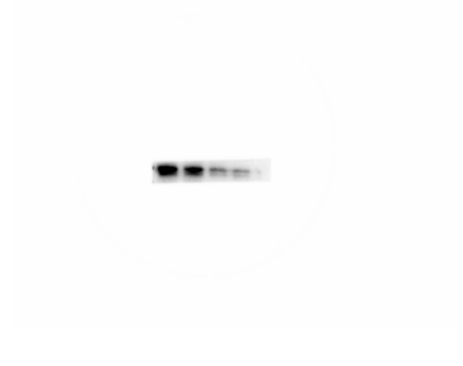
Cyclin B1	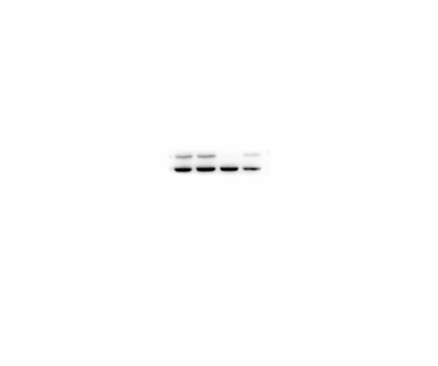	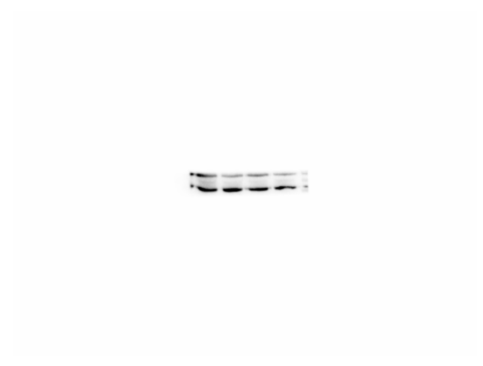	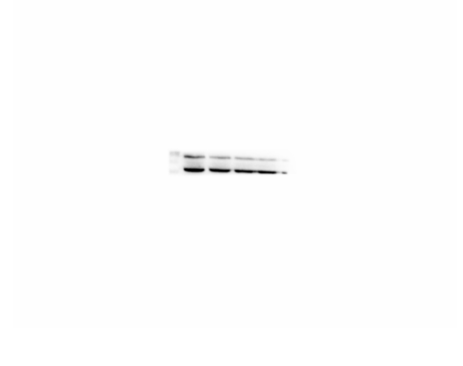
P21	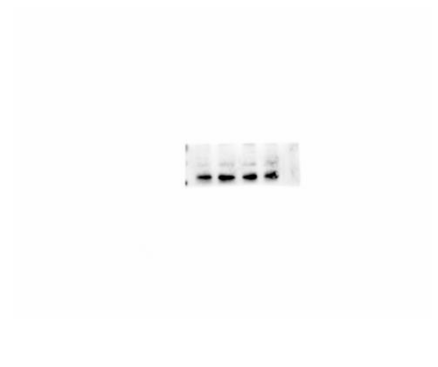	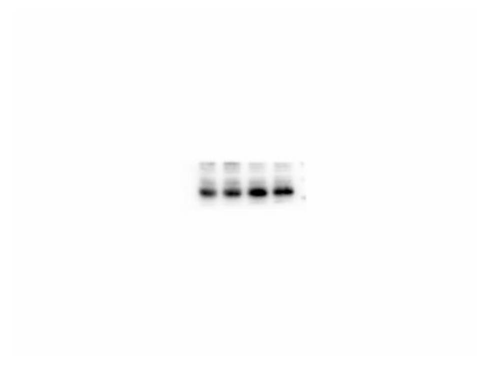	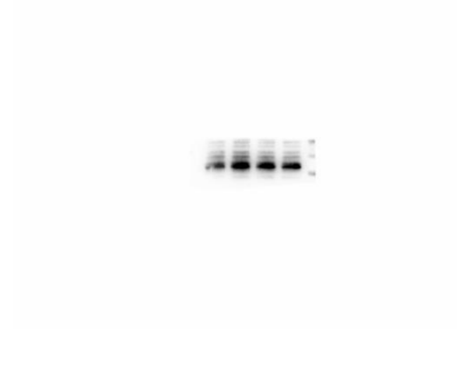
β-actin			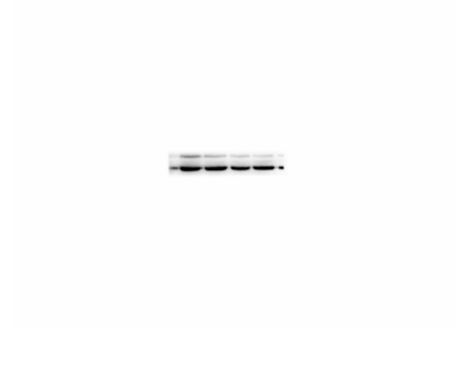

Figure 3E (A549)			
Bcl2	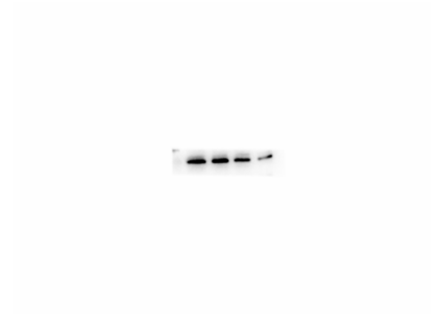	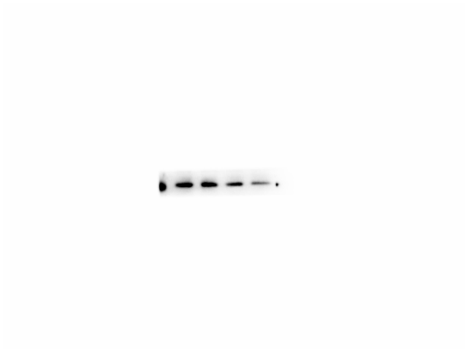	
Bax	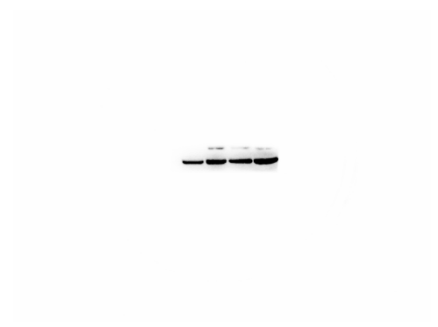	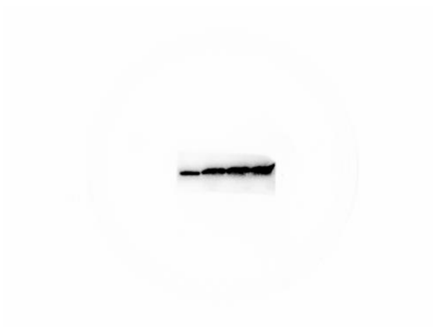	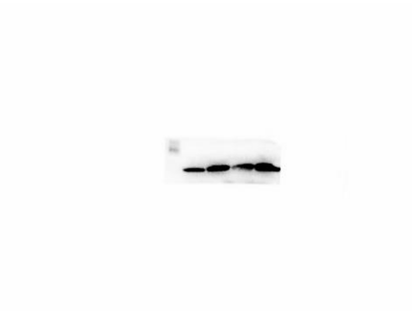
Cle-caspase3	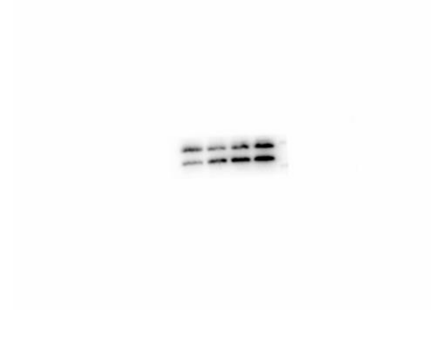	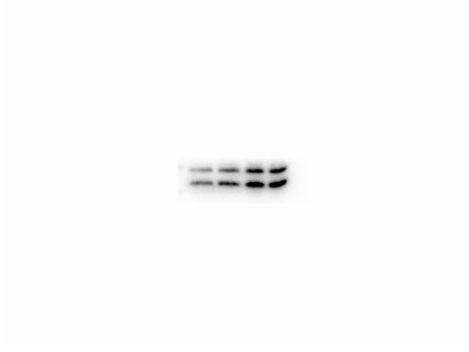	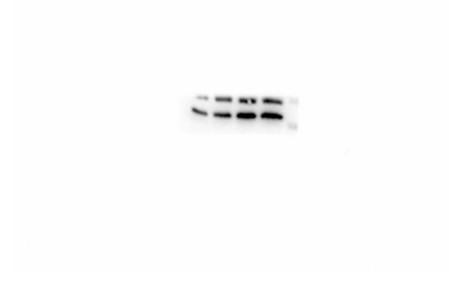
Cle-caspase9	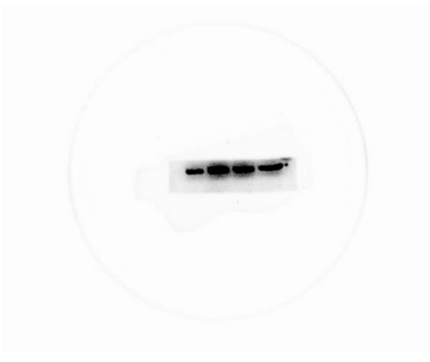	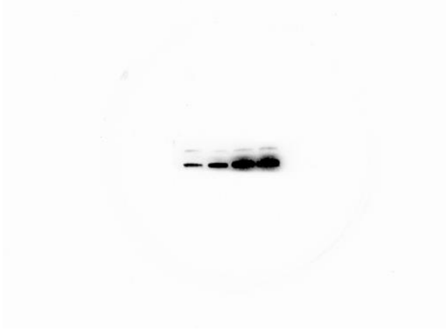	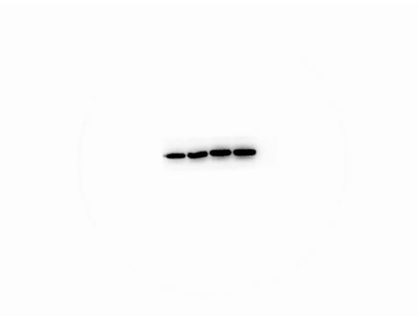

β-actin	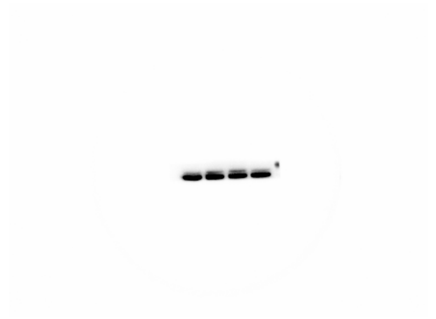		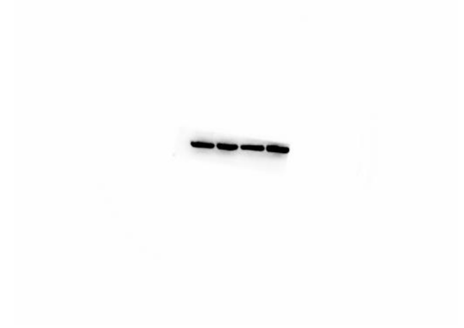

Figure 3E (H1299)			
Bcl2	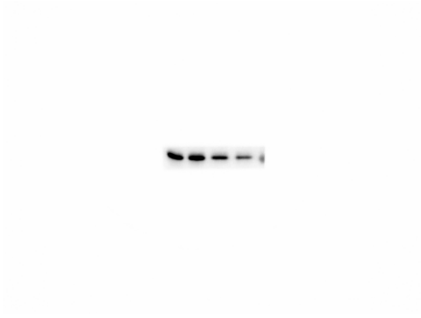	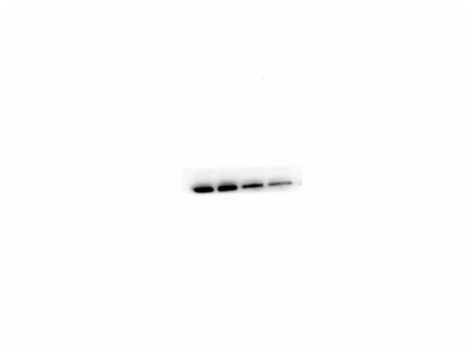	
Bax	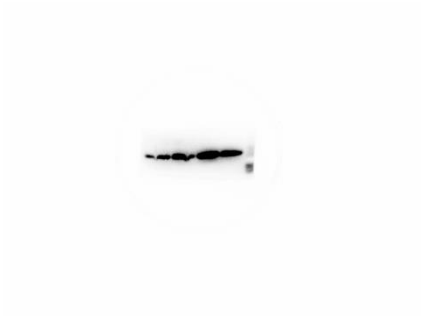	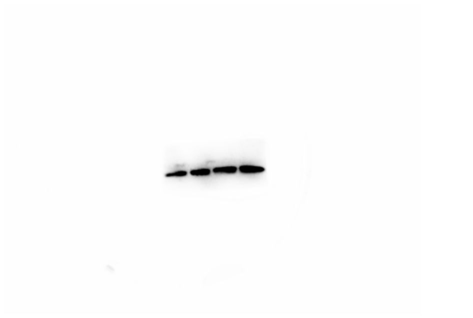	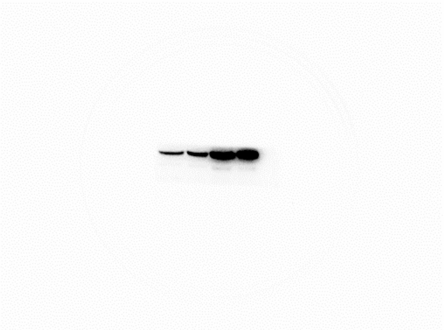
Cle-caspase3	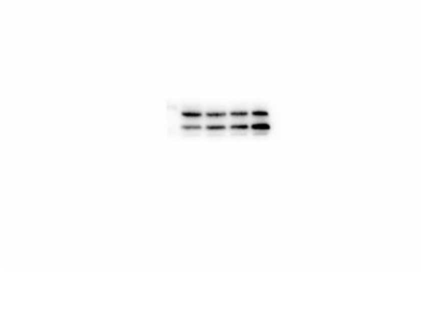	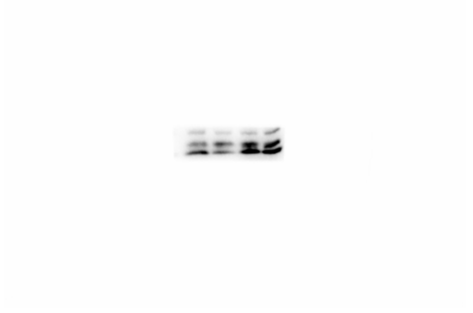	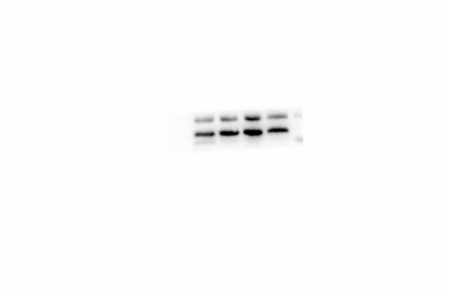
Cle-caspase9	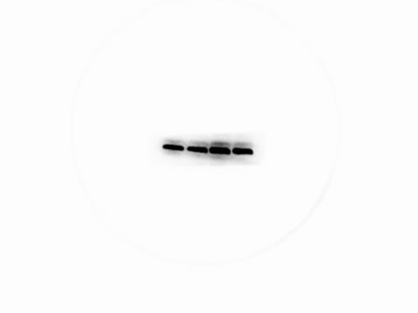	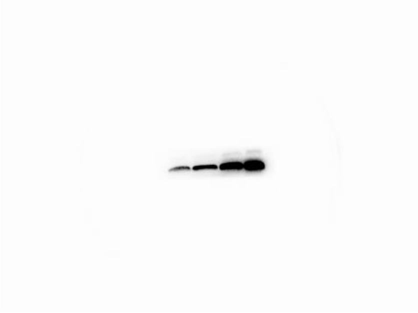	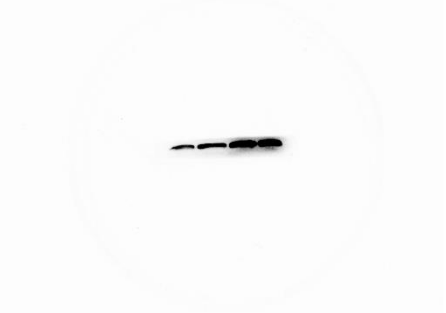

β-actin		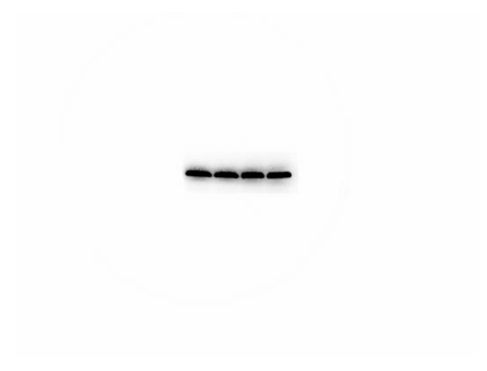	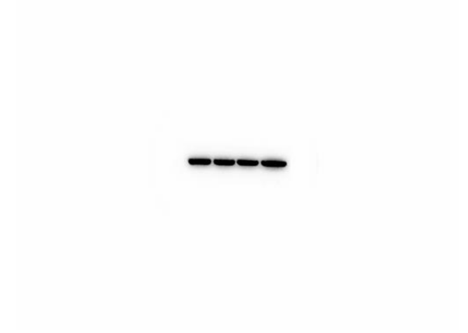

Figure 3E (H226)			
Bcl2	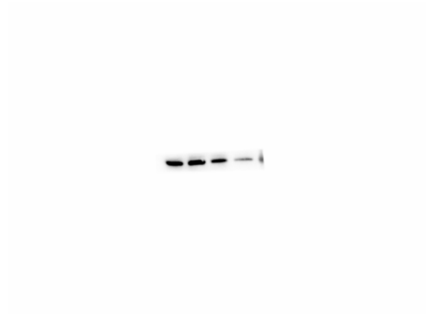		
Bax	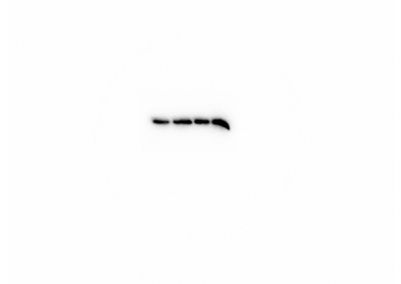		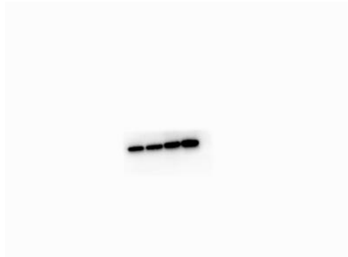
Cle-caspase3	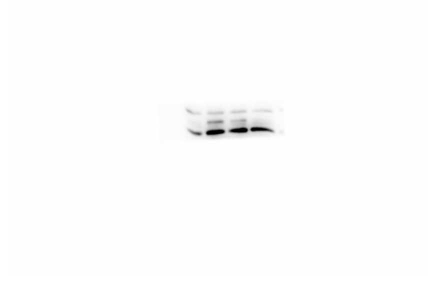	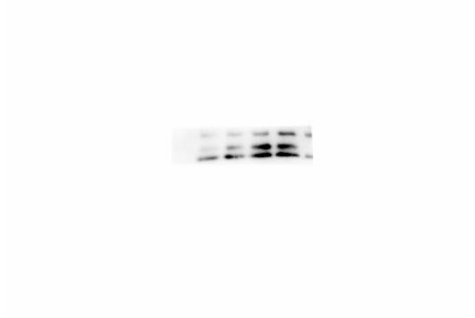	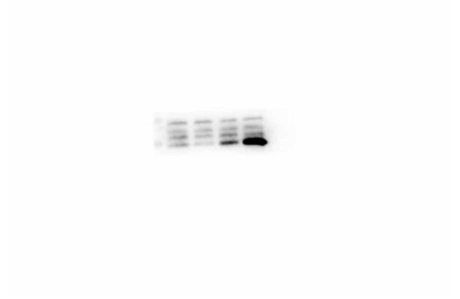
Cle-caspase9			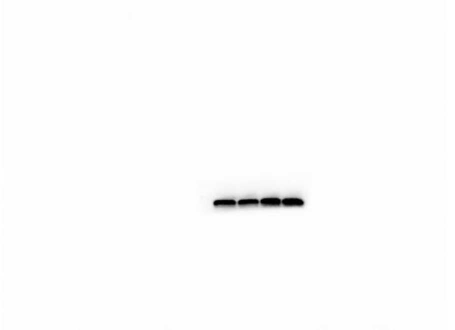

β-actin	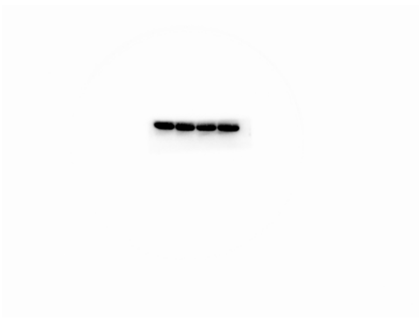	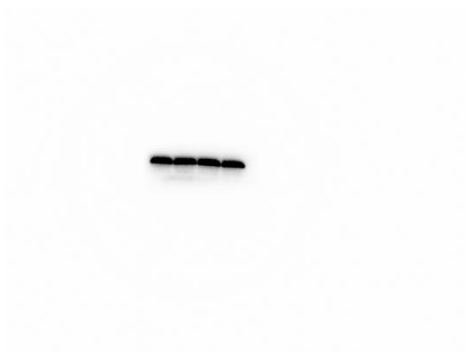	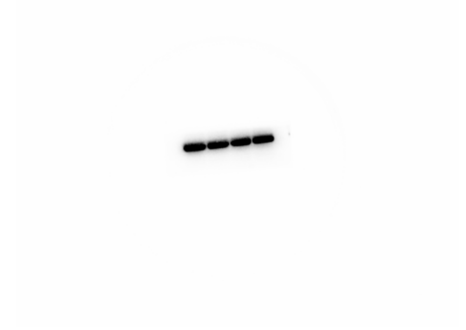

Figure 4B (A549)			
Beclin1	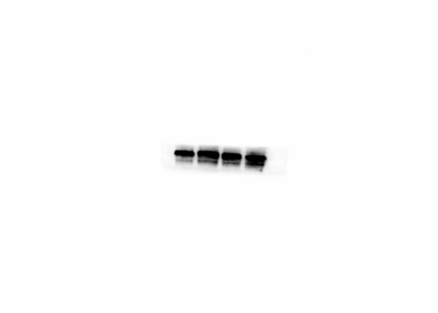	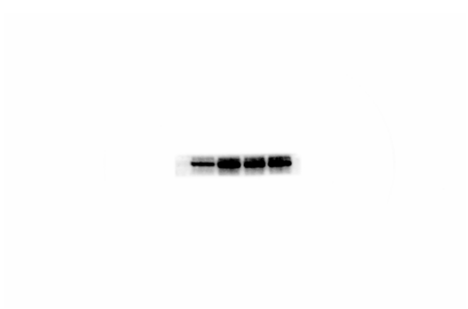	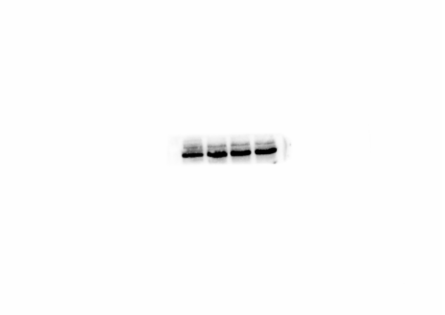
Lc3	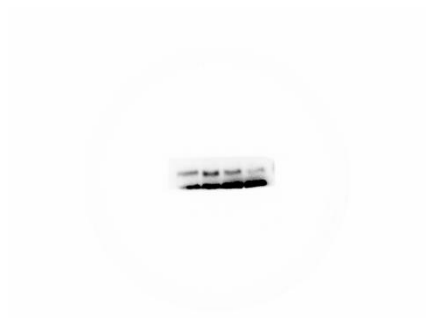	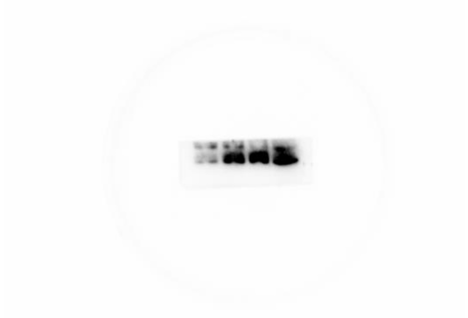	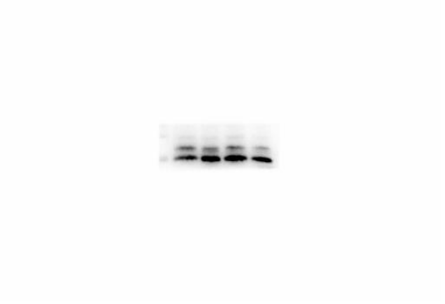
P62	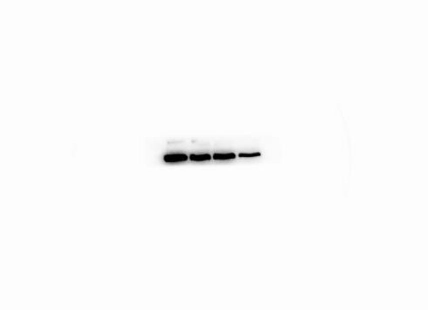	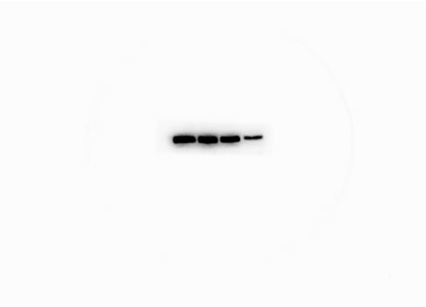	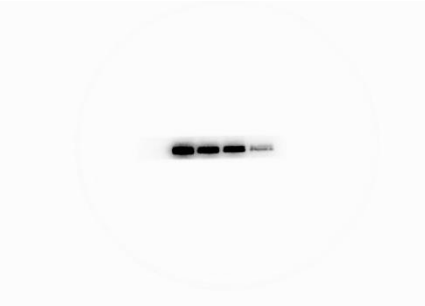
β-actin	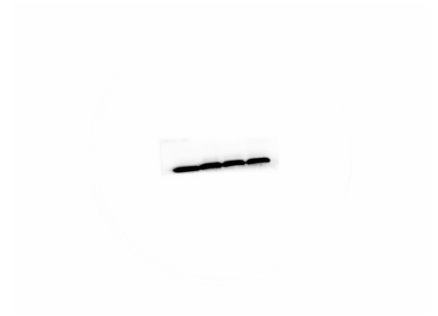	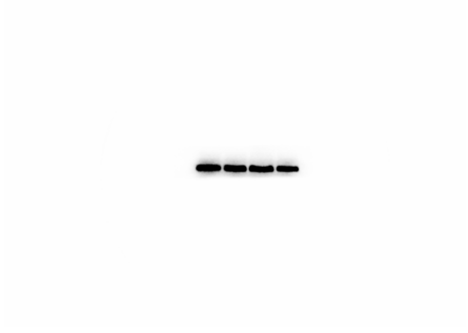	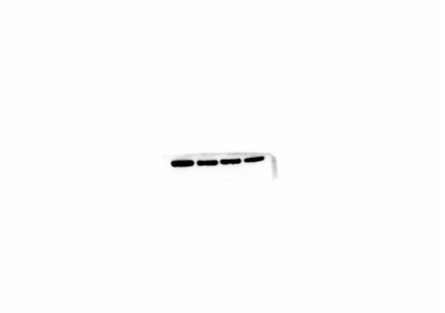

Figure 4B (H1299)			
Beclin1			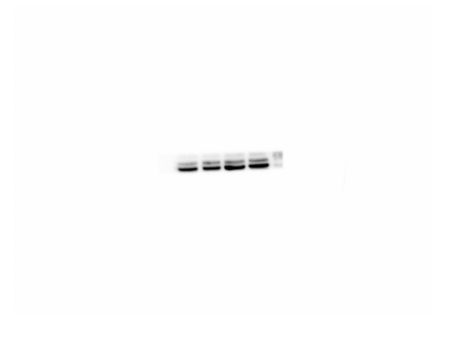
Lc3	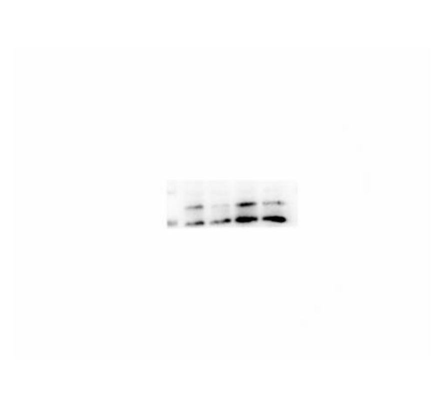	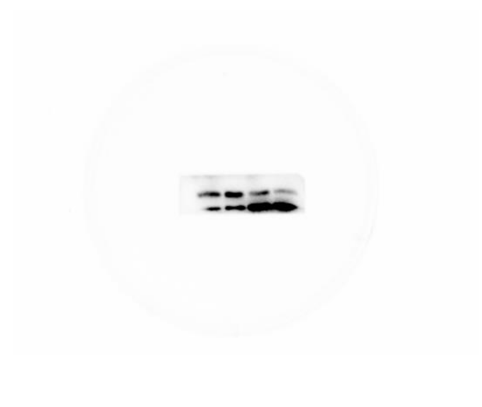	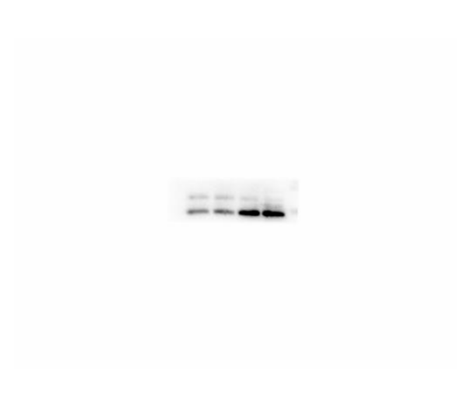
P62			
β-actin			

Figure 4B (H226)			
Beclin1	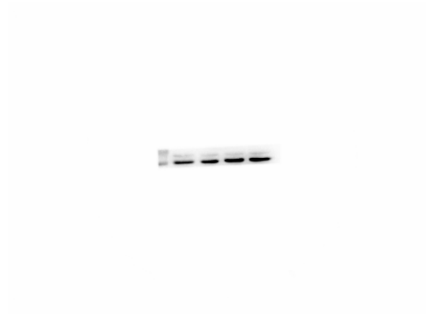	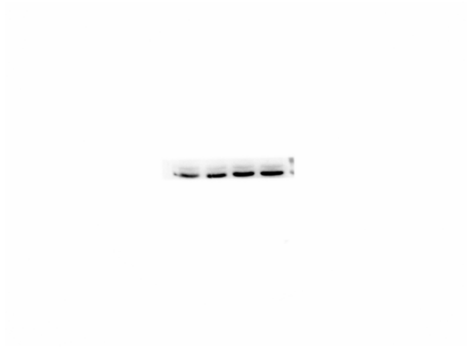	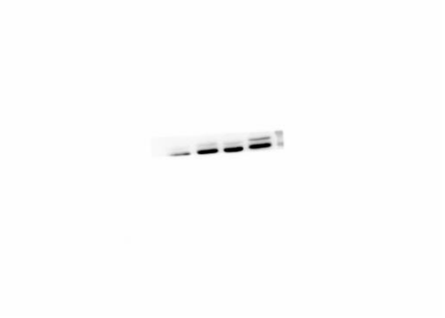
LC3	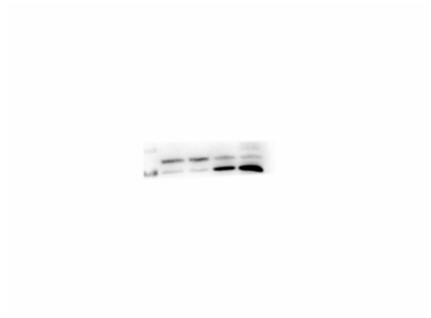	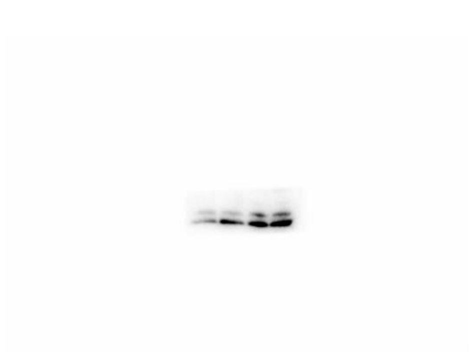	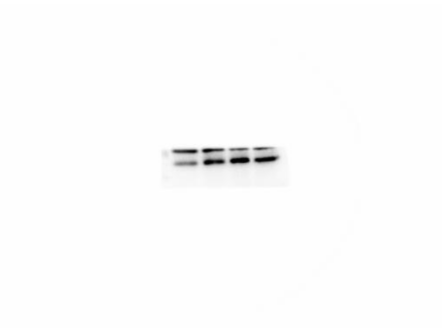
P62	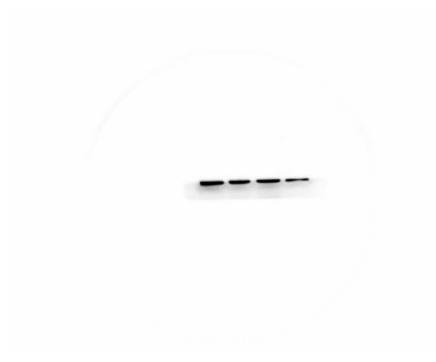		
β-actin			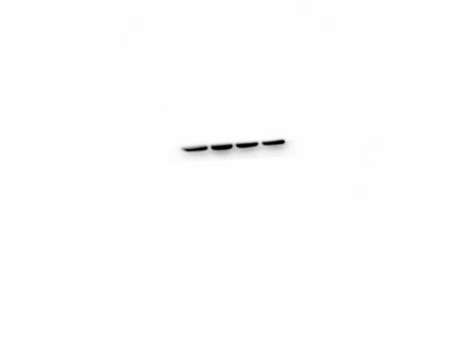

Figure 4D (A549)			
Beclin1	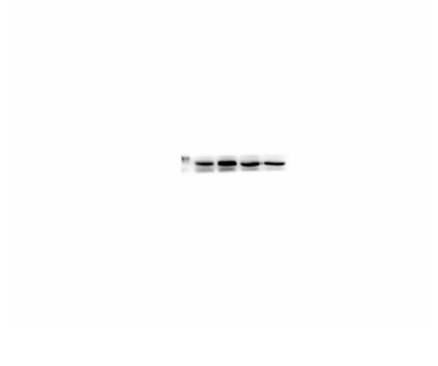	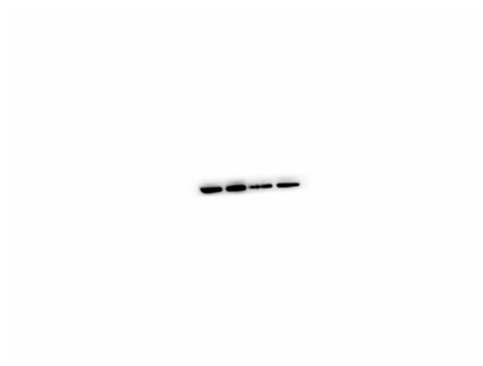	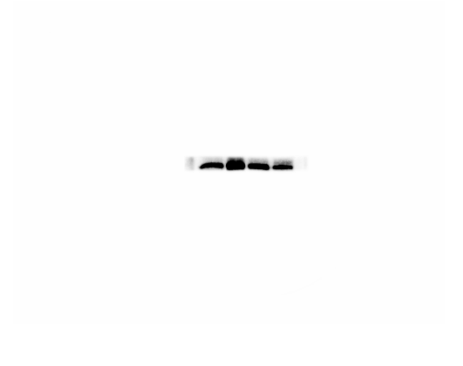
Lc3	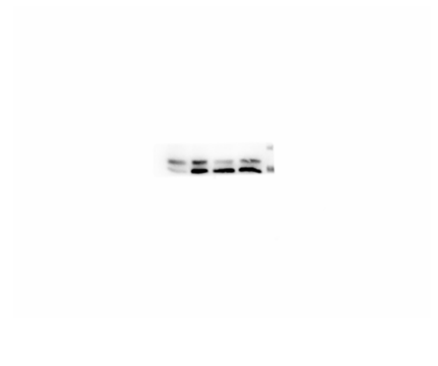	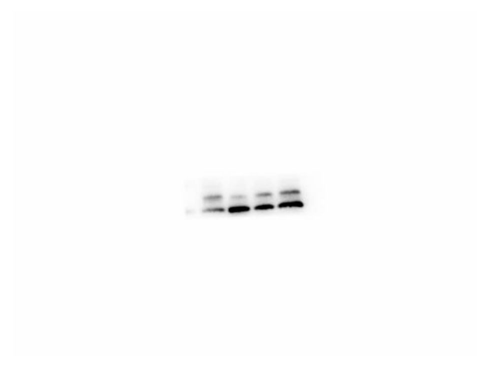	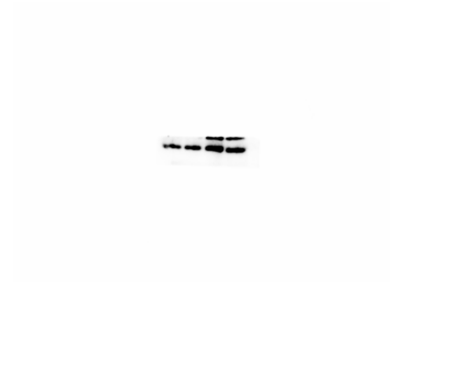
P62	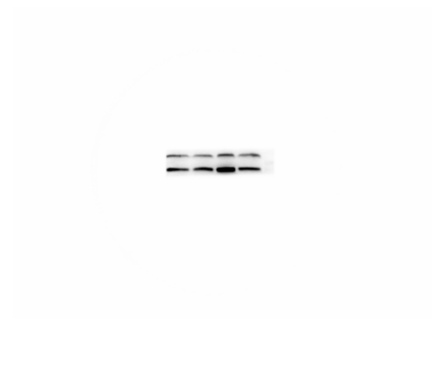	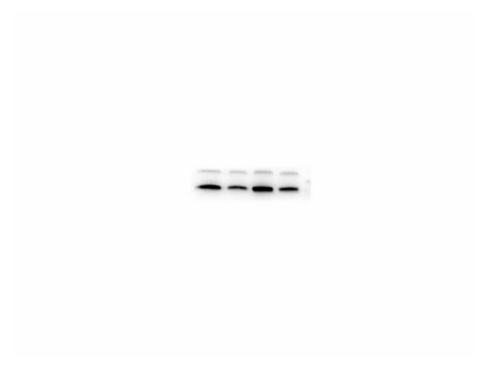	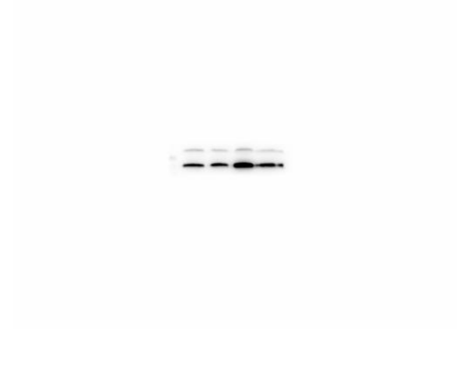
β-actin	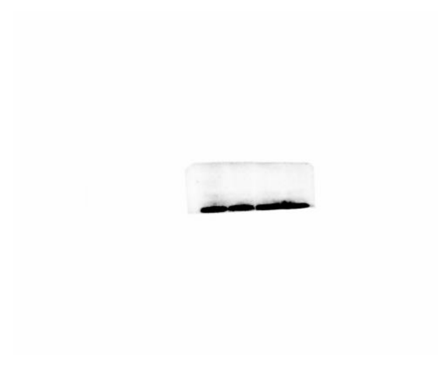	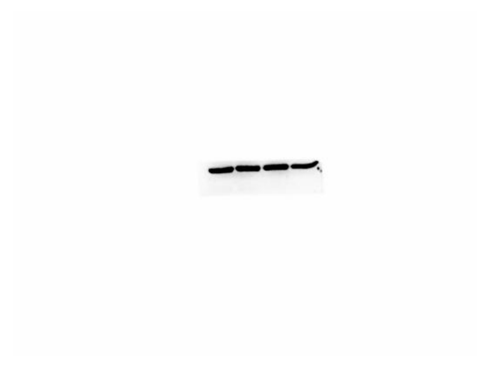	

Figure 4 D (H1299)			
Beclin1	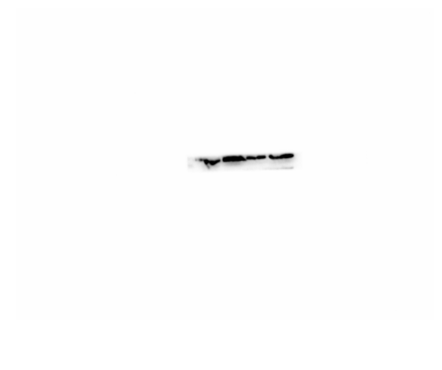	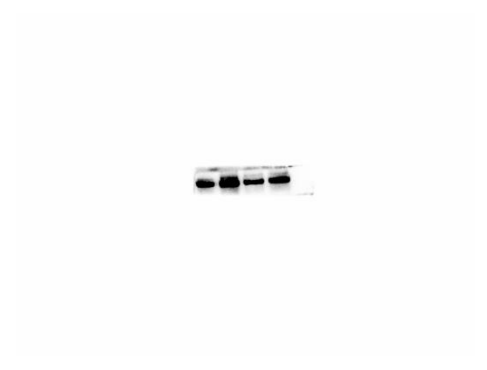	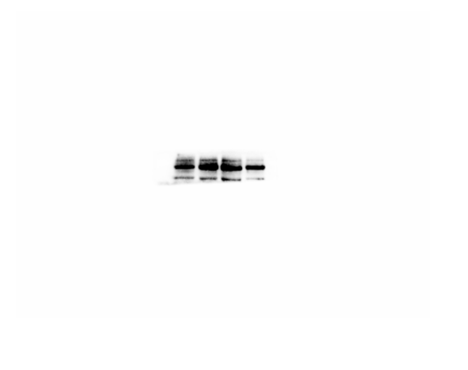
Lc3	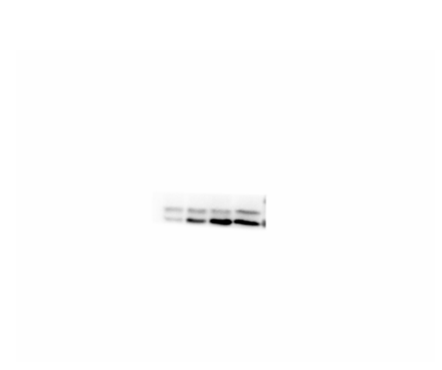	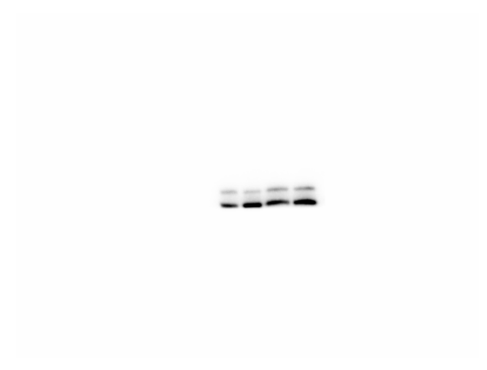	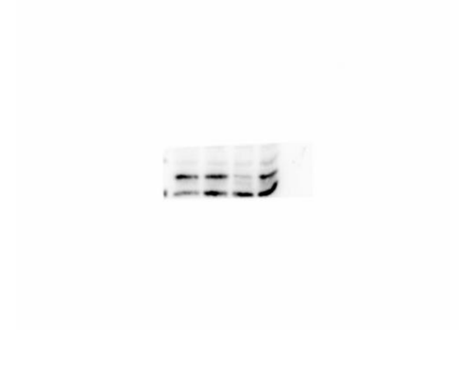
P62	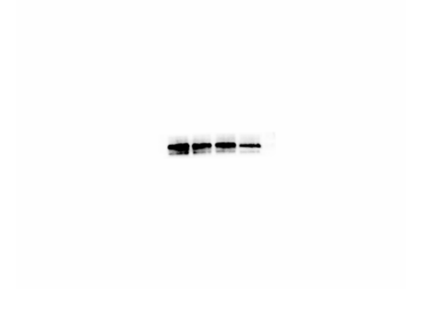	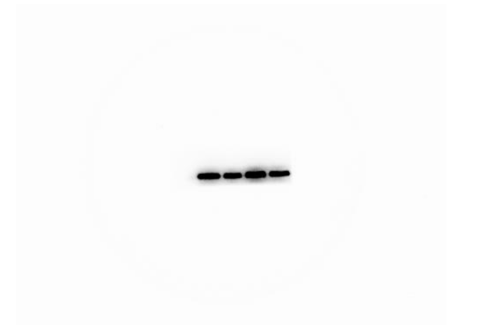	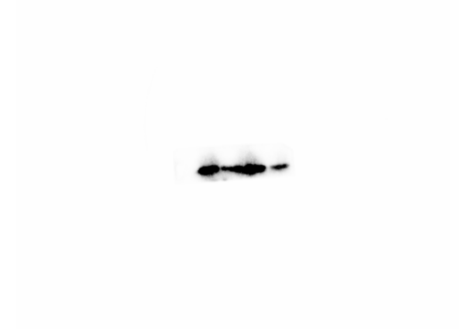
β-actin	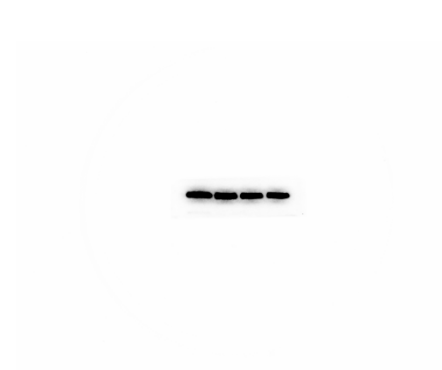	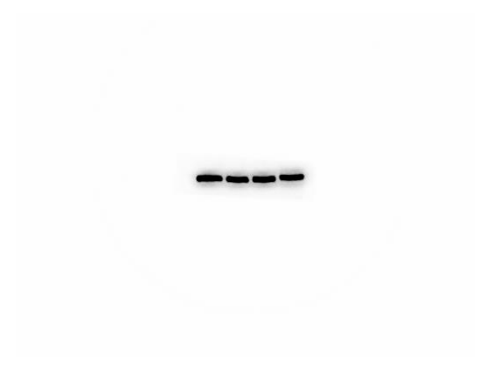	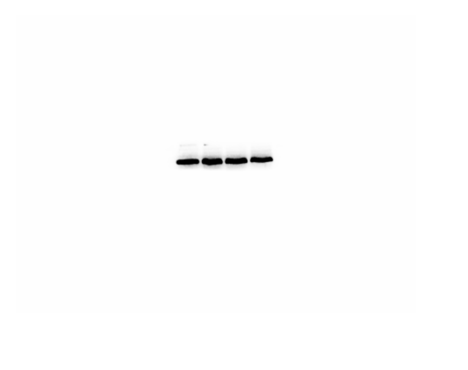

Figure 4D (H226)			
Beclin1	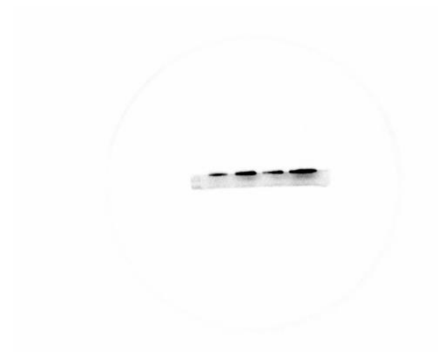	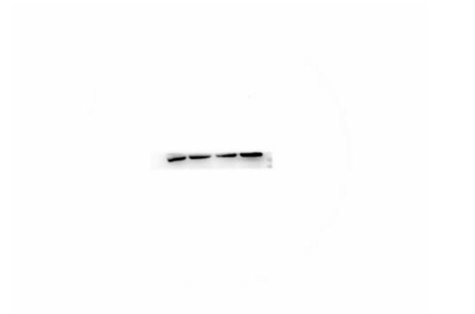	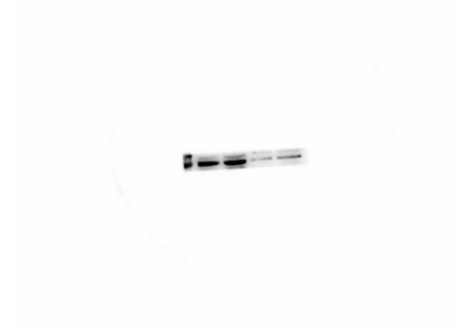
LC3	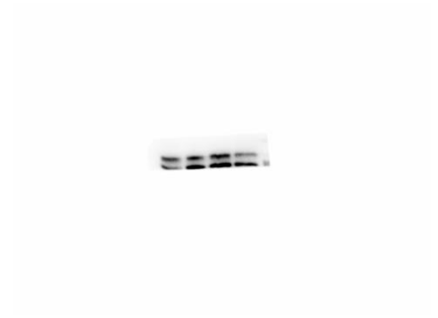	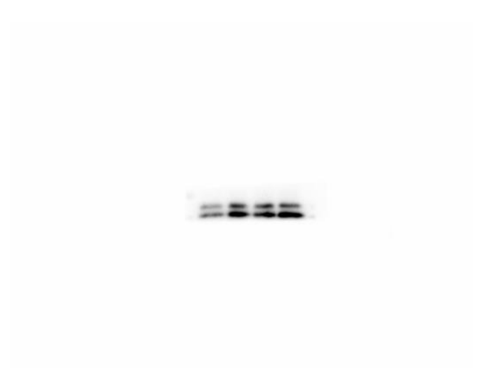	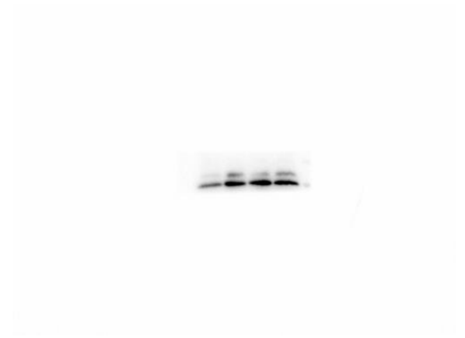
P62	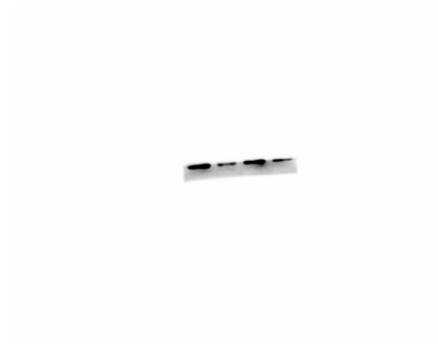	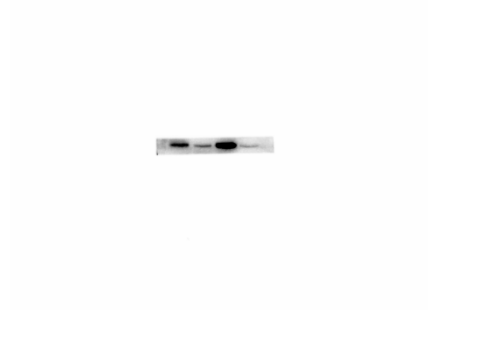	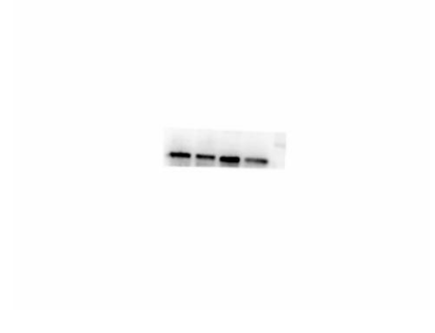
β-actin	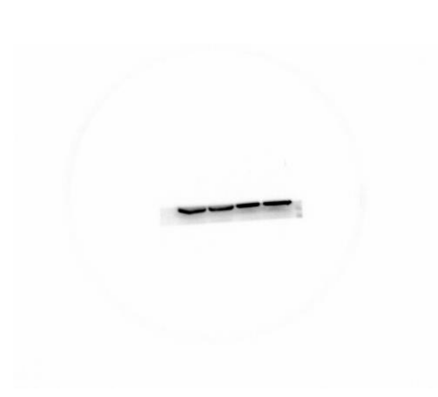		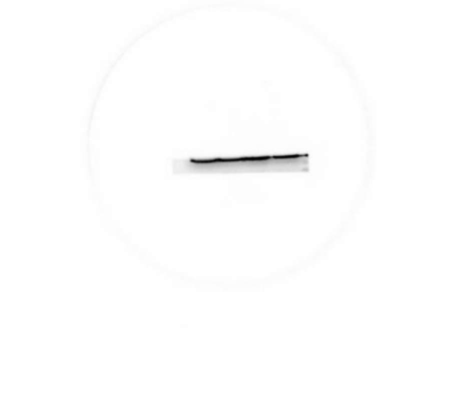

Figure 4F (A549)			
Bcl2	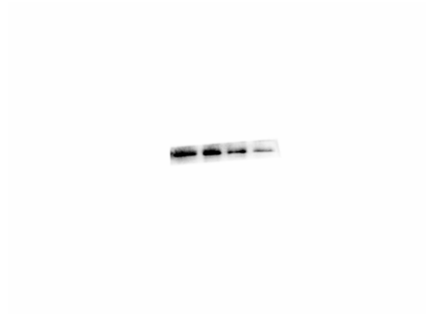	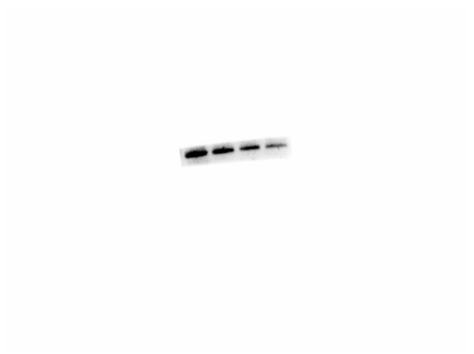	
β-actin	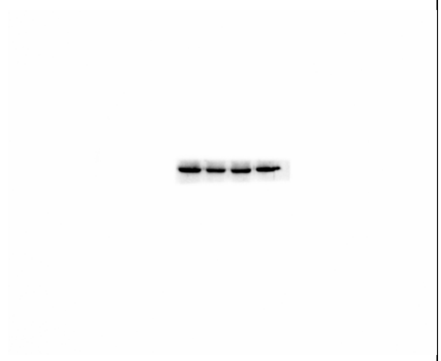	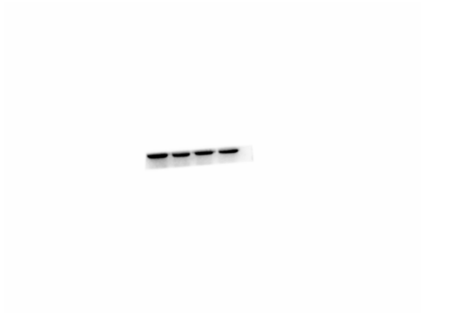	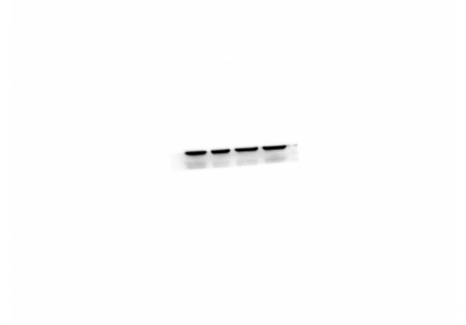

Figure 4F (H1299)			
Bcl2		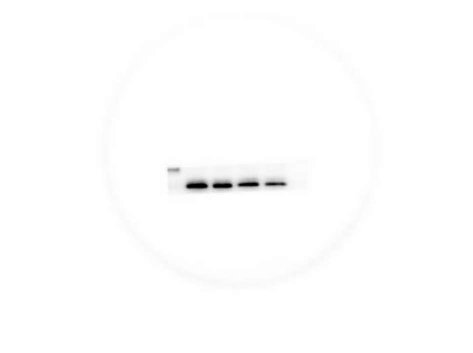	
β-actin			

Figure 4F (H226)			
Bcl2	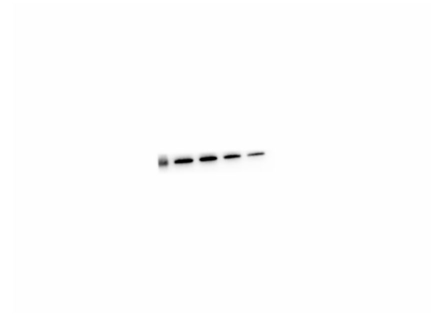	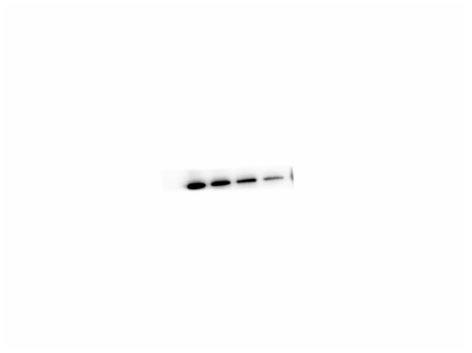	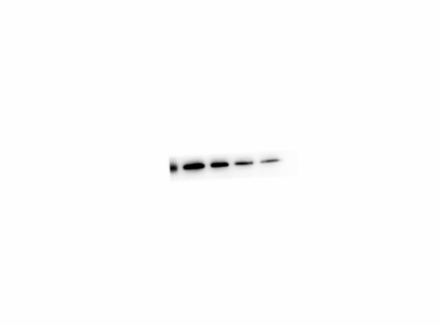
β-actin	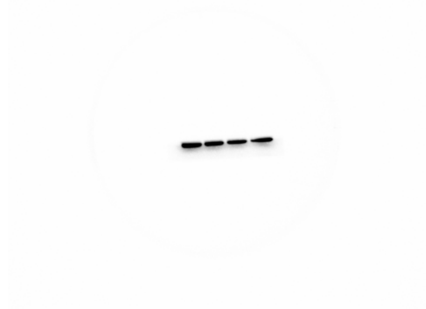		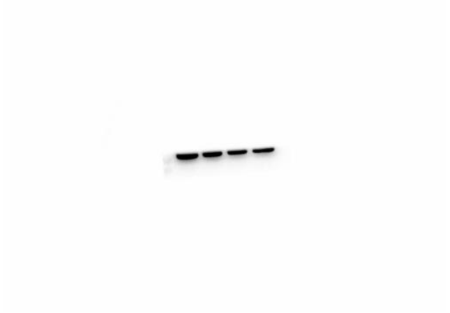

Figure 6A (A549)			
mTOR			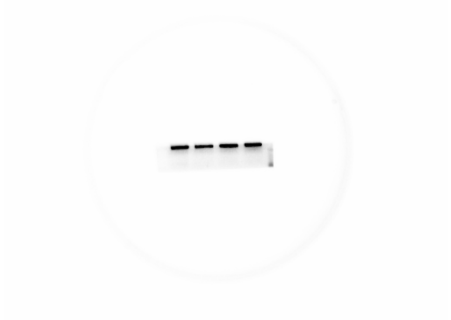
p-mTOR			
PI3K	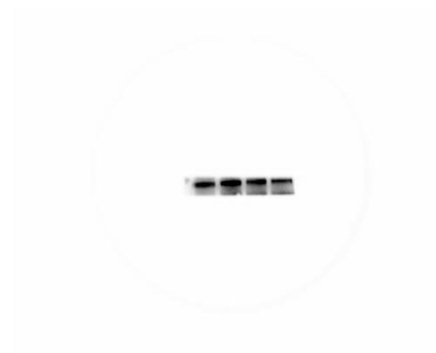	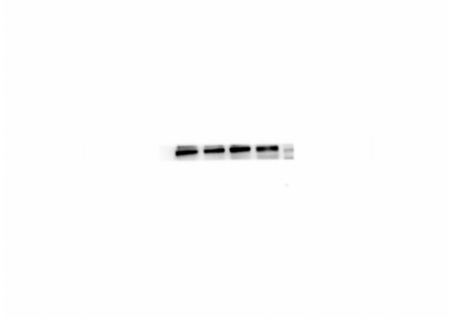	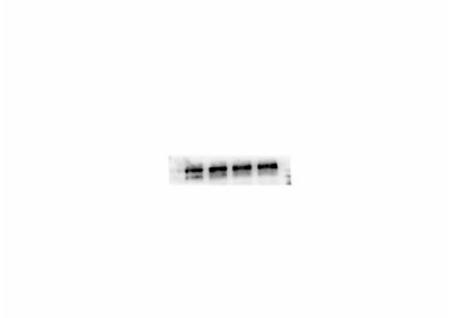
p-PI3K	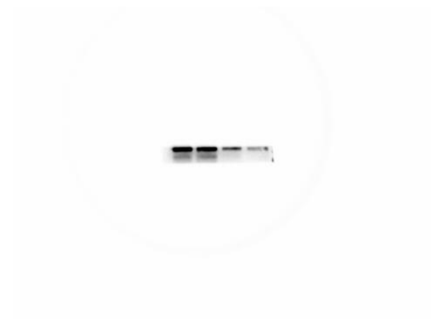	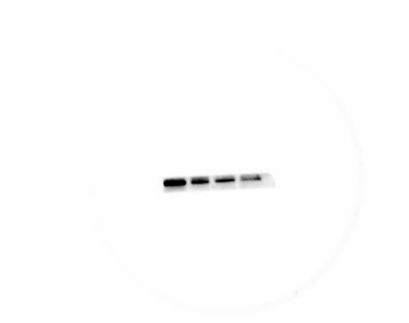	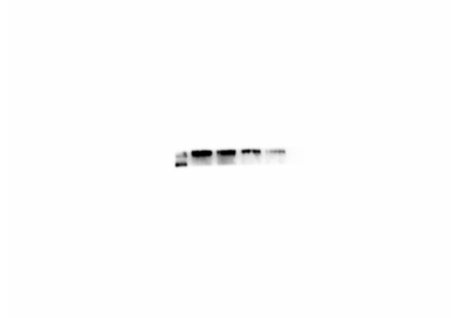

AKT	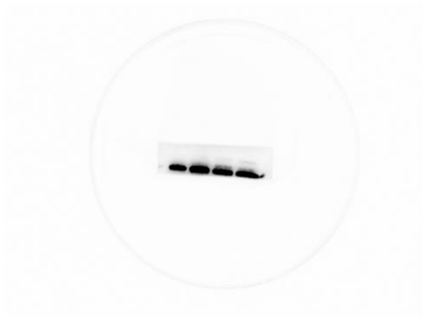	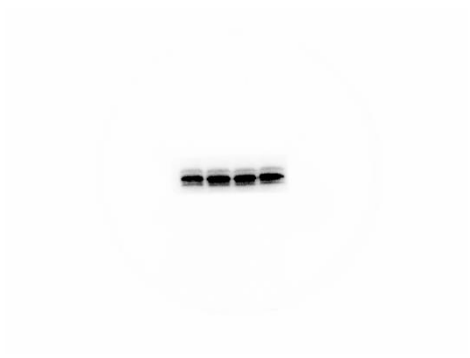	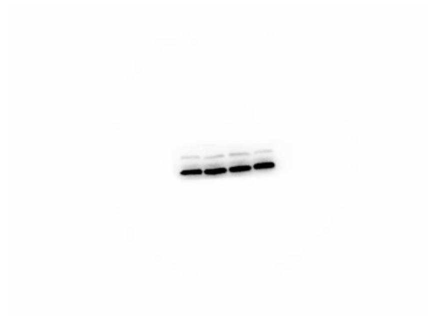
P-AKT	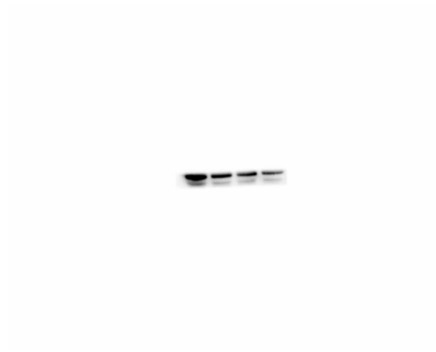	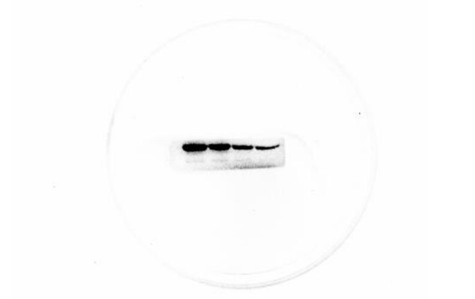	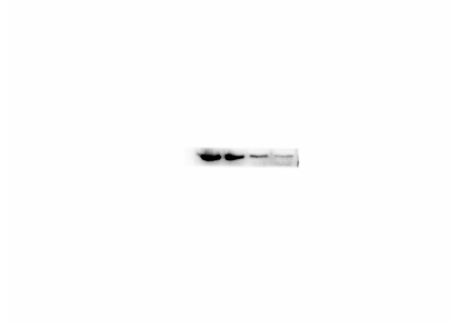
β-actin	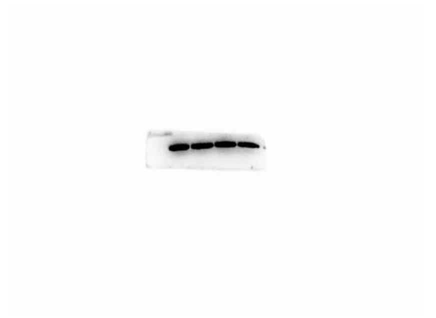	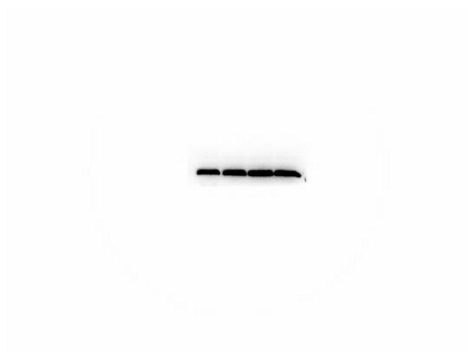	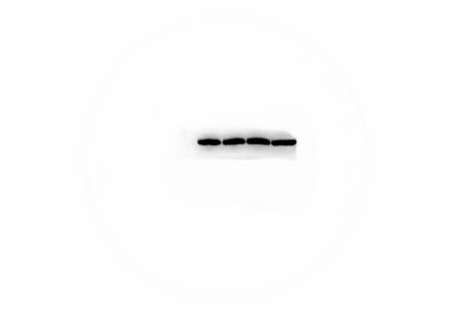

Figure 6A (H1299)			
mTOR			
p-mTOR			
PI3K	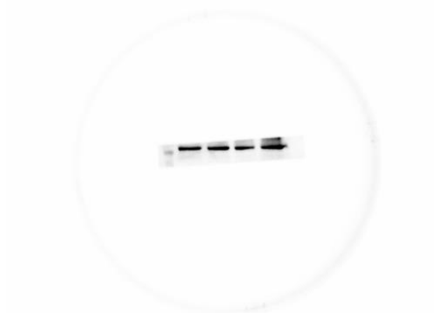		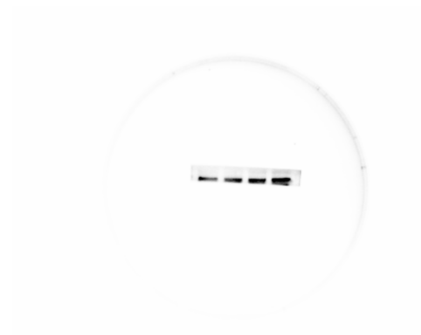
p-PI3K	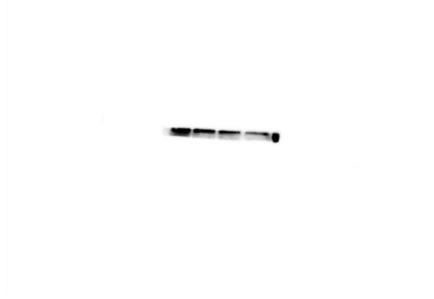	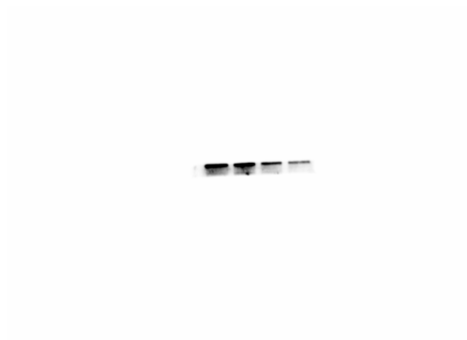	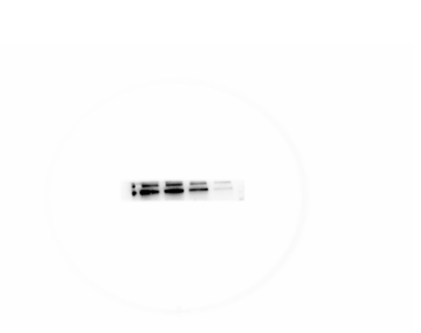

AKT	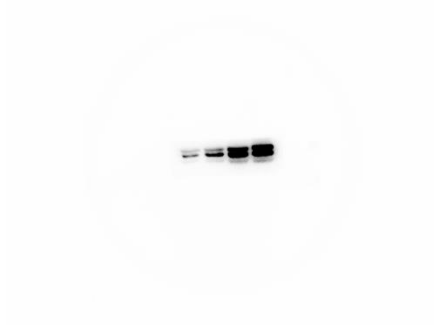	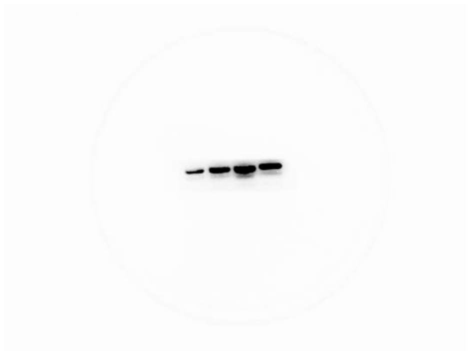	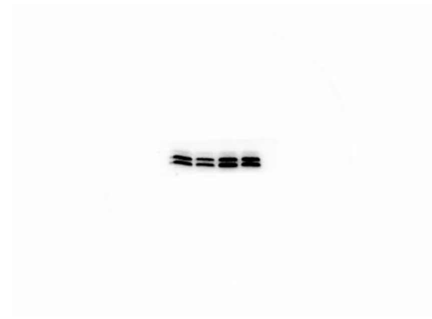
p-AKT	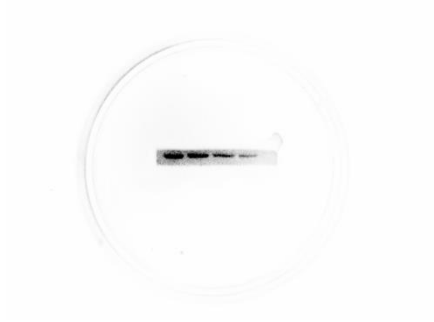		
β-actin	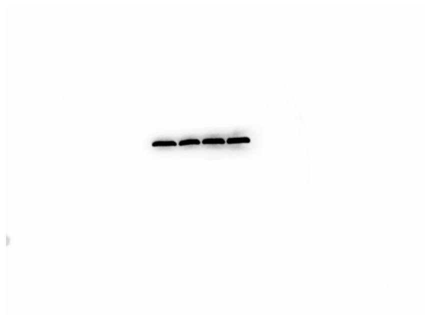	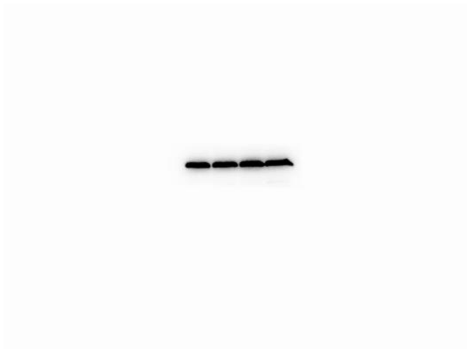	

Figure 6A (H226)			
mTOR	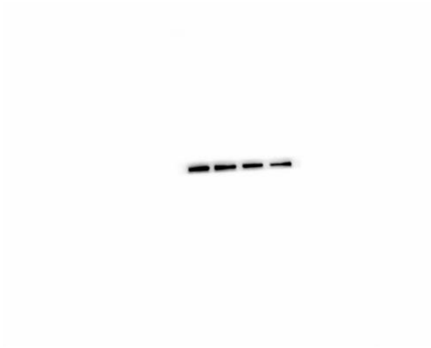		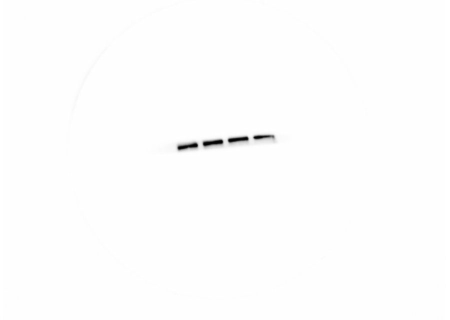
p-mTOR			
PI3K	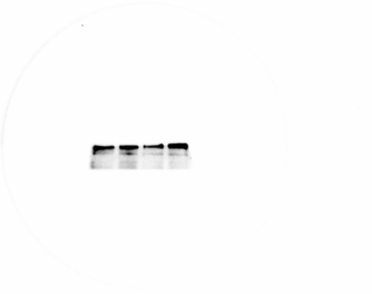	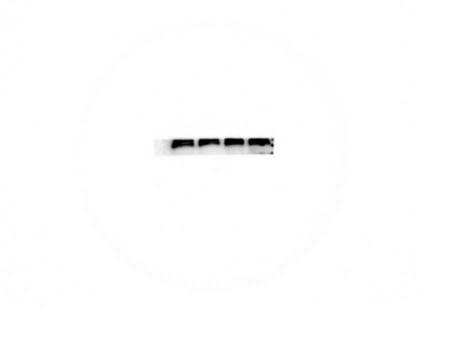	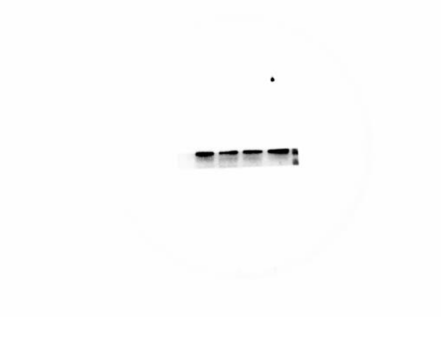
p-PI3K	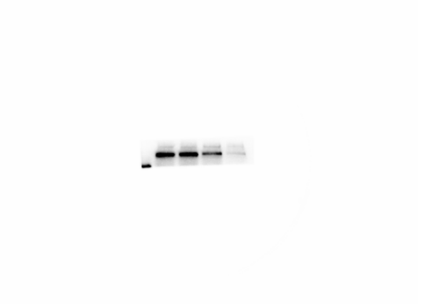	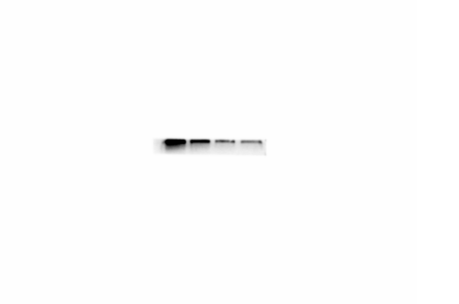	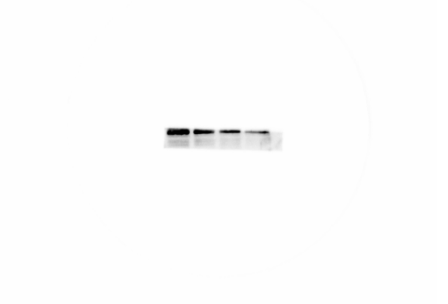

AKT	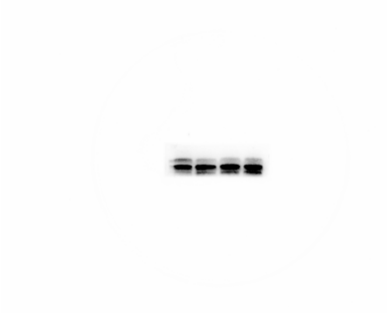	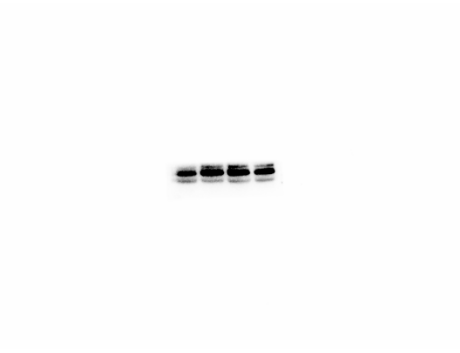	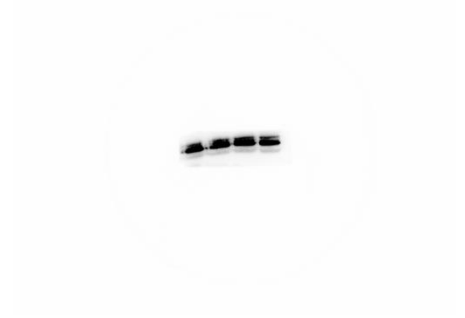
p-AKT		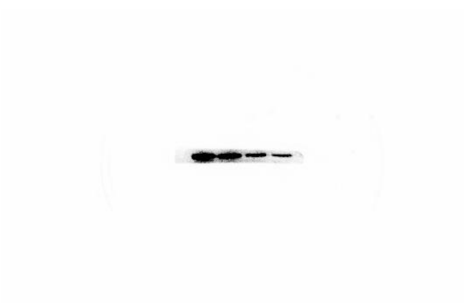	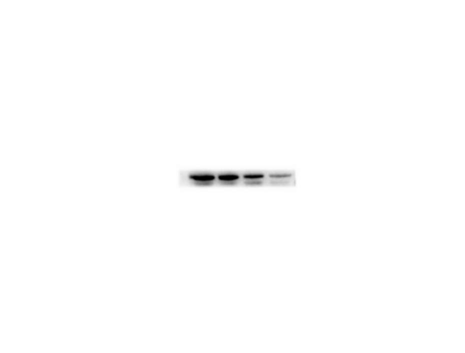
β-actin			

Figure 6C (A549)			
mTOR			
p-mTOR	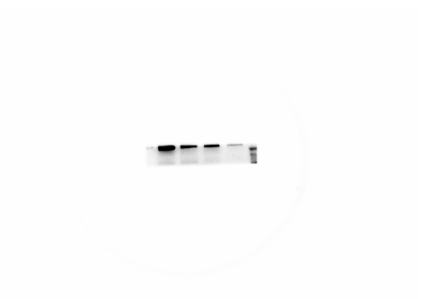		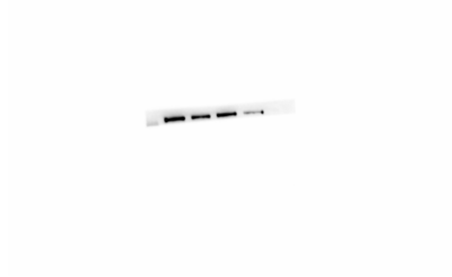
PI3K		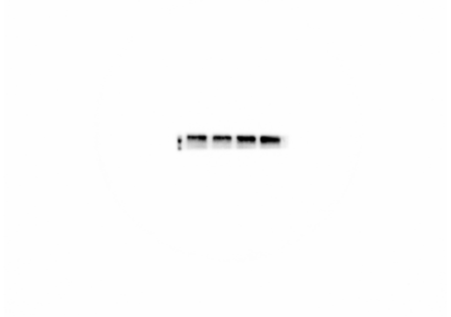	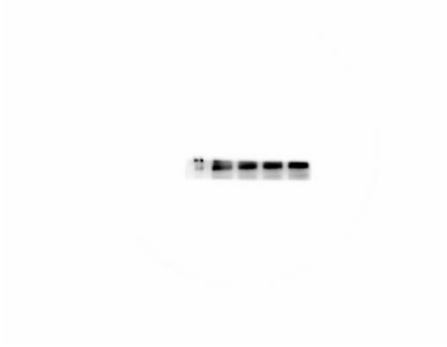
p-PI3K	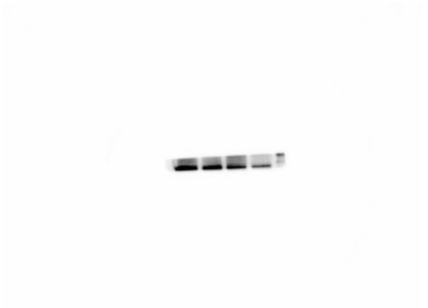	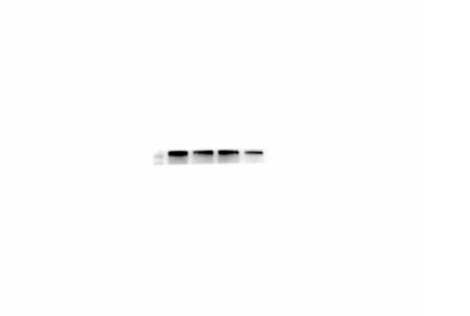	

AKT	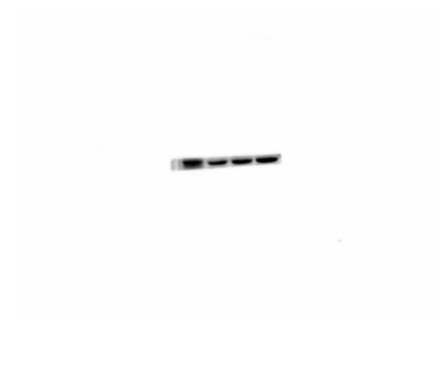	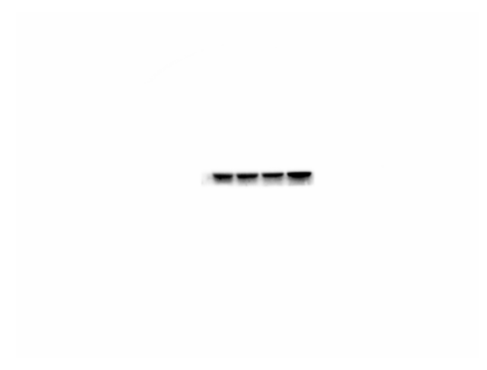	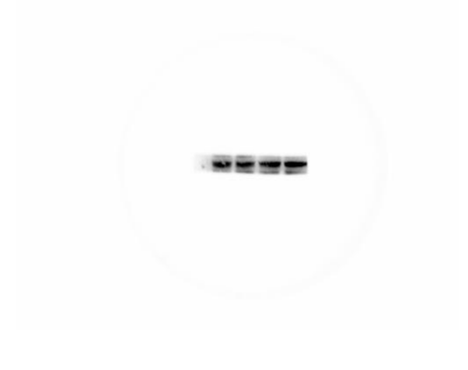
p-AKT			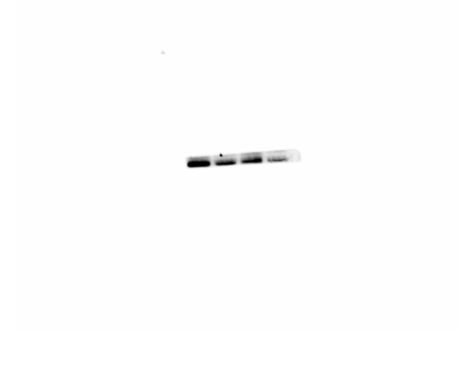
β-actin			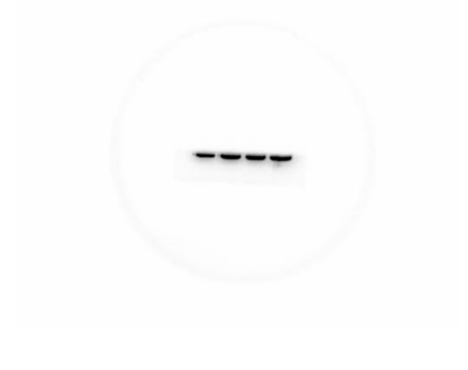

Figure 6C (H1299)			
mTOR			
p-mTOR			
PI3K		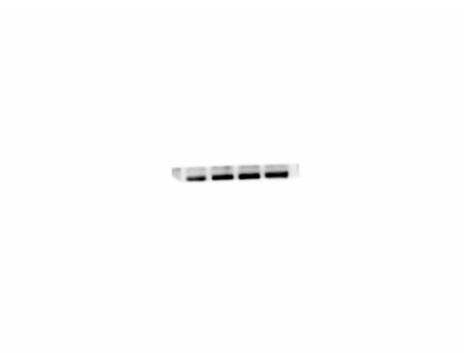	
p-PI3K	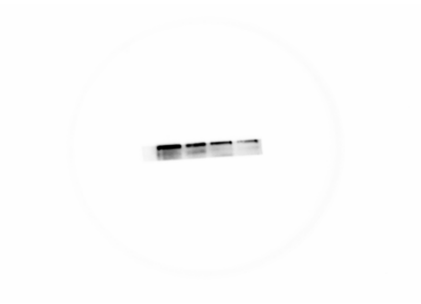	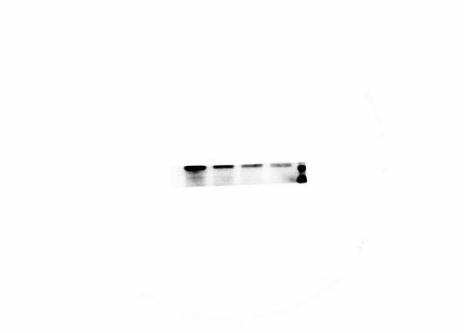	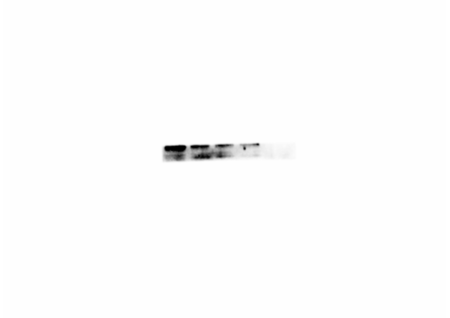

AKT			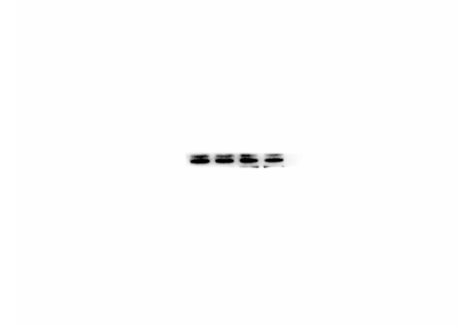
p-AKT	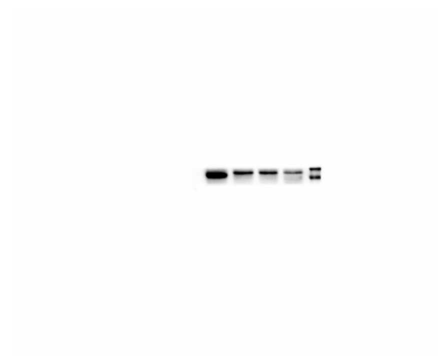	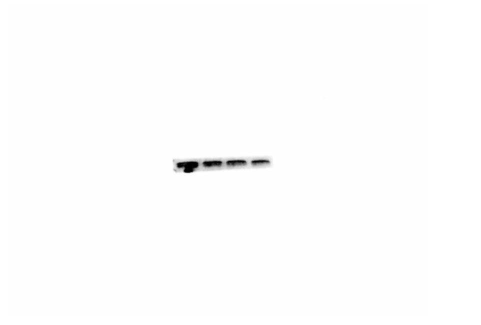	
β-actin	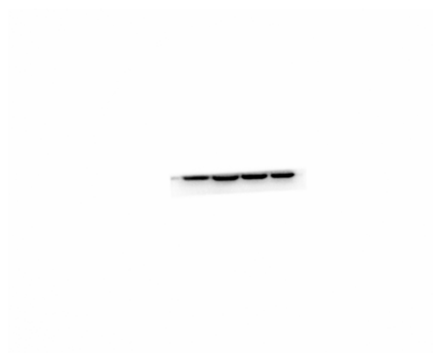		

Figure 6C (H226)			
mTOR			
p-mTOR			
PI3K	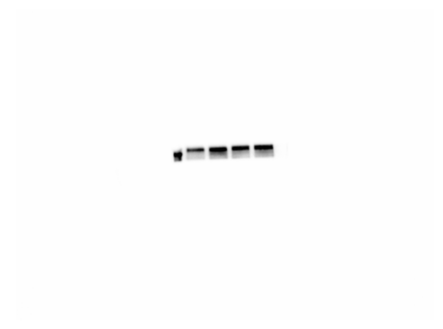		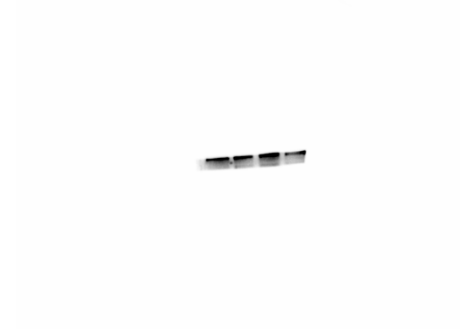
p-PI3K	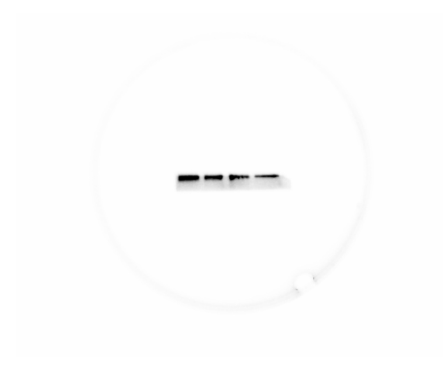	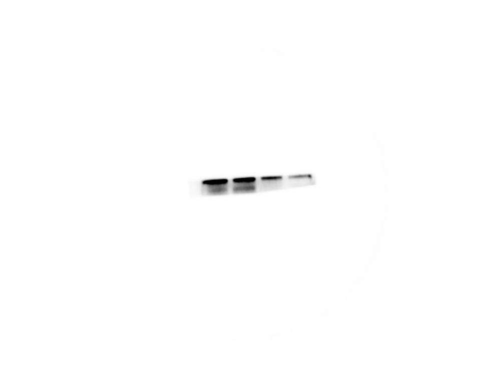	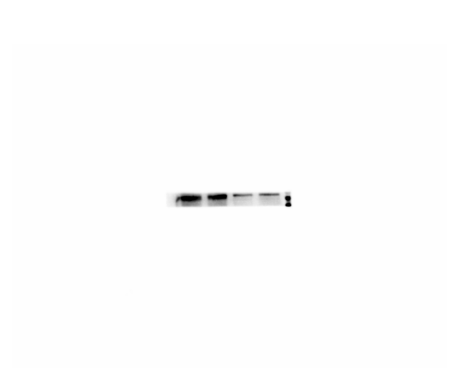

AKT			
p-AKT	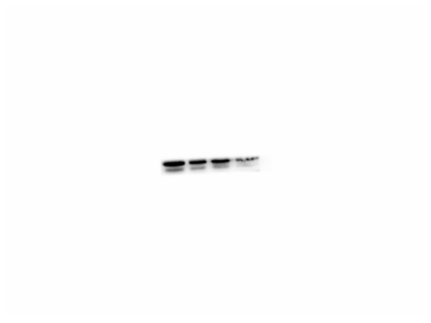	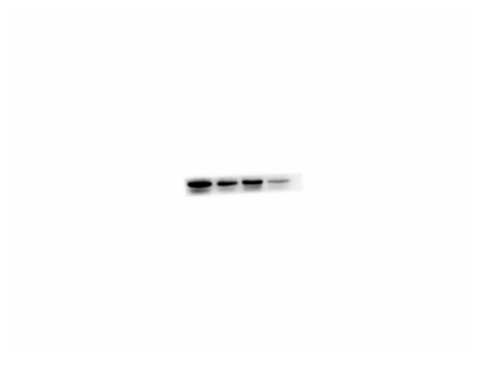	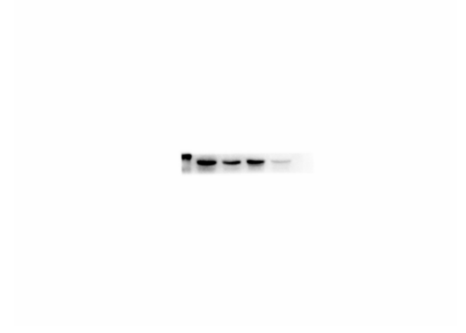
β-actin	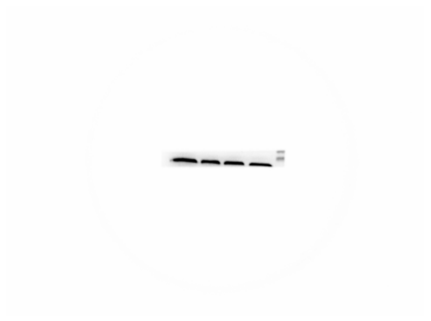		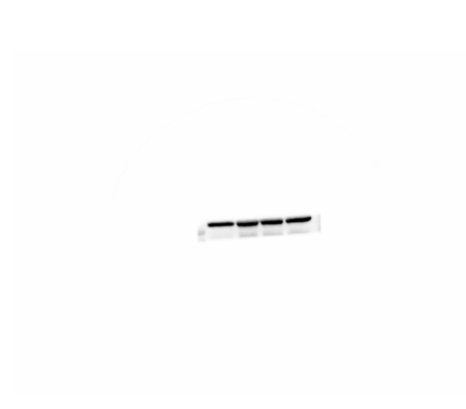

Figure 6E (A549)			
Bcl2		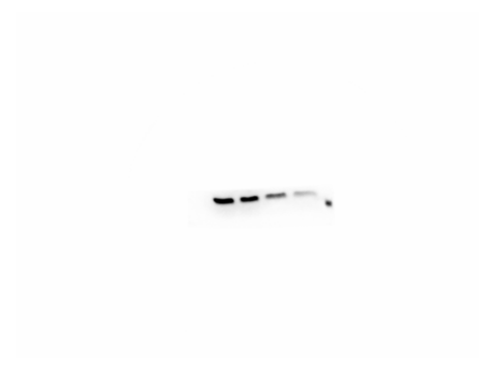	
LC3	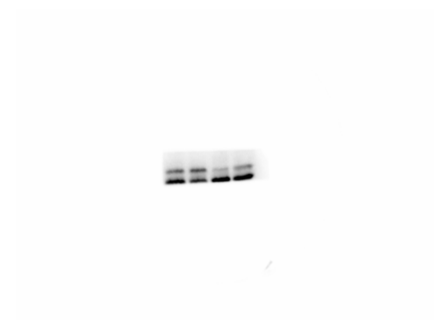	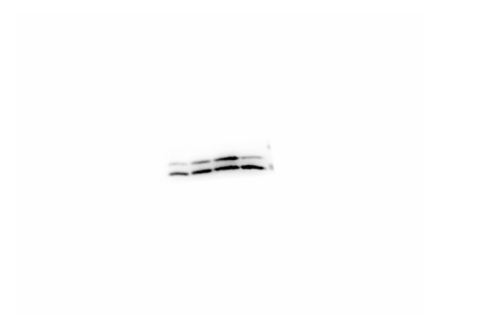	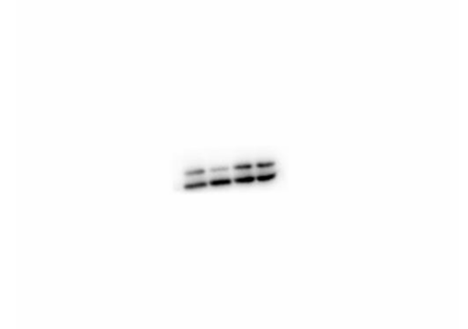
β-actin			

Figure 6E (H1299)			
Bcl2		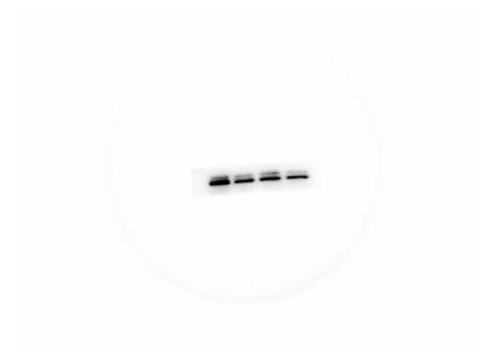	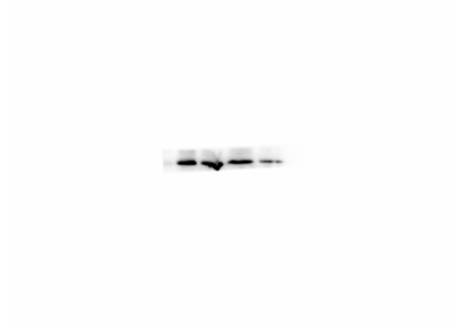
LC3	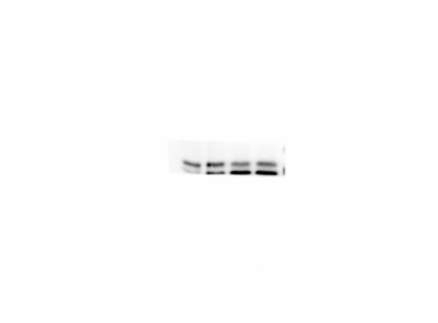	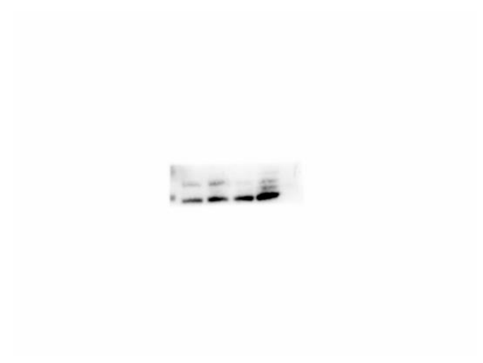	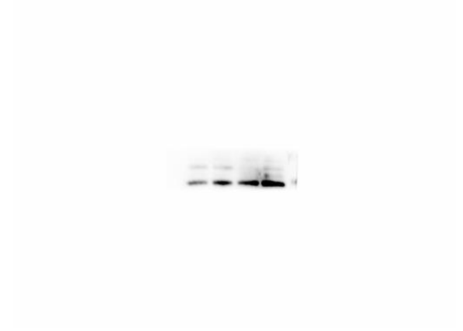
β-actin			

Figure 6E (H226)			
Bcl2	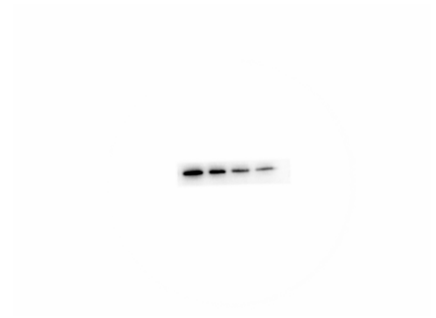		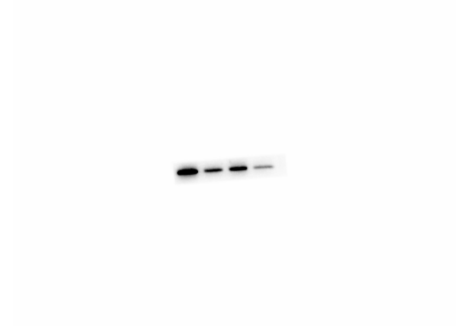
LC3	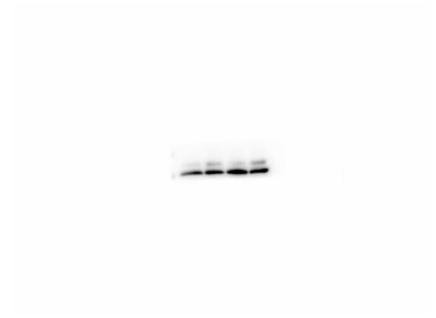	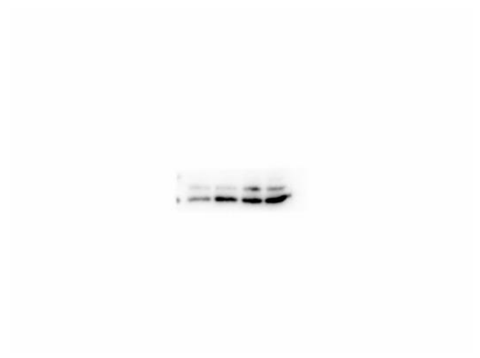	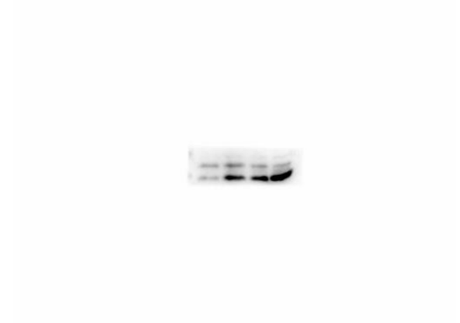
β-actin			

Figure 7D (A549)			
Bcl2	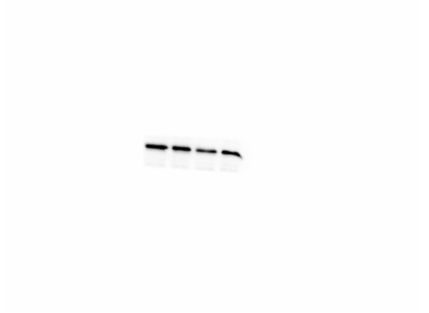	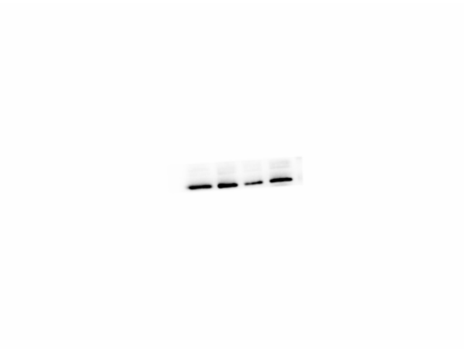	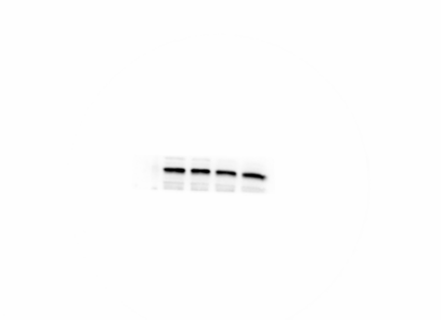
LC3	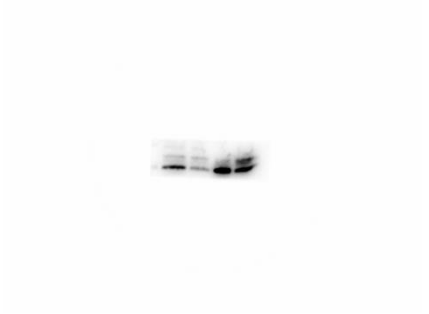	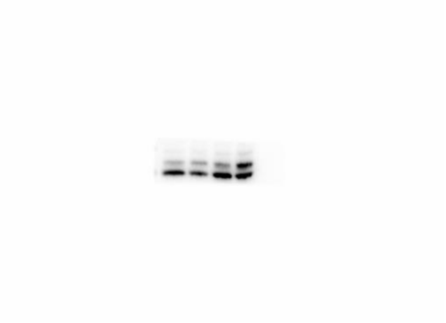	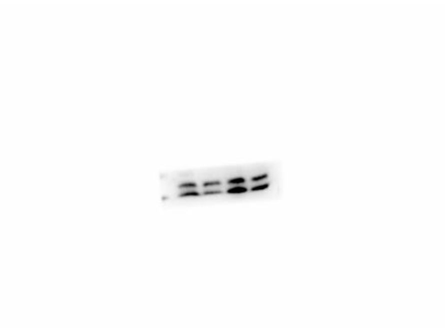
β-actin	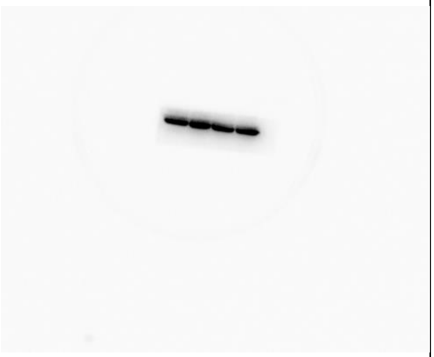	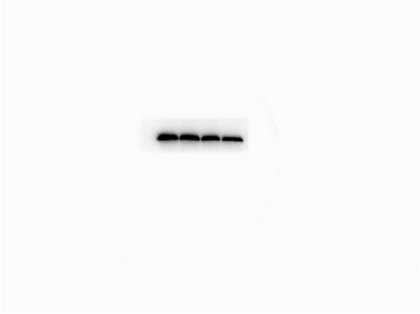	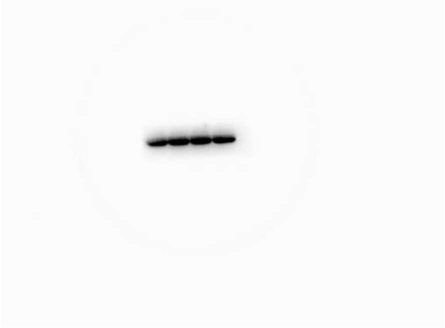

Figure 7D (H1299)			
Bcl2	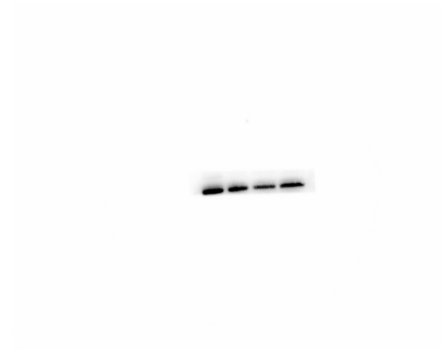		
LC3		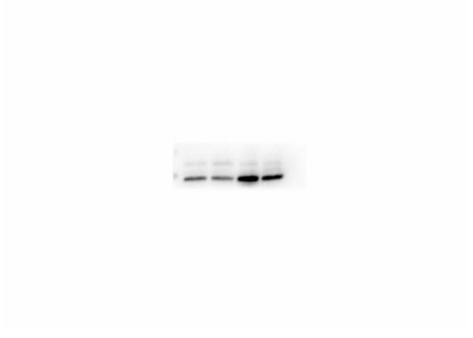	
β-actin	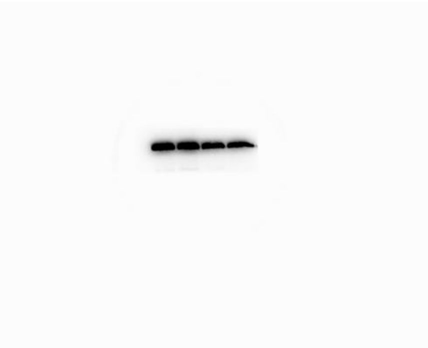	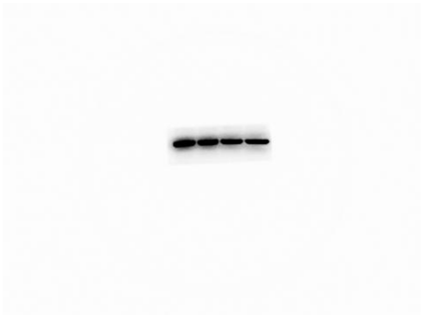	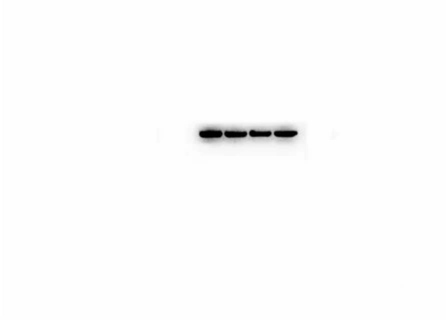

Figure 7D (H226)			
Bcl2		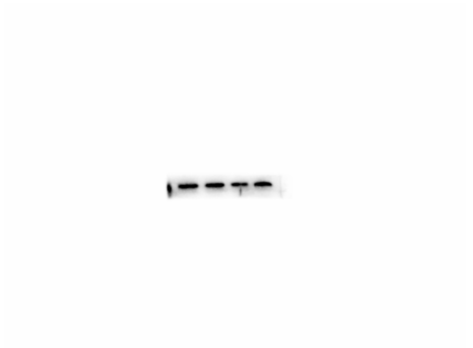	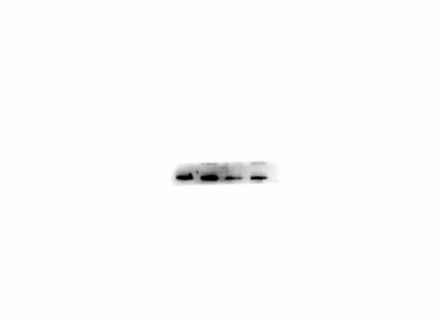
LC3	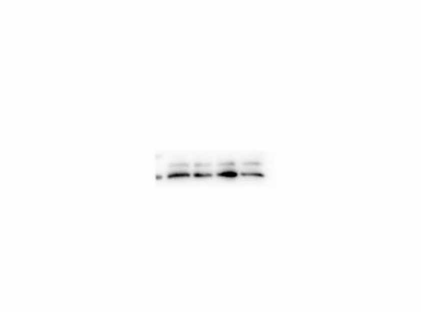	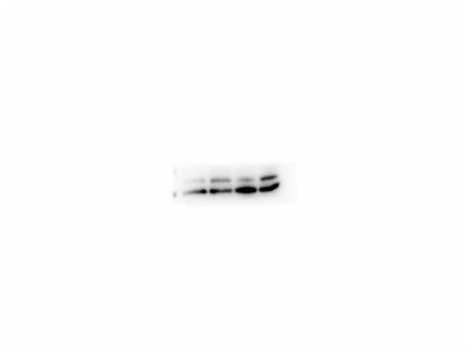	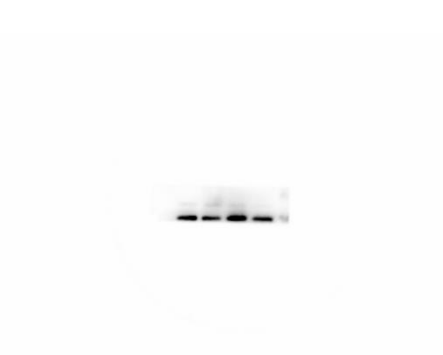
β-actin	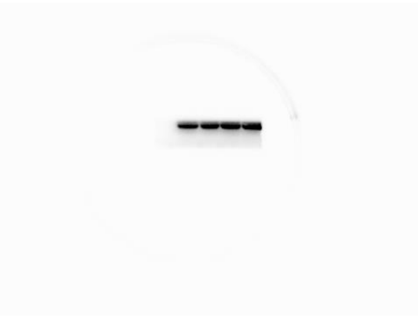	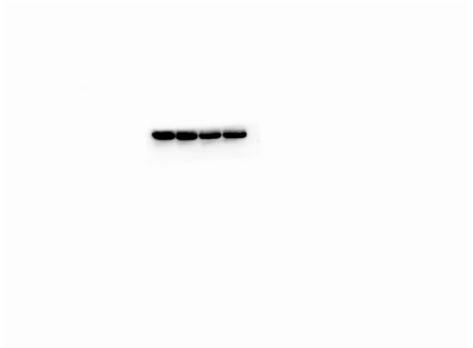	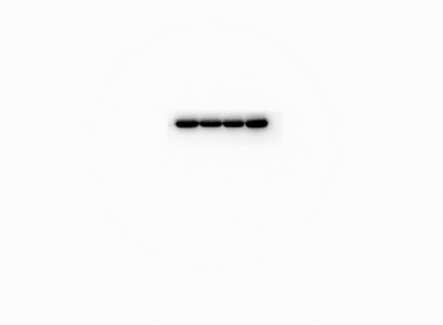

Figure 7F (A549)			
mTOR			
p-mTOR	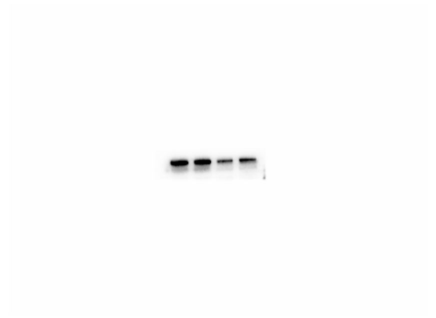	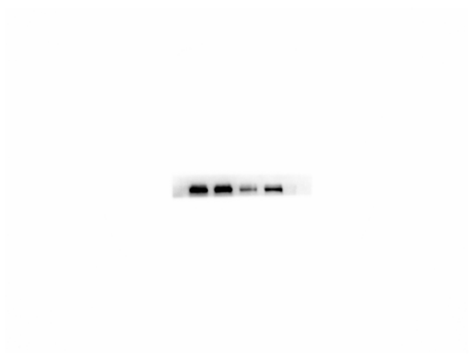	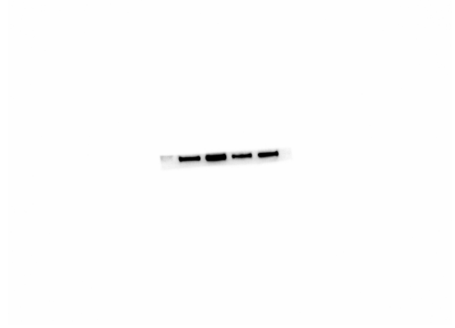
PI3K	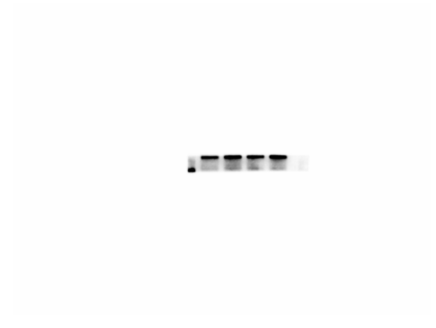	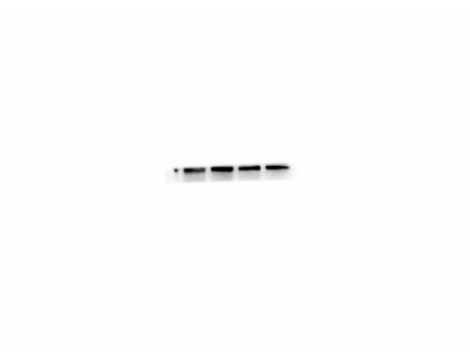	
p-PI3K	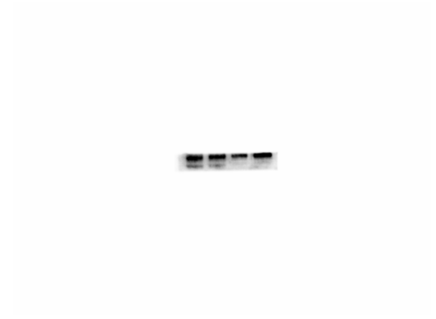	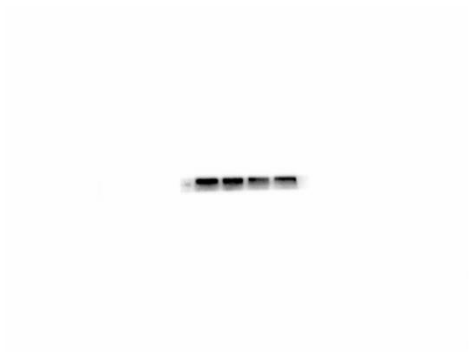	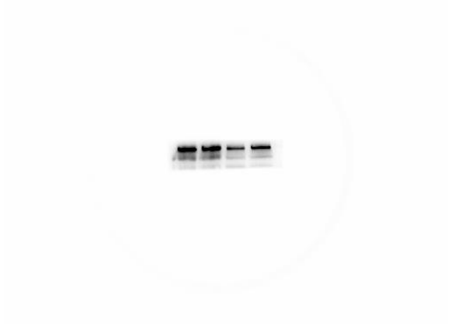

AKT	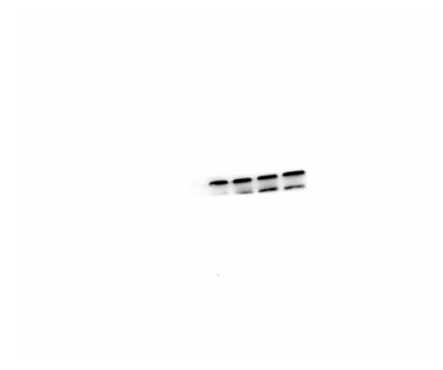	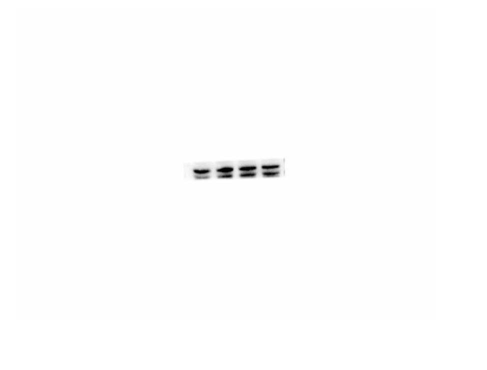	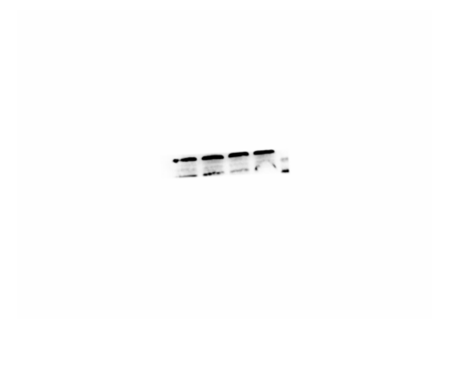
p-AKT	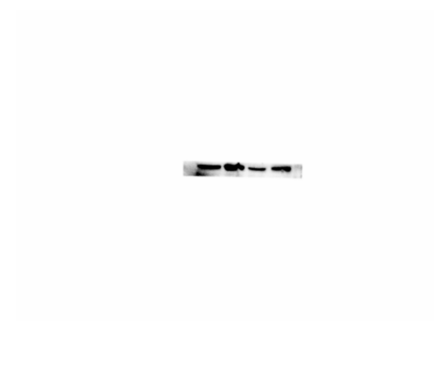	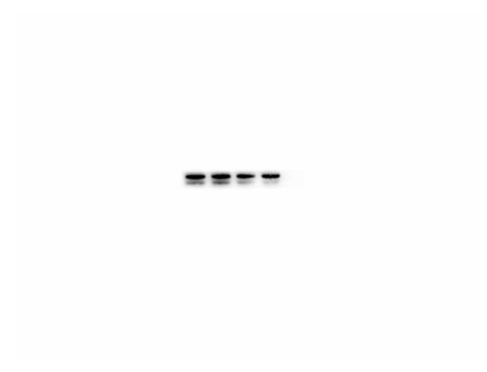	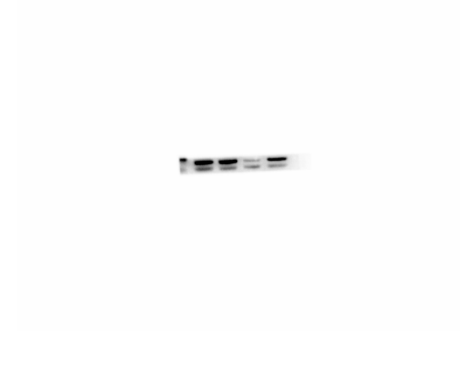
β-actin	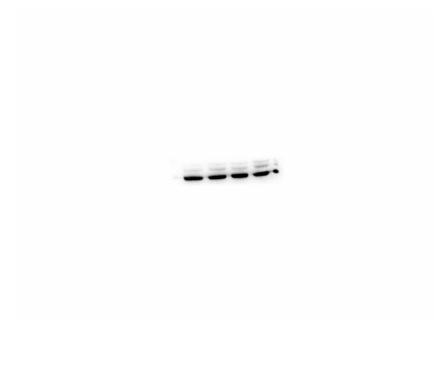	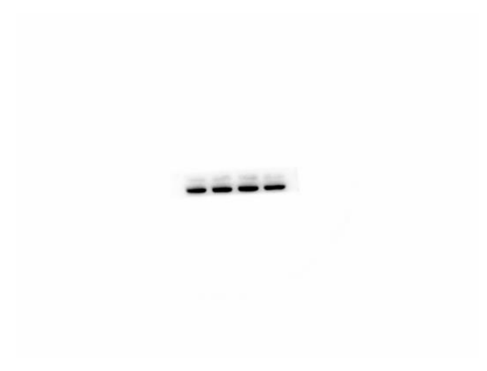	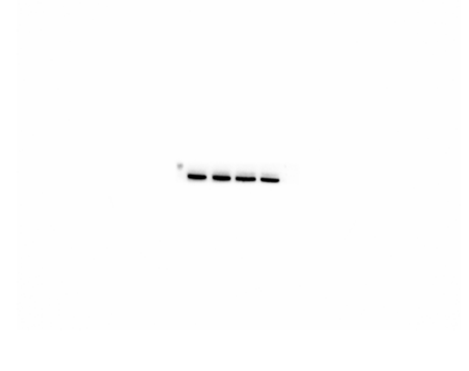

Figure 7F (H1299)			
mTOR			
p-mTOR			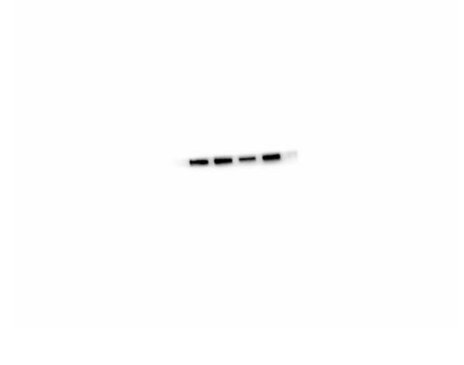
PI3K	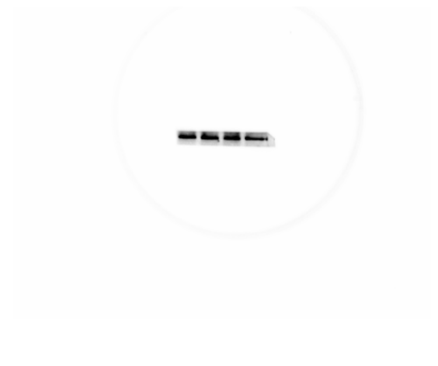	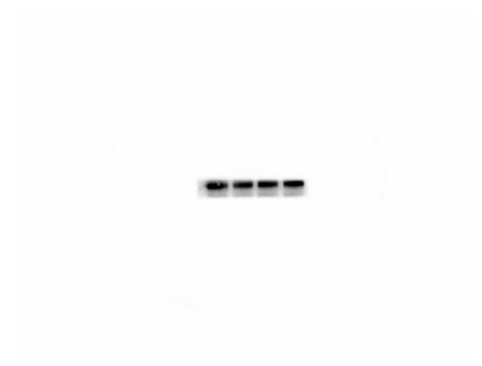	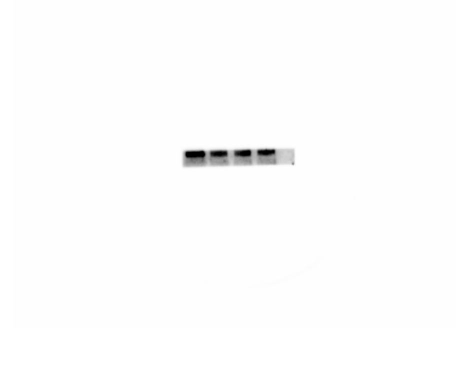
p-PI3K	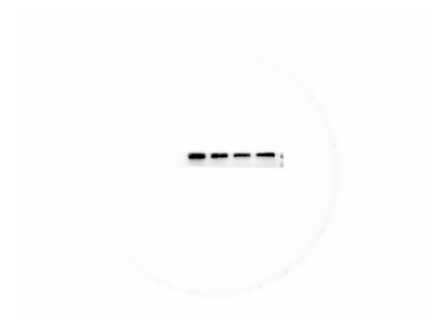		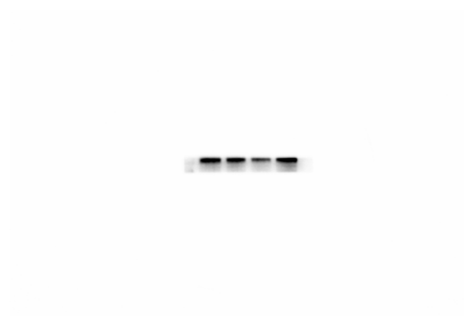

AKT	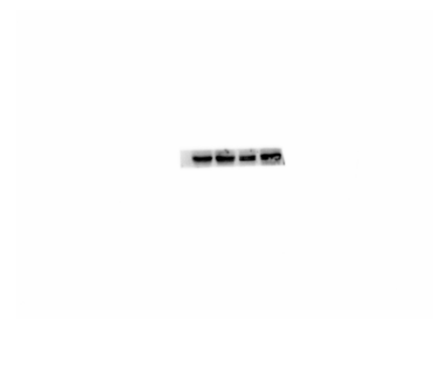	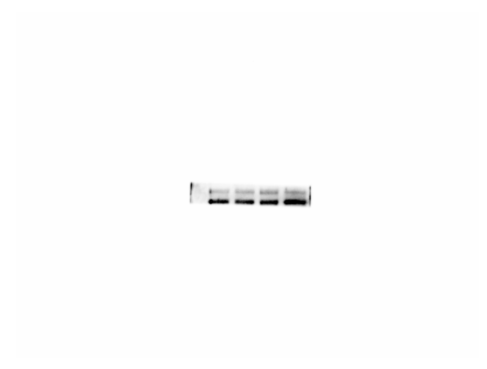	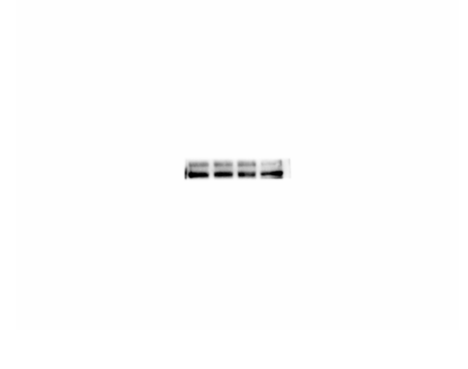
p-AKT	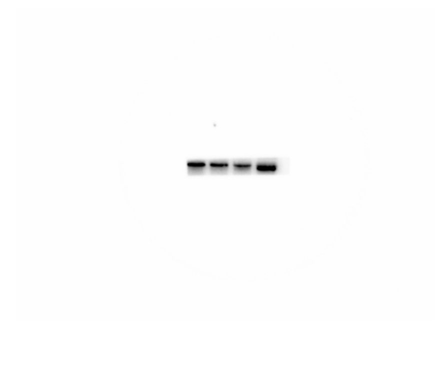		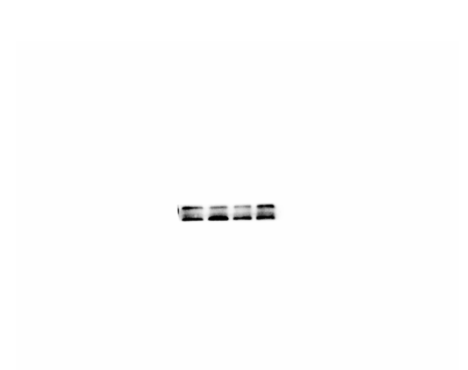
β-actin	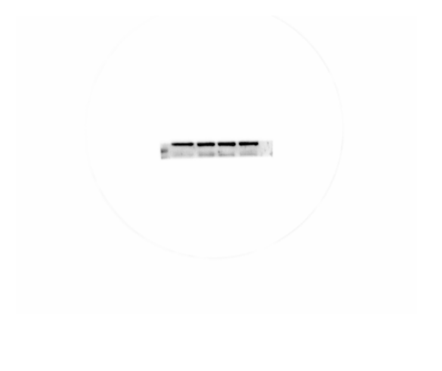	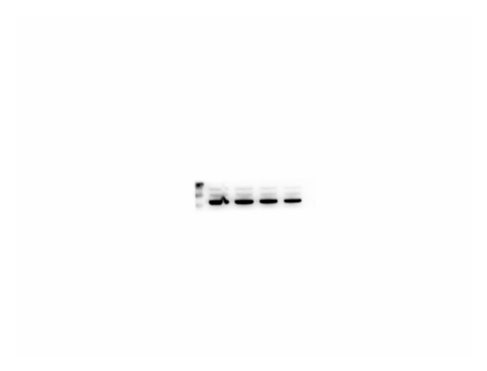	

Figure 7F (H226)			
mTOR	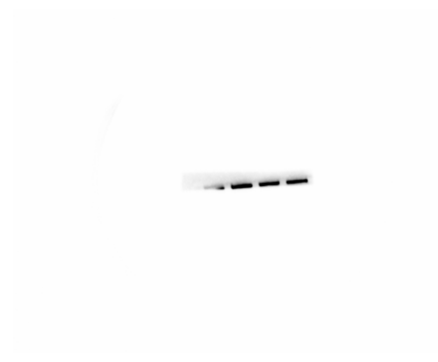		
P-mTOR			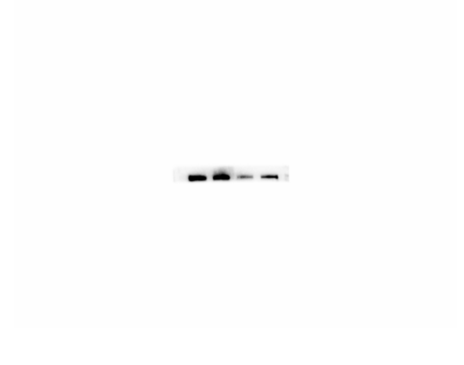
PI3K	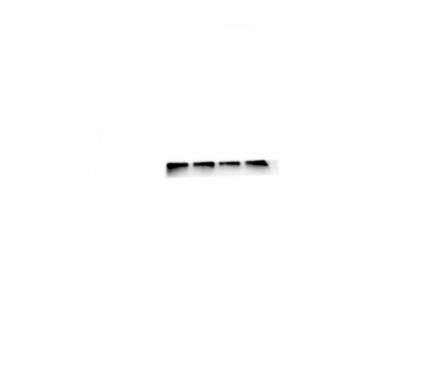	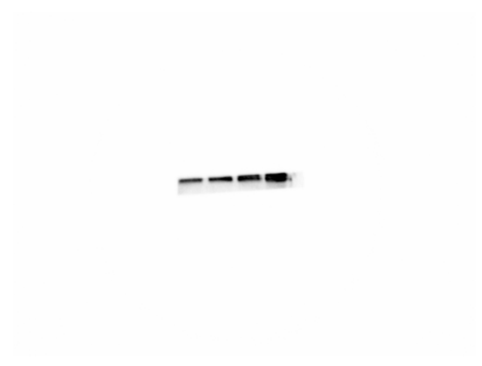	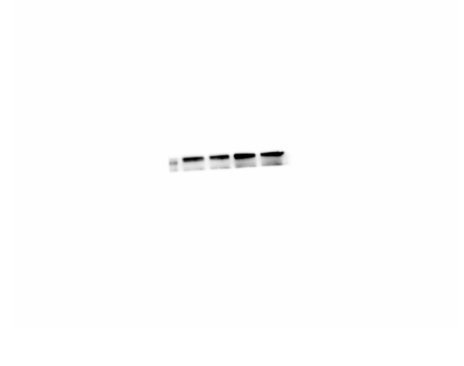
p-PI3K	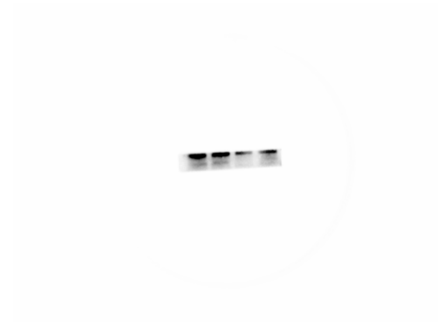	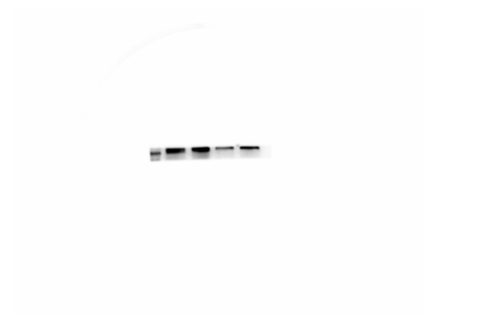	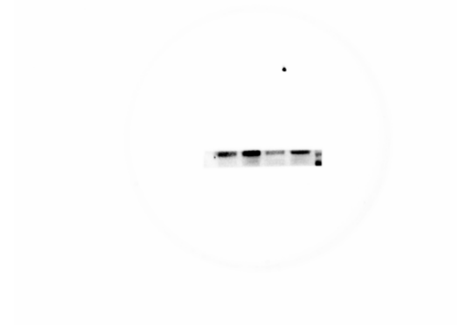

AKT	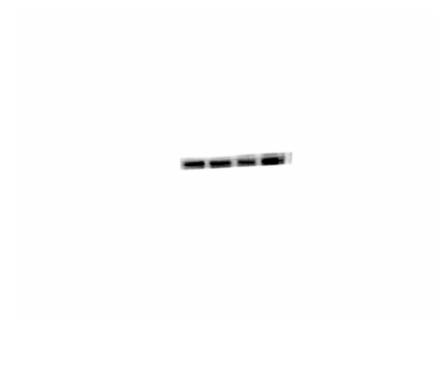	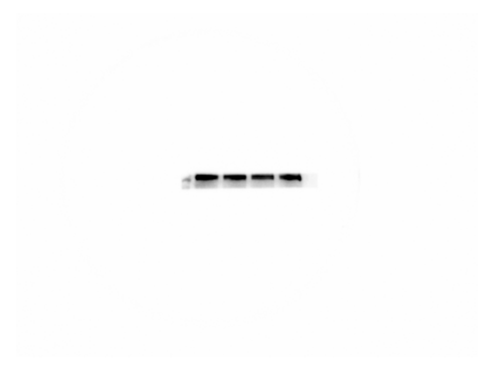	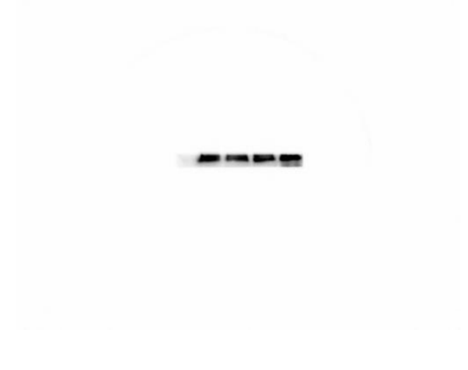
p-AKT	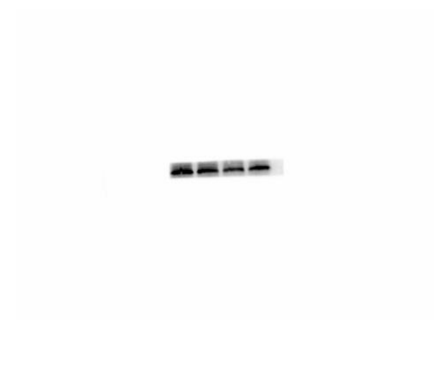	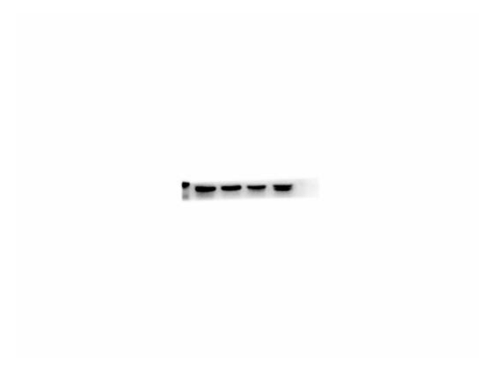	
β-actin	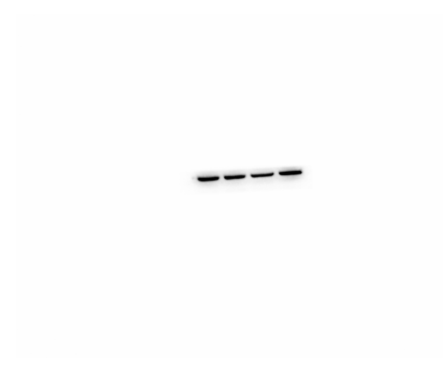		

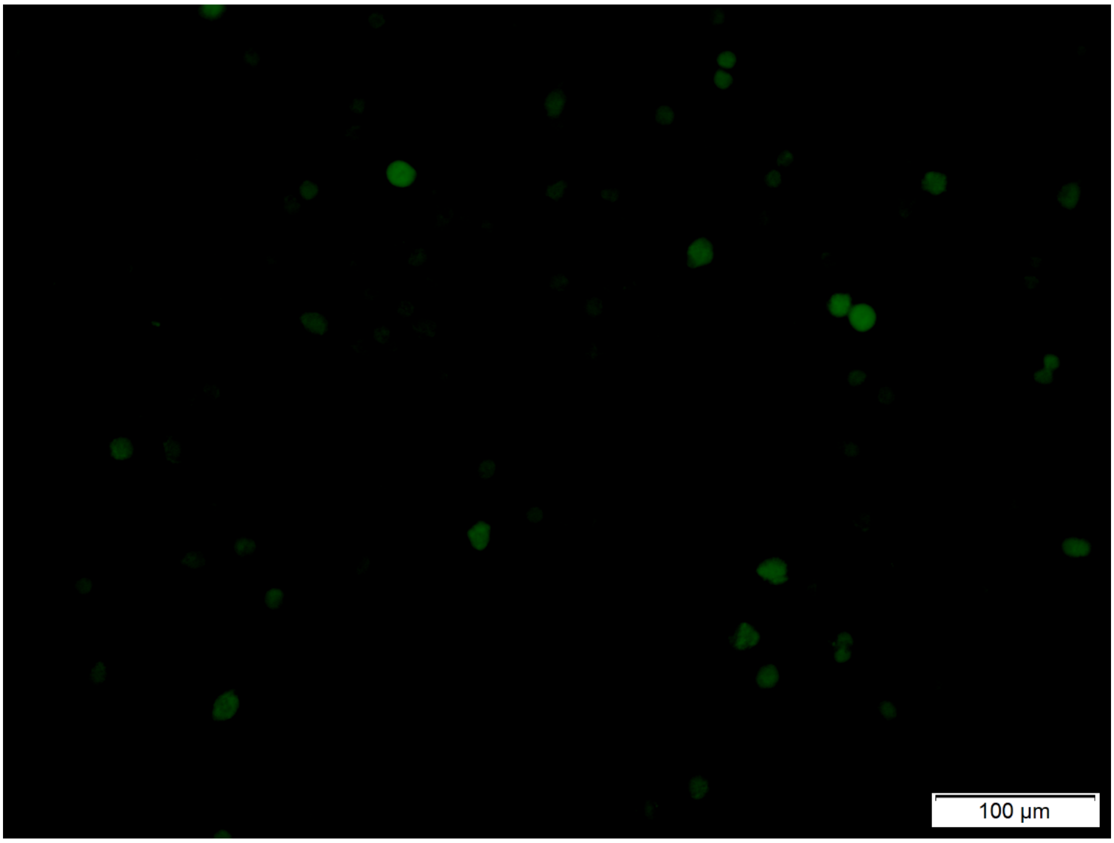

Fig. 7A_A549_control

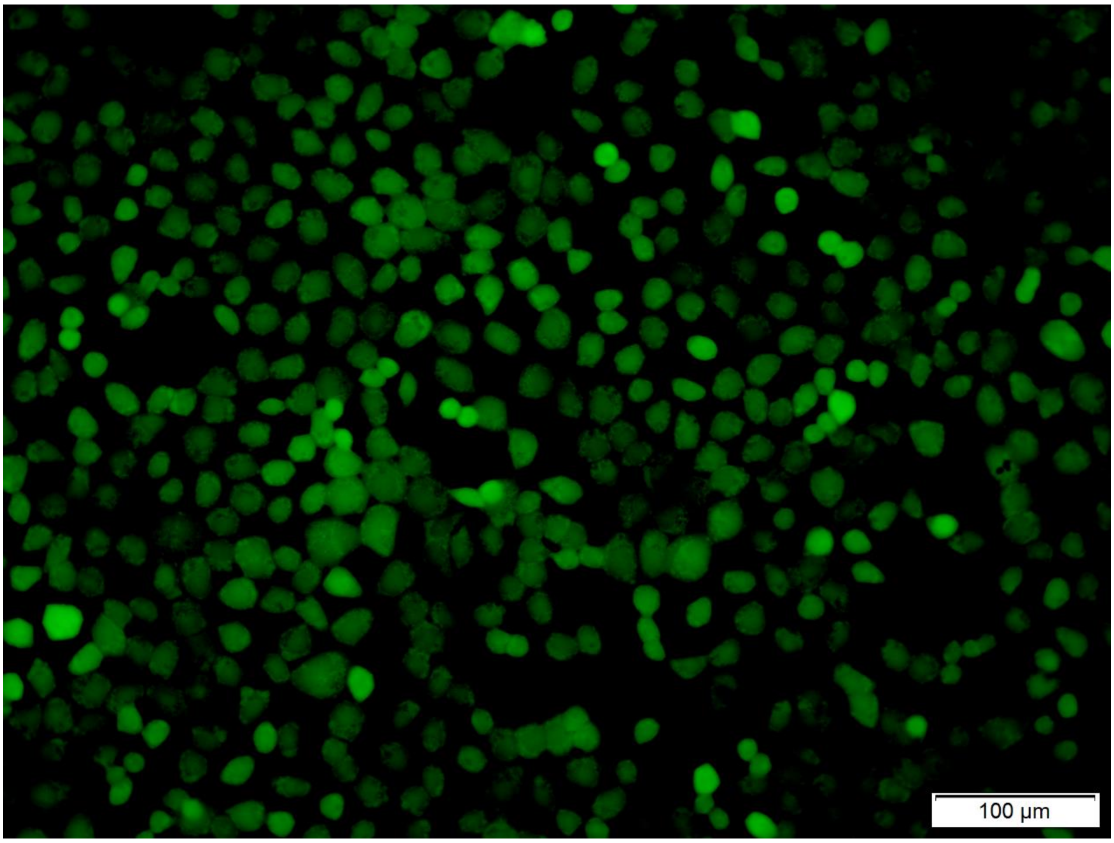

Fig. 7A_A549_ABX_10µM

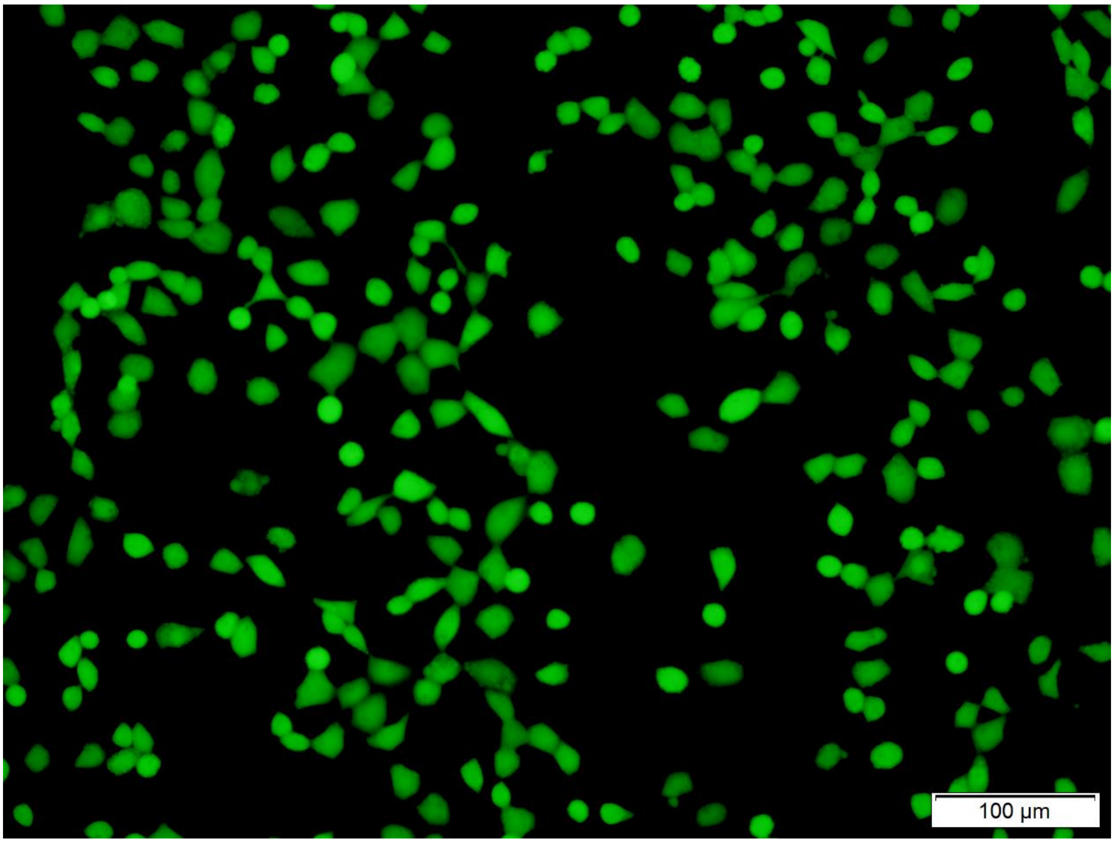

Fig. 7A_A549_ABX_20µM

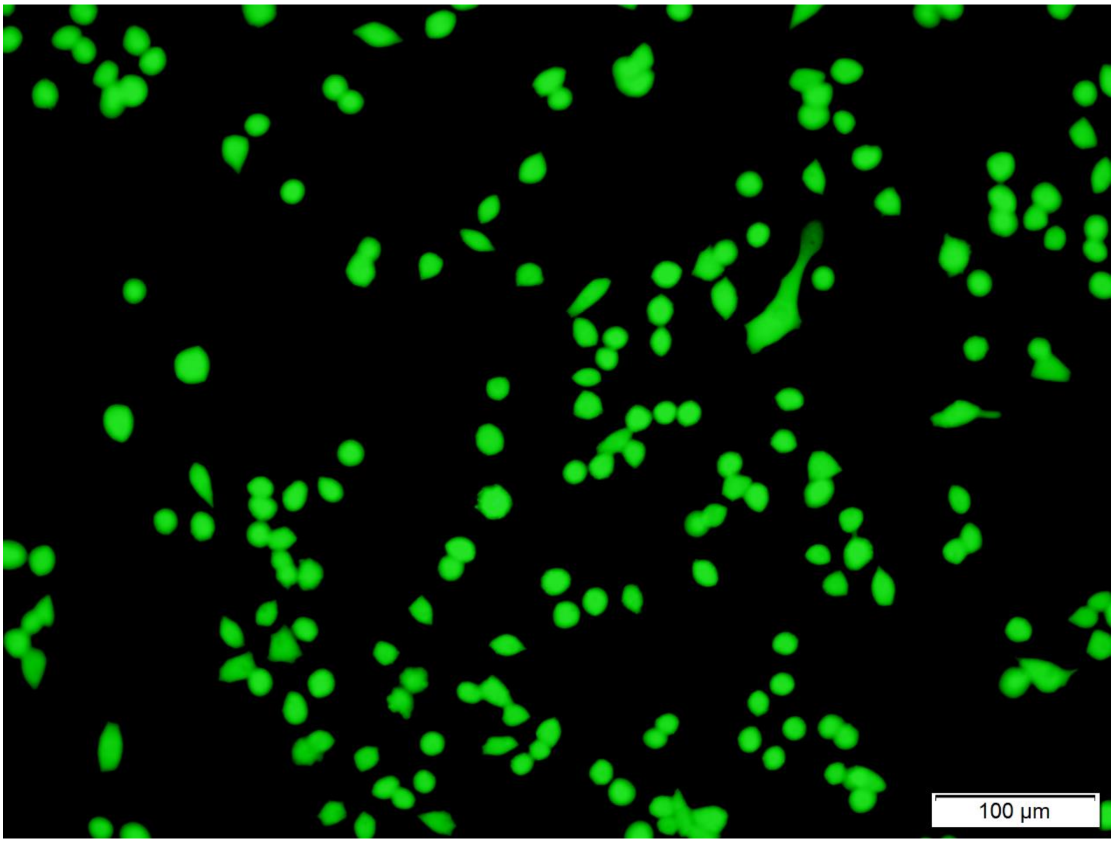

Fig. 7A_A549_ABX_40µM

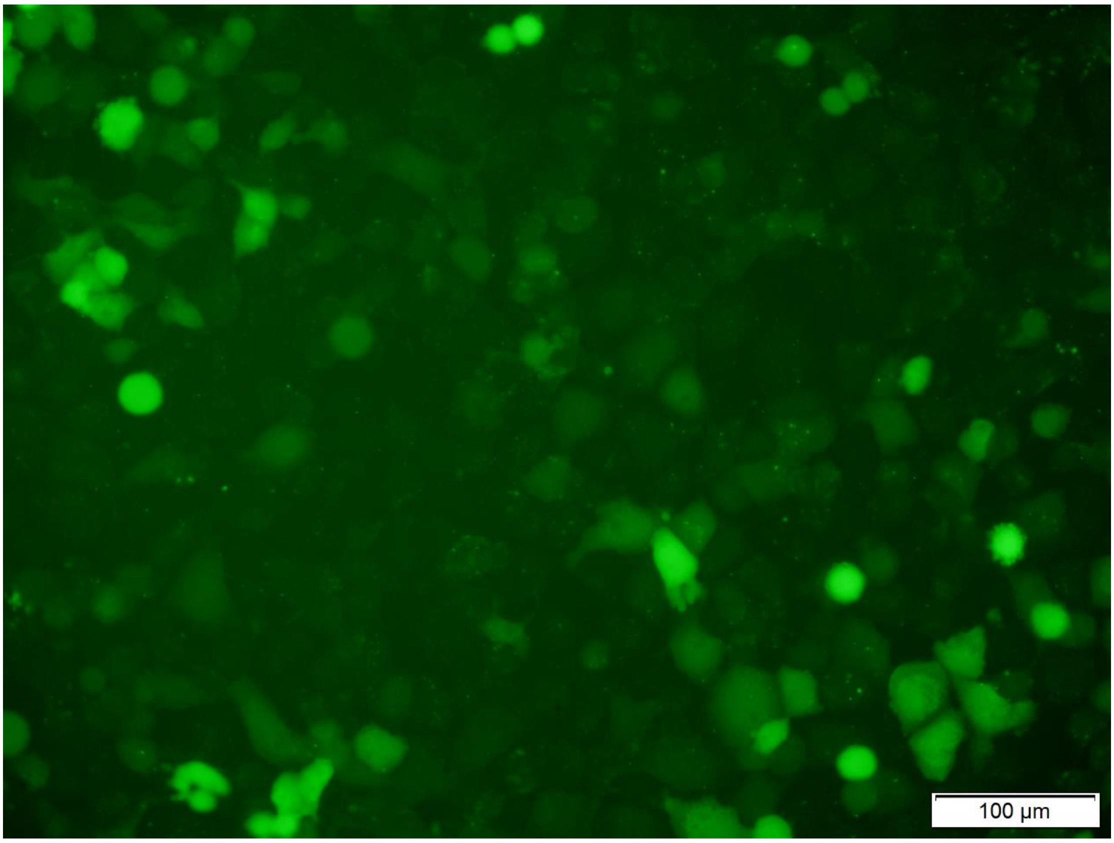

Fig. 7A_H1299_control

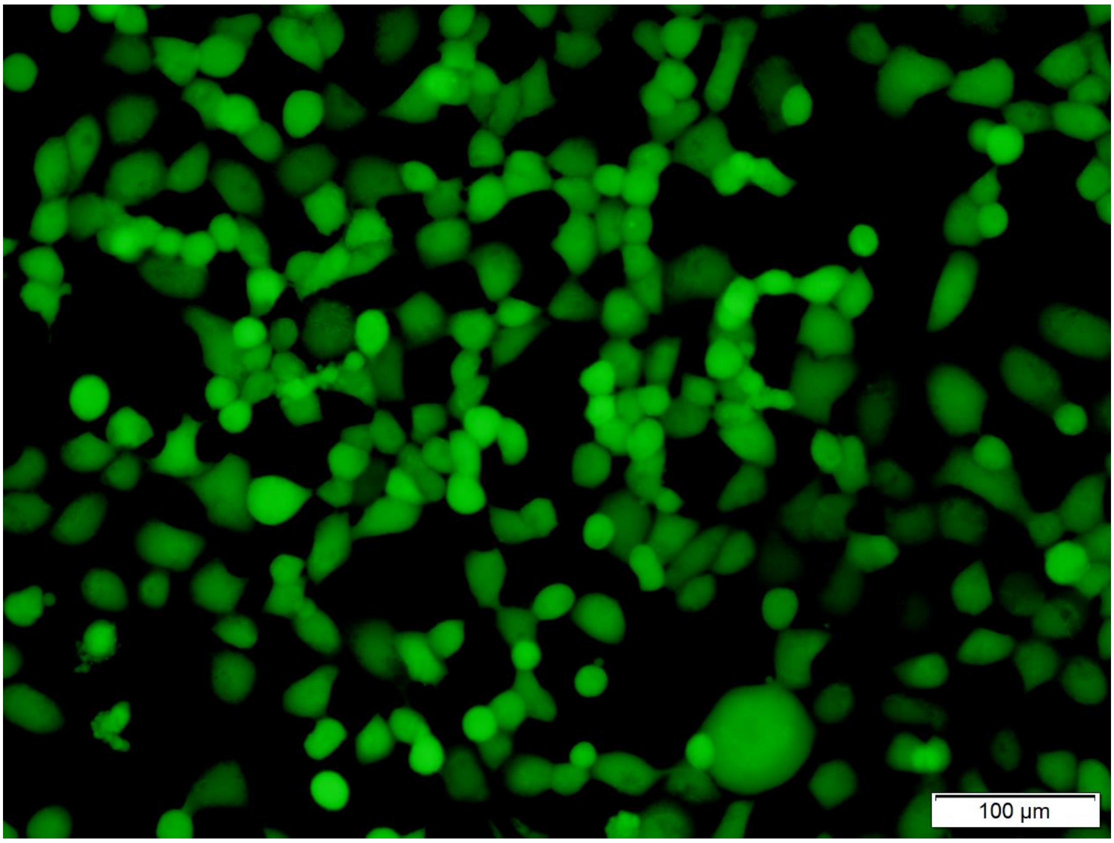

Fig. 7A_H1299_ABX_10µM

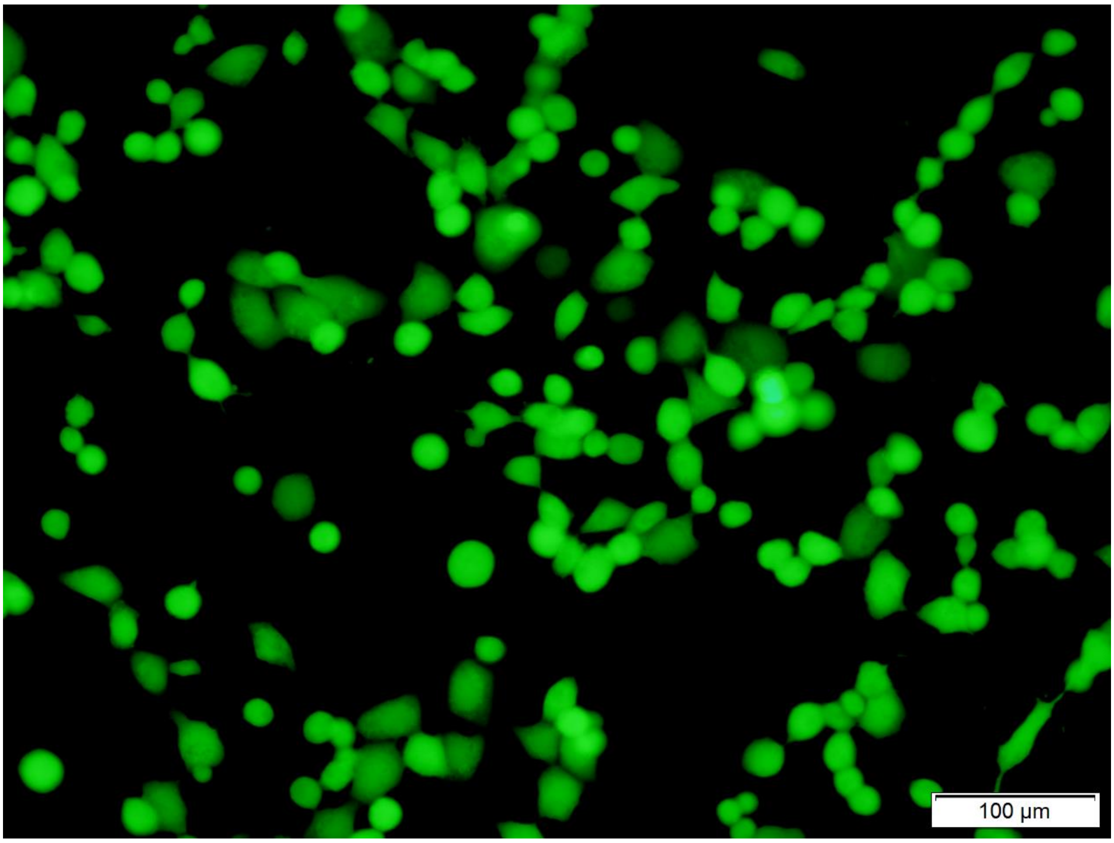

Fig. 7A_H1299_ABX_20µM

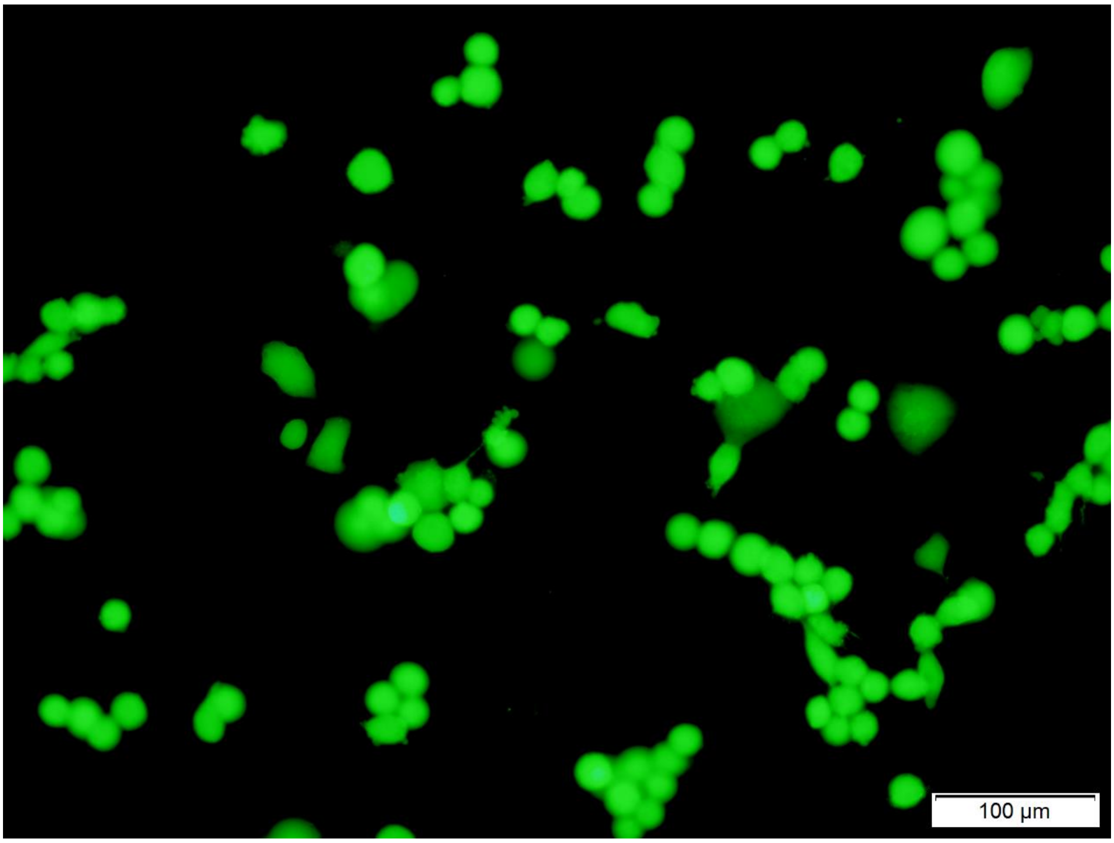

Fig. 7A_H1299_ABX_40µM

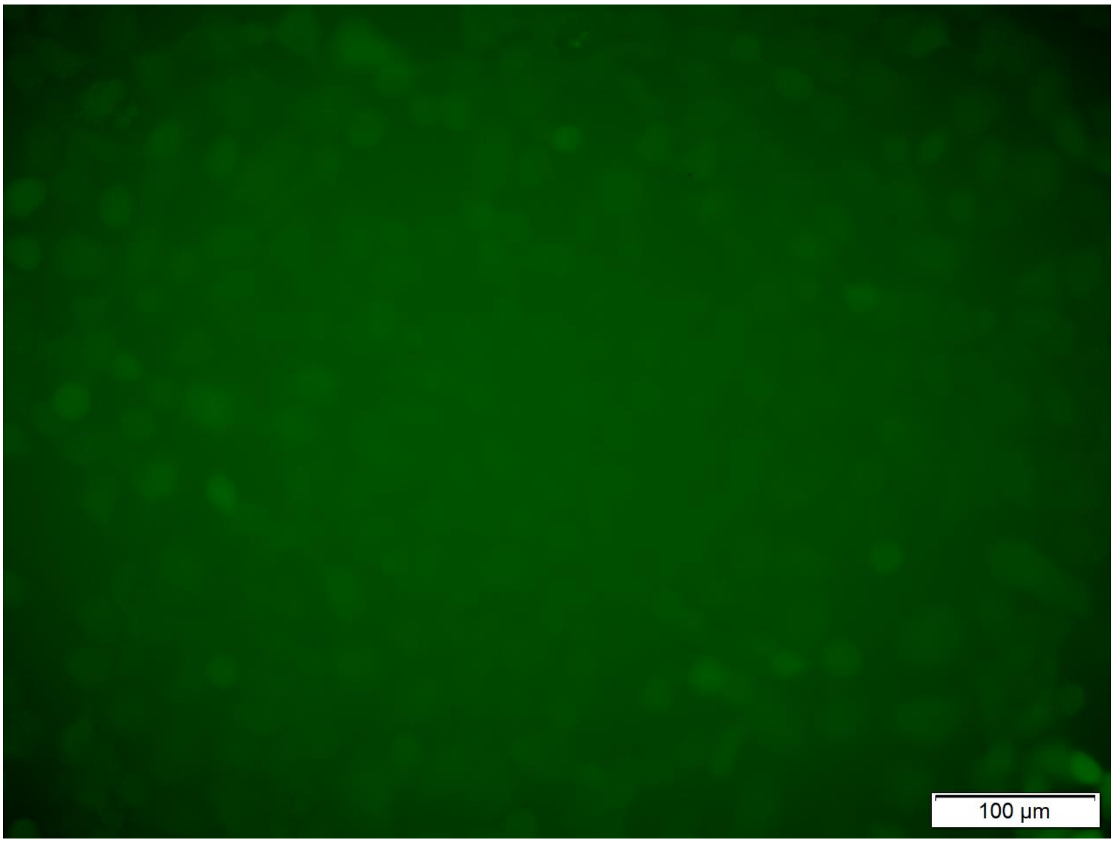

Fig. 7A_H226_control

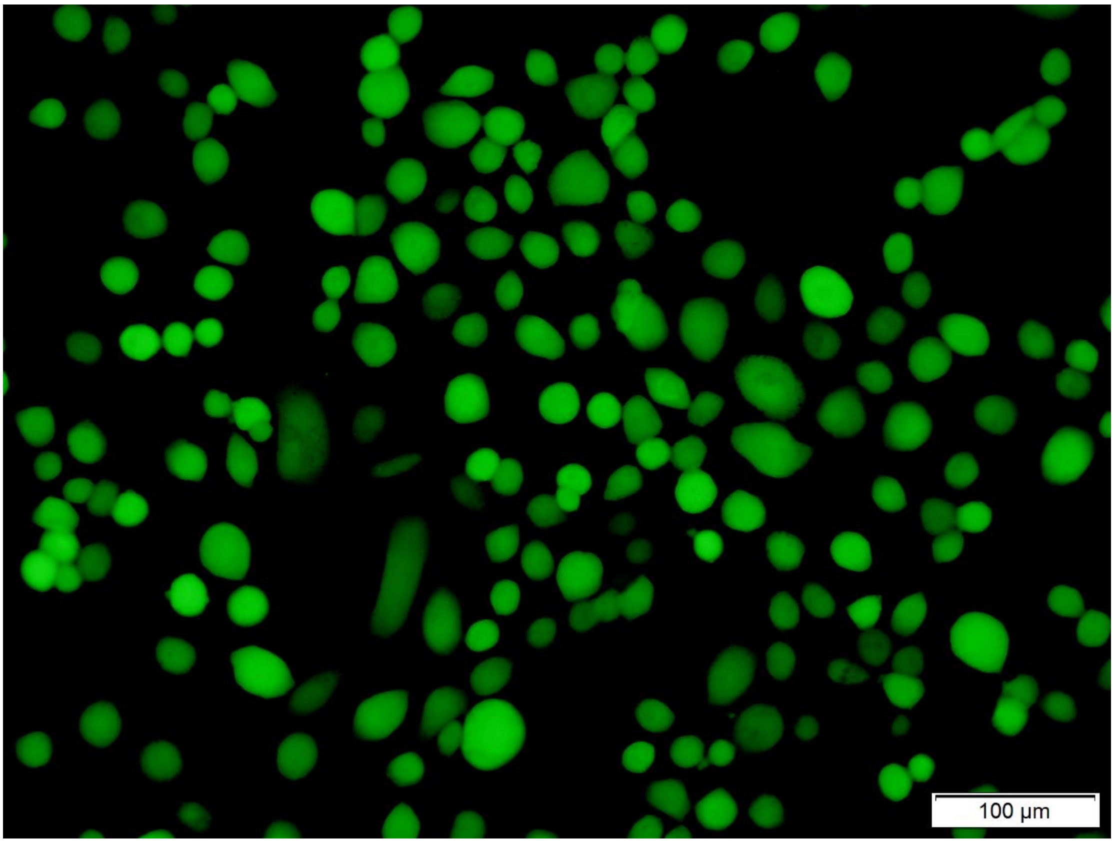

Fig. 7A_H226_ABX_10µM

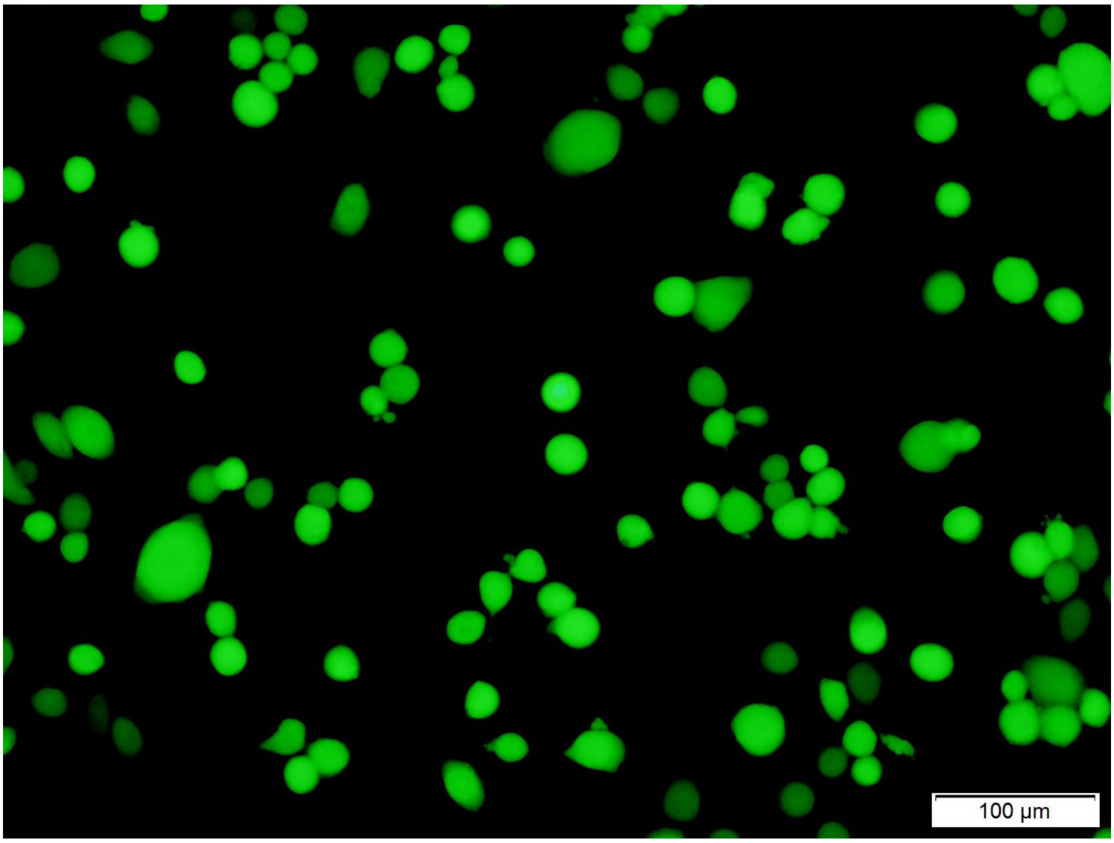

Fig. 7A_H226_ABX_20µM

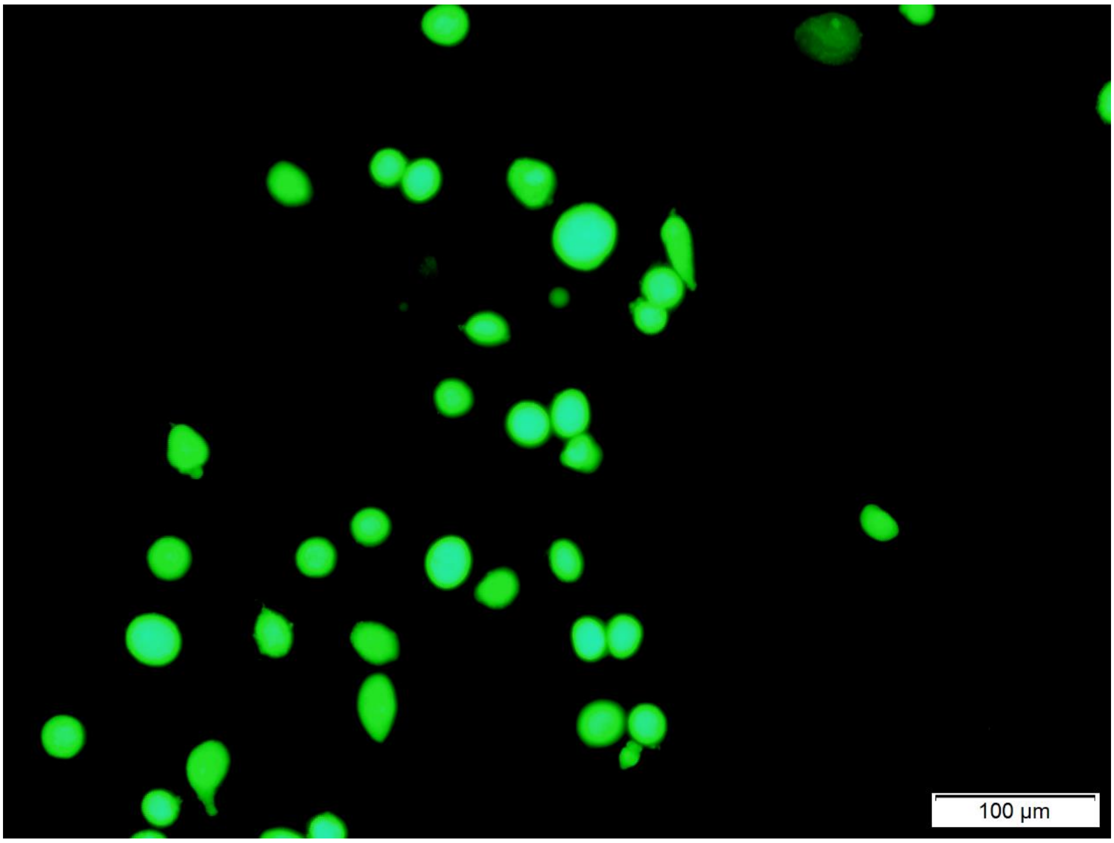

Fig. 7A_H226_ABX_40µM

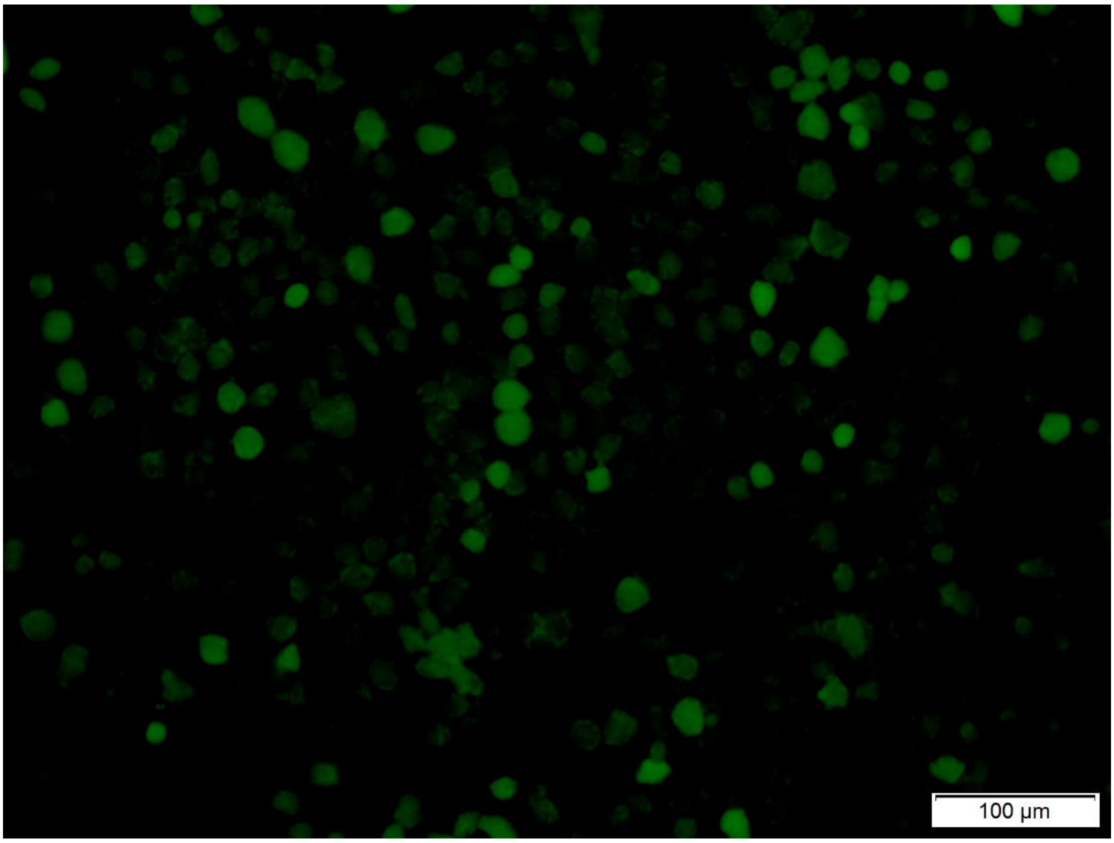

Fig. 7B_A549_control

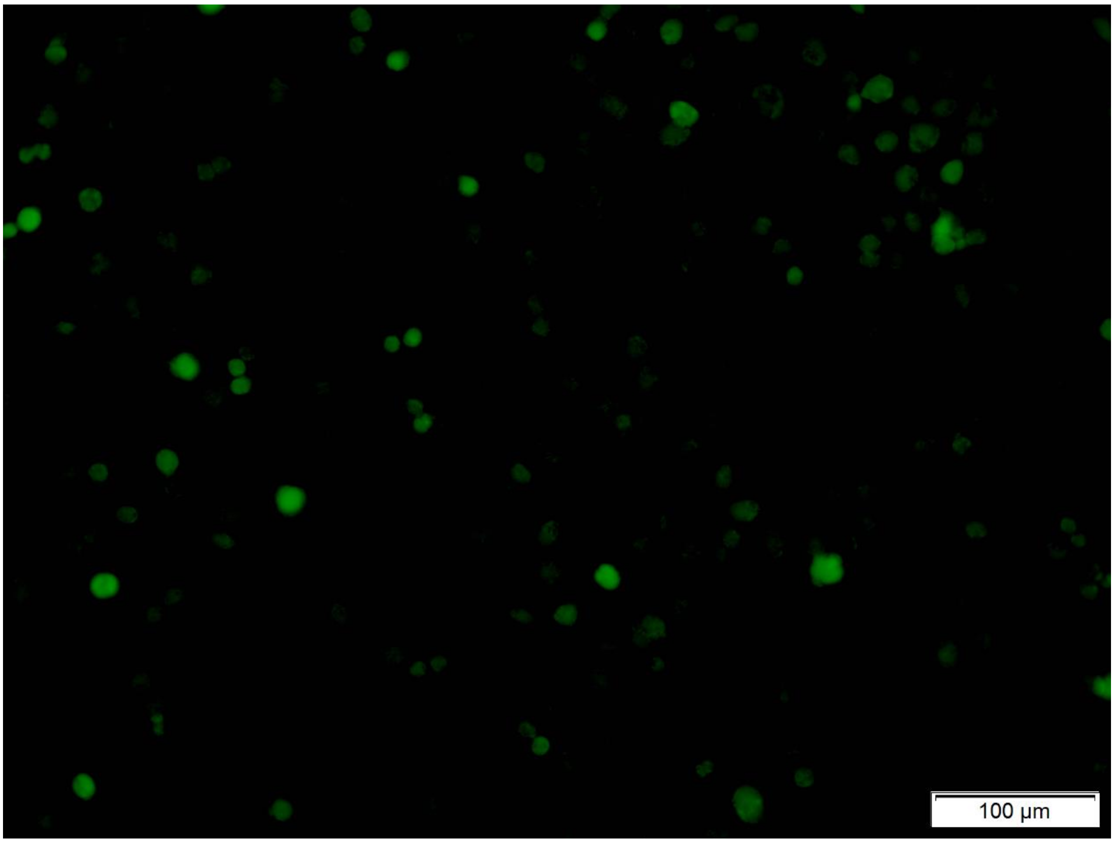

Fig. 7B_A549_NAC_10µM

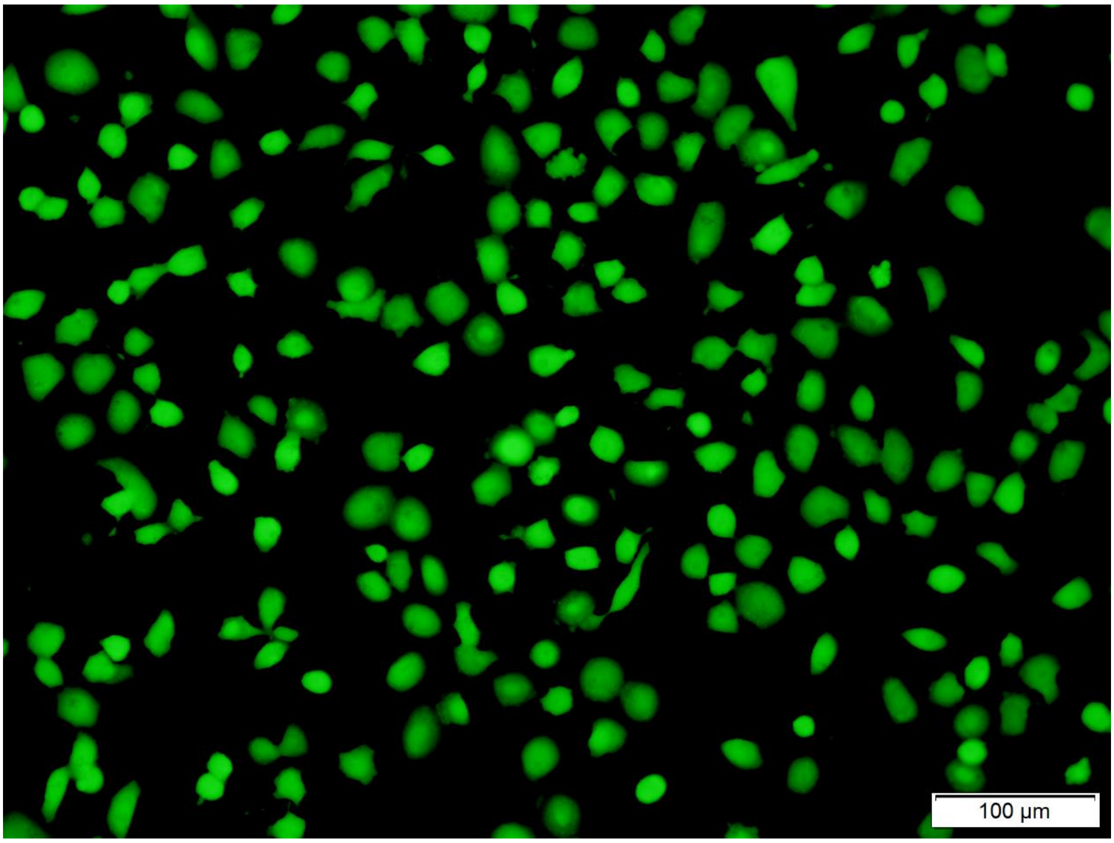

Fig. 7B_A549_ABX_20µM

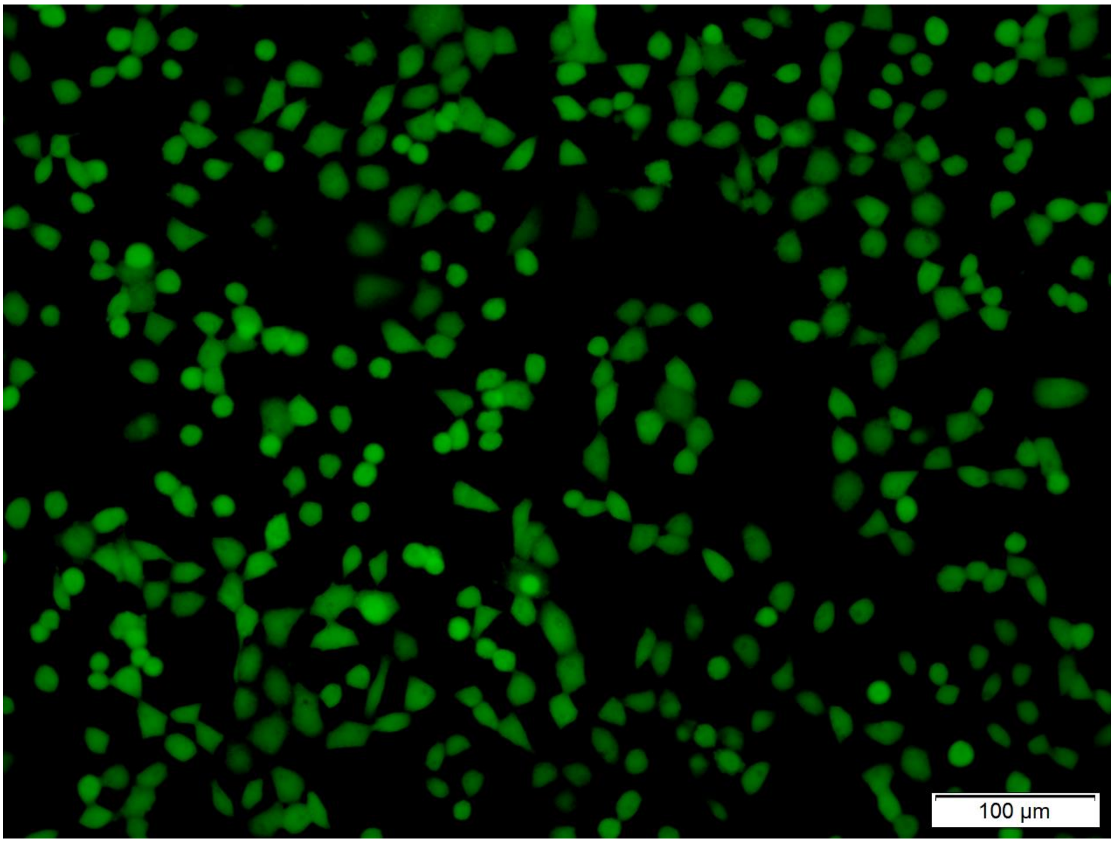

Fig. 7B_A549_ABX+NAC

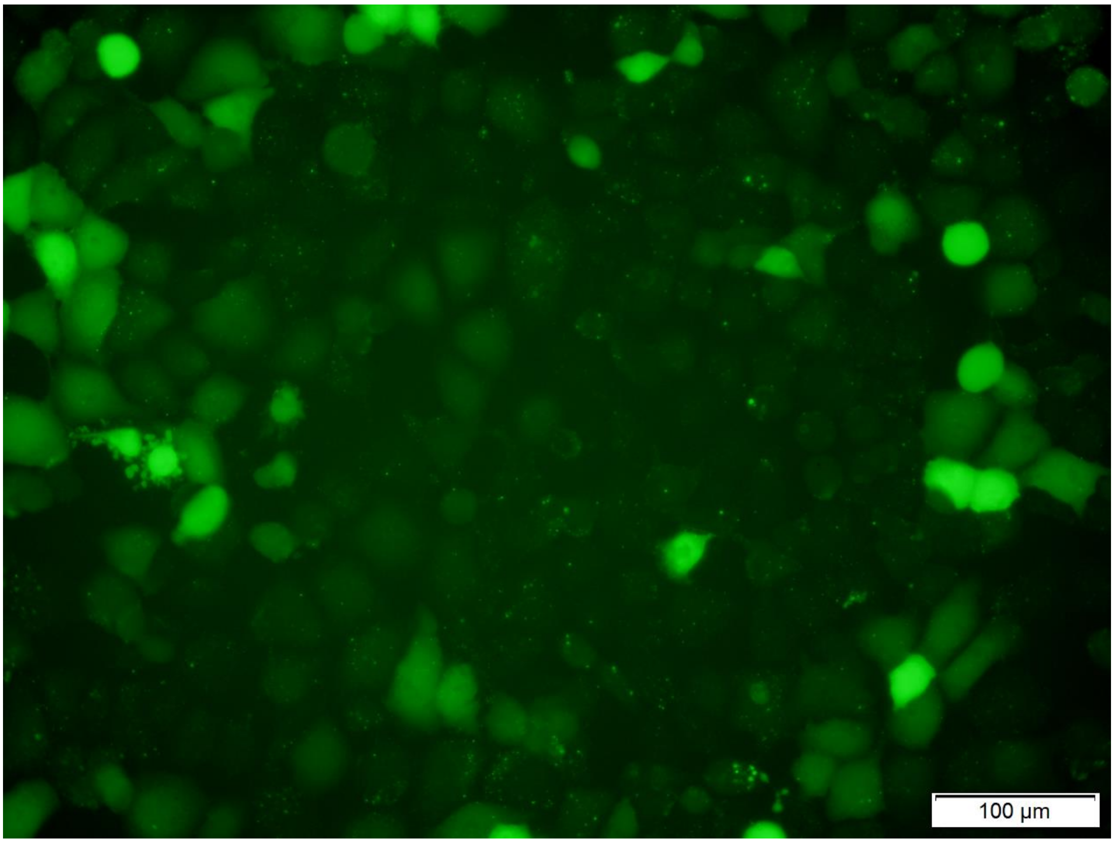

Fig. 7B_H1299_control

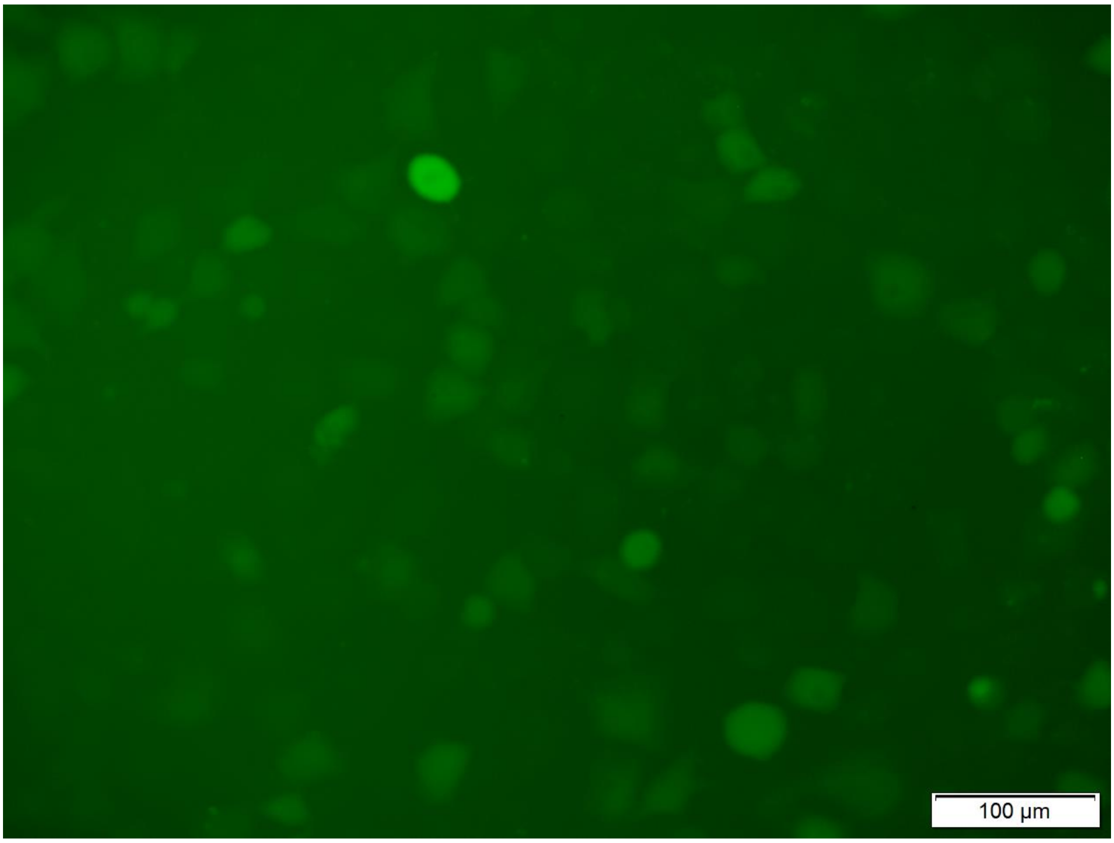

Fig. 7B_H1299_NAC_10µM

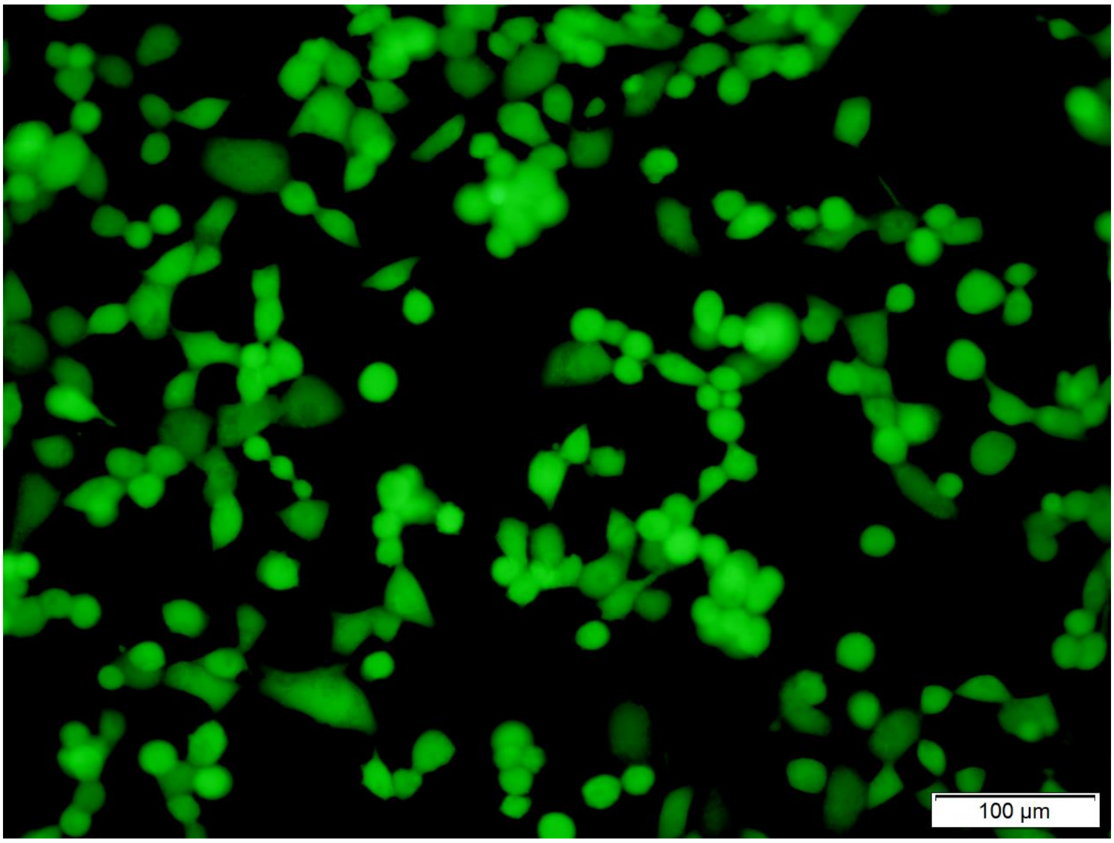

Fig. 7B_H1299_ABX_20µM

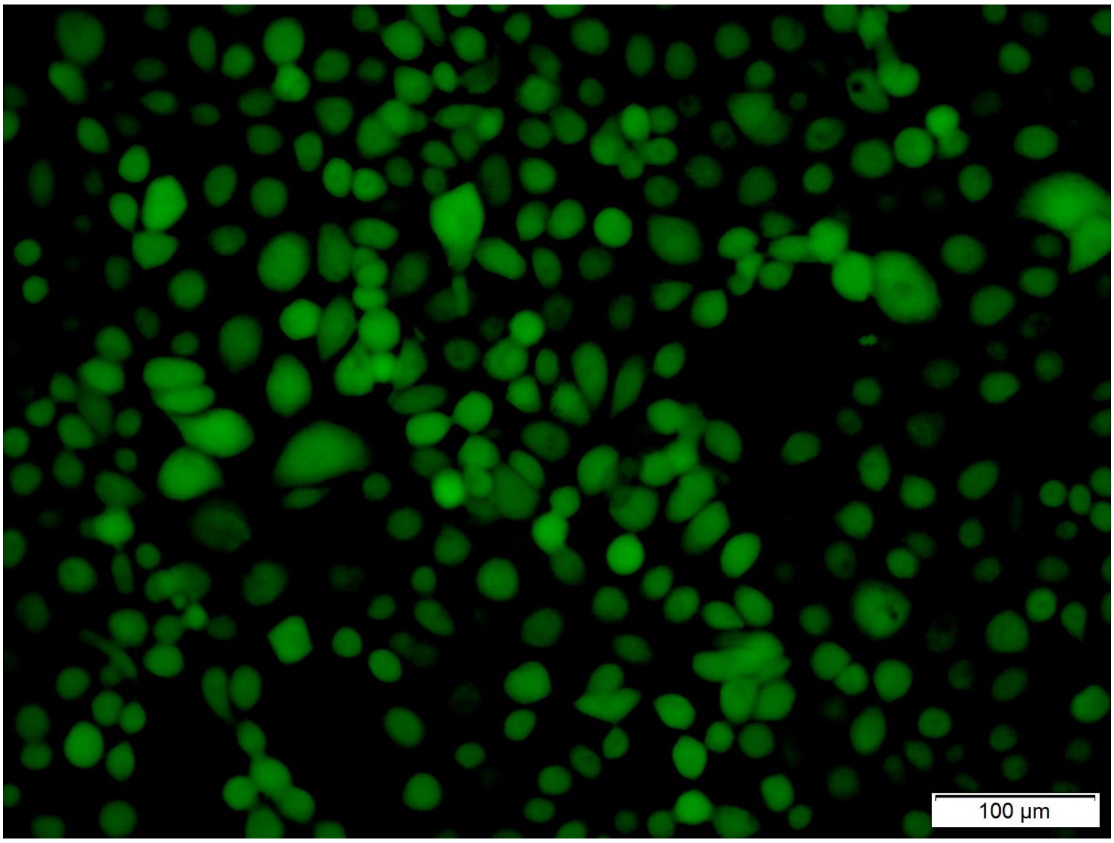

Fig. 7B_H1299_ABX+NAC

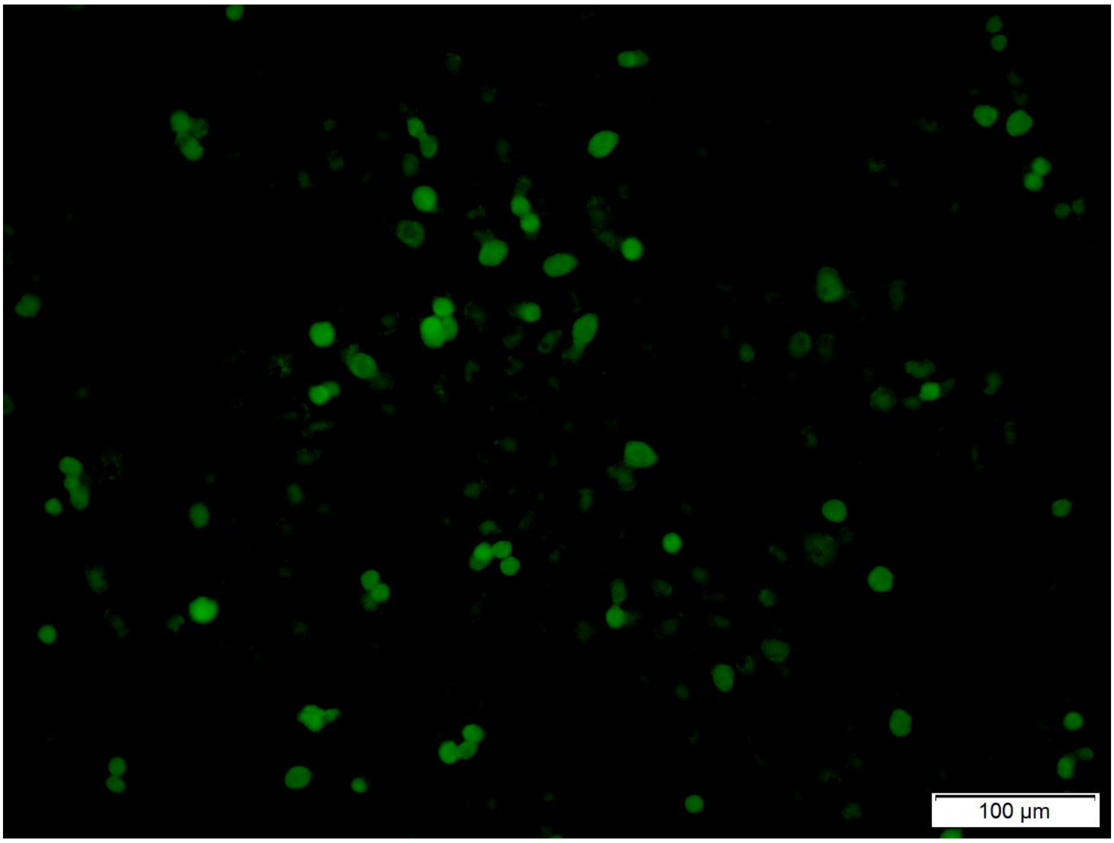

Fig. 7B_H226_control

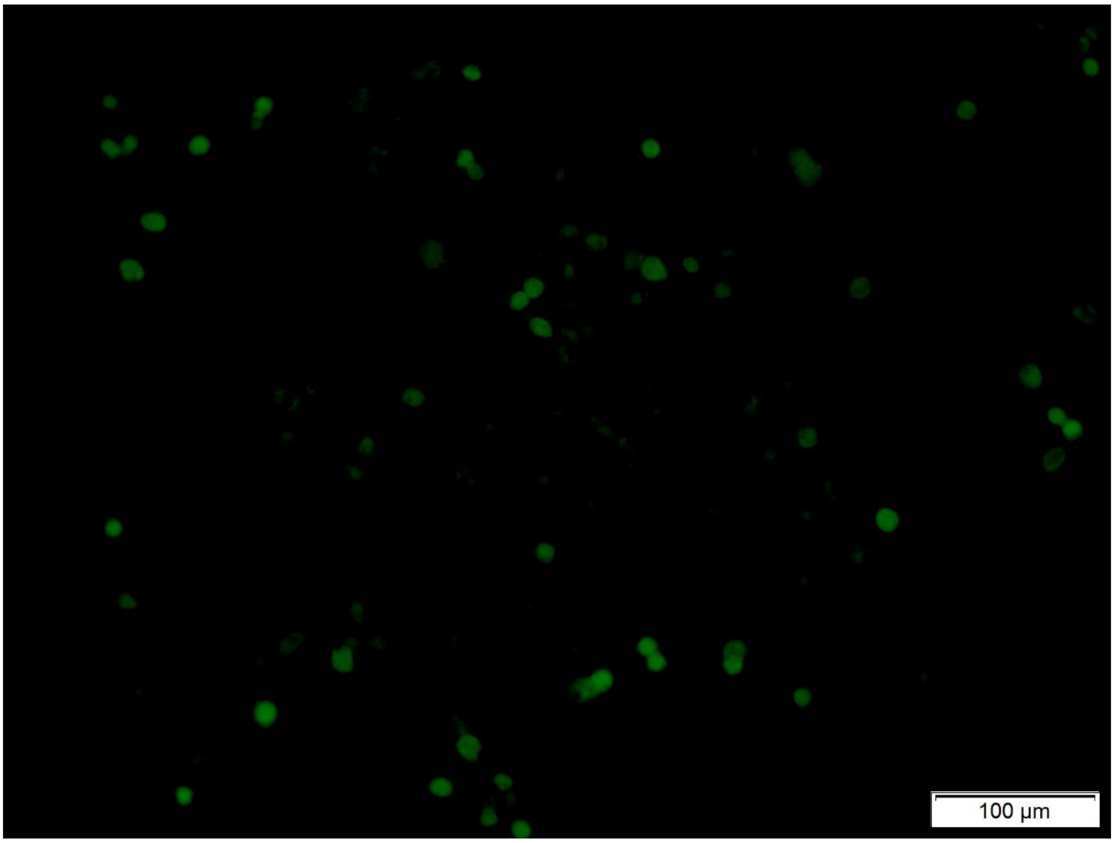

Fig. 7B_H226_NAC_10µM

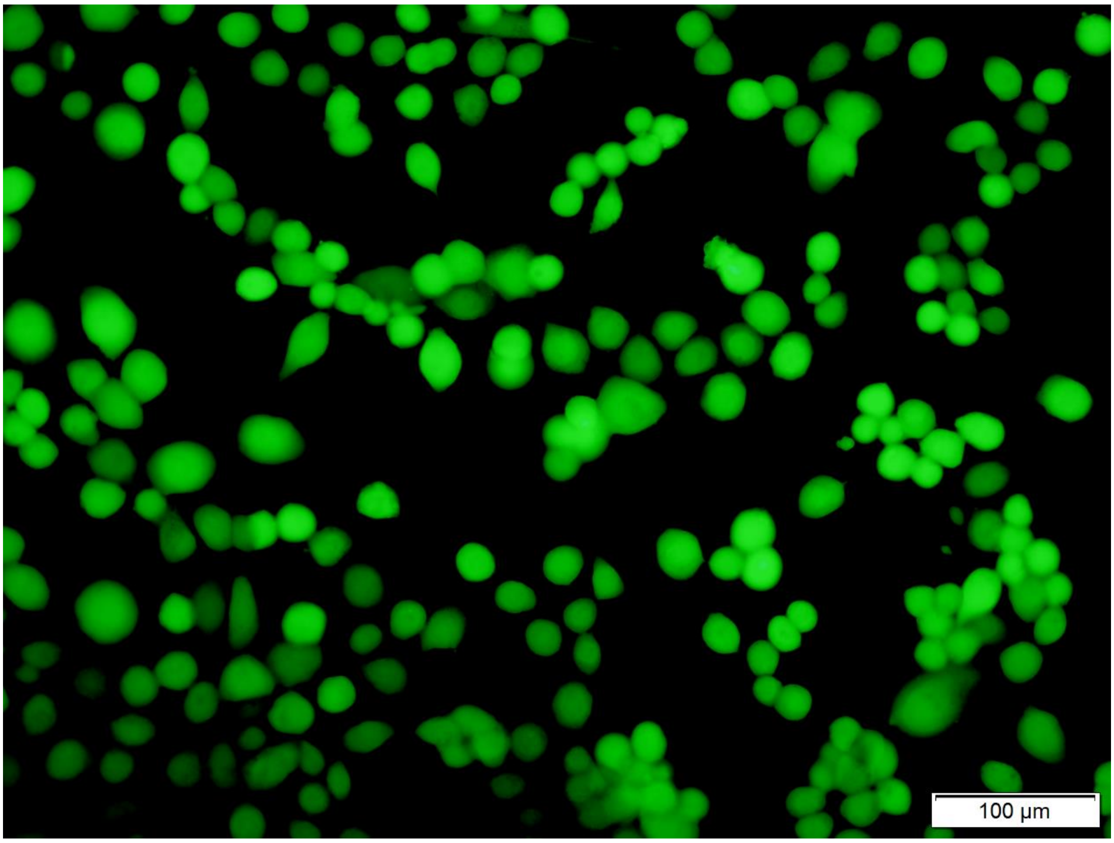

Fig. 7B_ H226_ABX_20µM

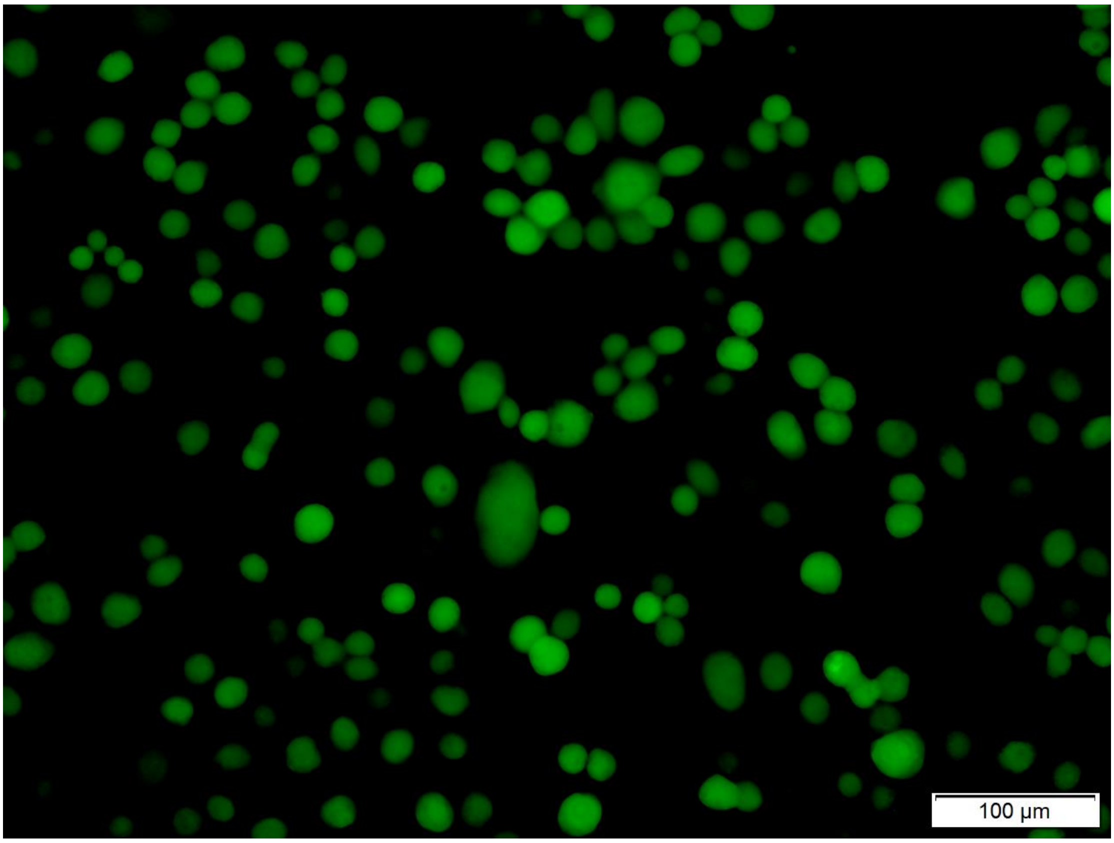

Fig. 7B_ H226_ABX+NAC
